# Thioesterase-mediated side chain transesterification generates potent Gq signaling inhibitor FR900359

**DOI:** 10.1038/s41467-020-20418-3

**Published:** 2021-01-08

**Authors:** Cornelia Hermes, René Richarz, Daniel A. Wirtz, Julian Patt, Wiebke Hanke, Stefan Kehraus, Jan Hendrik Voß, Jim Küppers, Tsubasa Ohbayashi, Vigneshwaran Namasivayam, Judith Alenfelder, Asuka Inoue, Peter Mergaert, Michael Gütschow, Christa E. Müller, Evi Kostenis, Gabriele M. König, Max Crüsemann

**Affiliations:** 1 Institute of Pharmaceutical Biology, University of Bonn, Nussallee 6, 53115 Bonn, Germany; 2 Pharmaceutical Institute, Pharmaceutical & Medicinal Chemistry, University of Bonn, An der Immenburg 4, 53121 Bonn, Germany; 3 University of Paris-Saclay, CEA, CNRS, Institute for Integrative Biology of the Cell (I2BC), Avenue de la Terrasse, Gif-sur-Yvette, 91198 France; 4 Institute for Agro-Environmental Sciences, National Agriculture and Food Research Organization (NARO), Tsukuba, 305-8604 Japan; 5 Graduate School of Pharmaceutical Sciences, Tohoku University, Sendai, 980-8578 Japan

**Keywords:** Biosynthesis, Natural products, Peptides, Medicinal chemistry

## Abstract

The potent and selective Gq protein inhibitor depsipeptide FR900359 (FR), originally discovered as the product of an uncultivable plant endosymbiont, is synthesized by a complex biosynthetic system comprising two nonribosomal peptide synthetase (NRPS) assembly lines. Here we characterize a cultivable bacterial FR producer, enabling detailed investigations into biosynthesis and attachment of the functionally important FR side chain. We reconstitute side chain assembly by the monomodular NRPS FrsA and the non-heme monooxygenase FrsH, and characterize intermolecular side chain transesterification to the final macrocyclic intermediate FR-Core, mediated by the FrsA thioesterase domain. We harness FrsA substrate promiscuity to generate FR analogs with altered side chains and demonstrate indispensability of the FR side chain for efficient Gq inhibition by comparative bioactivity, toxicity and docking studies. Finally, evolution of FR and side chain biosynthesis is discussed based on bioinformatics analyses. Side chain transesterification boosts potency and target affinity of selective Gq inhibitor natural products.

## Abstract (web summary)

FR900359 (FR) is a Gq protein inhibitor depsipeptide isolated from an uncultivable plant endosymbiont and synthesized by non-ribosomal peptide synthetases. Here, the authors discover a cultivable bacterial FR producer and show that FrsA thioesterase domain catalyses intermolecular transesterification of the FR side chain to the depsipeptide core during biosynthesis, improving Gq inhibition properties.

## Introduction

Natural product secondary metabolites comprise a plethora of structurally complex molecules purposed for communication and mediating interactions within or between species^1^. Humans take advantage of natural products, e.g., as medicines or research tools, because of their frequently observed superiority in affinity and selectivity for their targets in comparison to synthetic chemicals. This may be explained by the high likeliness of natural products to cover biologically relevant chemical space^2,3^.

In bacteria, the complete multienzymatic machinery responsible for natural product synthesis is usually encoded in biosynthetic gene clusters (BGCs). Polyketides (PK) and nonribosomal peptides (NRP) are important sources of bioactive and structurally complex natural products, synthesized from monomeric building blocks by assembly-line megaenzymes^4,5^. Nonribosomal peptide synthetases (NRPSs) contain adenylation (A), thiolation (T), and condensation (C) domains, organized into modules, to select and activate the building blocks and to extend the nascent peptide chain^5^. A type I thioesterase (TE), the final domain in canonical NRPS and polyketide synthase (PKS) systems, catalyzes hydrolytic release from the assembly line and, prevalently, the additional formation of macrocyclic scaffolds such as macrolactones and -lactams^6^. However, in recent years, individual type I TEs catalyzing noncanonical reactions, e.g., β-lactone formation^7^, double bond formation^8^ or epimerization^9^ were characterized. While the vast majority of NRPs and PKs are synthesized by one system with a final type I TE, few characterized natural products, e.g., rimosamide^10^ and the hybrid NRP/PK metabolites necroxime^11^ and salinamide^12^ are assembled by the joint action of two NRPS/PKS systems. Here, both systems possess a terminal TE domain, suggesting an intermolecular transesterification reaction conjugating two separately assembled molecules to the final natural product. This paradigm for TE functions was first explored for Sln9_TE_ from the salinamide BGC, shown to catalyze the conjugation of an acylglycine moiety to a cyclic octapeptide intermediate^12^.

**Fig. 1:**
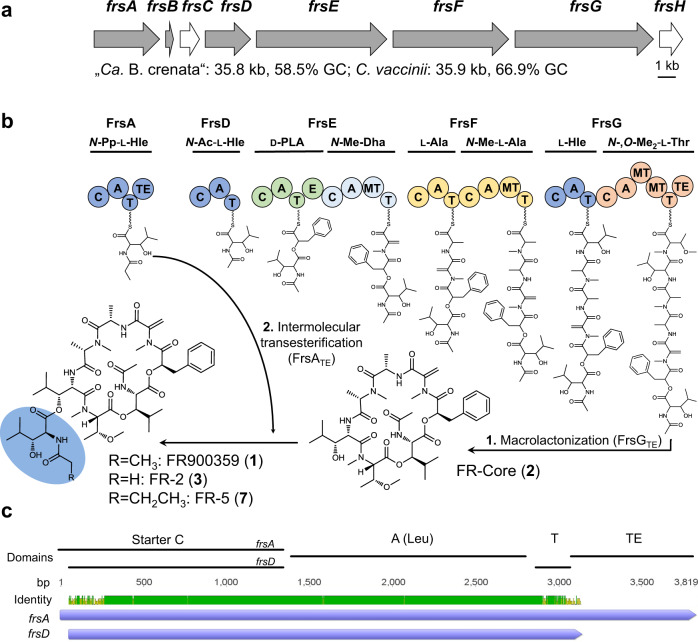
FR biosynthesis. **a** Organization, size and GC content of the *frs* BGCs from “*Ca*. B. crenata” and *C. vaccinii* (gray = NRPS, white = modifying enzyme). **b** Biosynthetic pathway of **1**. First, the NRPS FrsDEFG together with FrsH forms a seven-membered linear peptide chain which is then hydrolyzed and cyclized by FrsG_TE_ to **2**. Then, FrsA_TE_ catalyzes intermolecular transesterification of *N*-Pp-Hle synthesized by FrsA_CAT_ and FrsH (see Fig. 3a) onto **2** to yield the final product **1** (or **3**). Pp = Propionyl, Ac = Acetyl, Me = Methyl, Hle = l-3-Hydroxyleucine, PLA = Phenyllactic acid, Dha = Dehydroalanine. Domains are abbreviated as: C = condensation A = adenylation, T = thiolation, E = epimerization, MT = methyl transferase. Modules are colored according to their activated amino acid precursors. **c** Nucleotide alignment of *frsA* and *frsD*. All predicted domains and identity are indicated. Prediction of domains was conducted with InterPro (EMBL).

FR900359 (FR) (**1**, Fig. 1), first isolated in 1988 from the tropical plant *Ardisia crenata*^13^, is a cyclic depsipeptide containing several modified building blocks, among them three 3-hydroxyleucine (Hle) residues. This natural product family furthermore comprises the analogs FR1-4, mostly varying in length of Hle acyl chains^14^ as well as YM-254890 (YM) and analogs thereof. YM is structurally closely related, containing one less bulky residue in the macrocycle (Thr vs. Hle)^15^. FR is the most potent inhibitor of heterotrimeric Gq family proteins known to date and interacts with its molecular target with high selectivity^16–18^. Heterotrimeric G proteins are the immediate downstream effectors of G protein-coupled receptors (GPCRs), the largest family of membrane proteins in mammalian cells. Therefore, inhibition of Gq, one of the four major G protein families, by FR is a very efficient strategy to simultaneously shut down the intracellular signaling of multiple GPCRs^16,17^. FR1-4 and YM inhibit Gq with similar potency and selectivity^14^, although YM is slightly less active and has altered binding kinetics characterized by a significantly shorter residence time^19^.

Due to its high selectivity and unique mechanism of action, FR has proven to be a valuable tool to address fundamental pharmacological questions in GqPCR-related research^16,17,20^. Recent studies suggest that FR even holds promise as potential therapy for the treatment of Gq-related diseases such as asthma^21^ or uveal melanoma^22,23^.

Structure-activity relationship (SAR) studies on over 30 natural and synthetic FR and YM analogs revealed that almost the entire molecule is vital for interacting with residues in the Gαq binding pocket and thus constitutes the pharmacophore^18,24–27^. Even subtle structural changes were associated with remarkable drops of FR potency, suggesting that the natural product scaffold is optimal for Gαq inhibition. The specialized metabolite FR was proposed to protect *A. crenata* against predators, supported by killing of insects through comparably strong inhibition of insect Gq^28^.

Recent studies revealed, that the actual producer of **1** is the unculturable bacterial endosymbiont “*Candidatus* Burkholderia crenata”, located in *A. crenata* leaf nodules and harboring the *frs* NRPS BGC (Fig. 1a) on an extrachromosomal plasmid^29^. Based on bioinformatics, heterologous expression, and metabolomics, we have provided a model for FR biosynthesis^28^. Accordingly, FR is assembled on two distinct NRPS systems: a cyclic heptapeptide core (**2**) is assembled by the NRPS FrsD-G, while the side chain *N*-propionyl-3-hydroxyleucine (*N*-Pp-Hle) is synthesized by the monomodular NRPS FrsA (Fig. 1b). This model implies the enzymatic conjugation of the two NRPS products as a final biosynthetic step to yield **1**.

Here we discover and characterize a cultivable and genetically tractable bacterial FR producer, enabling the detailed study of FR biosynthesis in vivo and in vitro. We dissect side chain biosynthesis and demonstrate, that the FrsA TE domain catalyzes intermolecular transesterification of the FR side chain to the cyclic depsipeptide intermediate FR-Core (**2**). This modification substantially improves inhibition of and affinity to Gq, as shown by comparative analyses. Moreover, we harness the enzymatic machinery to produce FR analogs with altered side chains in vitro and in vivo and perform bioinformatics analyses in order to identify related BGCs and to investigate *frsA* evolution.

## Results

### Characterization of a cultivable FR producer

To test our biosynthetic model, we aimed at studying FR biosynthesis in vivo. Practical use of our heterologous expression system in *Escherichia coli* was hampered by very low titers^28^, which could not yet be enhanced despite multiple efforts. Thus, to identify cultivable FR producing bacteria, we searched for *frs* BGC fragments in the available databases. Using BLAST analyses, we found short DNA sequences with high similarity to parts of the *frs* BGC in *Chromobacterium vaccinii*. The identified Gram-negative, rod-shaped bacterium was previously isolated from soil and roots of cranberry plants^30^. Subsequent cultivation of *C. vaccinii* in LB medium and extraction with *n*-butanol led to the detection and isolation of **1** (2.5 mg/L, Supplementary Fig. 1) and other derivatives previously identified from *A. crenata*. In contrast to *A. crenata*, the titer of the structural analog FR-2 (**3**, Fig. 1b)^14^, containing the altered side chain *N*-acetyl-3-hydroxyleucine (*N*-Ac-Hle), was almost as high as that of **1** (Supplementary Fig. 2), suggesting lower availability of propionyl-CoA in *C. vaccinii* or increased promiscuity of the responsible biosynthetic enzymes. Using MS/MS-based GNPS molecular networking^31^, we analyzed the molecular family of **1** produced by *C. vaccinii* in LB and M9 minimal medium and compared it to the one produced in *A. crenata*^14^. We observed a different profile of FR congeners in *C. vaccinii* depending on the culture medium (Supplementary Fig. 3), opening up possibilities to discover FR derivatives with altered biological properties.

Next, we aimed at fully sequencing the *frs* BGC in *C. vaccinii*. A draft genome containing eleven (short) contigs with *frs* BGC identity was published in 2015^32^. As this sequence was insufficient to determine the total number of genes within the cluster and their structural organization, we performed another Illumina genome sequencing of *C. vaccinii*. This still did not yield a full *frs* sequence due to the highly identical regions within the BGC, but reduced the number of contigs to six. Assuming a similar organization of the *frs* BGCs from *C. vaccinii* and “*Ca*. B. crenata”^28^, we attempted to close the five remaining gaps by employing a PCR-based approach (Supplementary Fig. 4). Ultimately, this enabled assembly of the complete *C. vaccinii frs* BGC, which is identical to the one from “*Ca*. B. crenata” in terms of gene number, organization and domain architecture (Fig. 1a, Supplementary Tables 2, 3). Only slight differences in GC content (58.5% vs. 66.9%) and the length of intergenic regions (“*Ca*. B. crenata”: 120 bp, *C. vaccinii*: 159 bp) were observed. In both *frs* BGCs, the sequences of *frsA*_*CAT*_ and *frsD*, both involved in the synthesis of acylated hydroxyleucines, are almost identical (94.3%) (Fig. 1c, Supplementary Fig. 5, 6).

### *frsA* knockout yields the key intermediate FR-Core

**Fig. 2:**
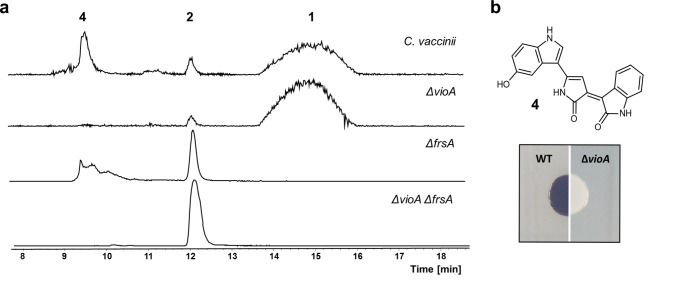
Characterization of *C. vaccinii* knock-out mutants. **a** HPLC-MS with extracted ion chromatograms (EIC) of *m/z*: 1002.539 (**1**), *m/z*: 817.430 (**2**) and *m/z*: 344.101 (**4**, violacein) for butanolic extracts of *C. vaccinii* and the Δ*vioA*, Δ*frsA* and Δ*vioA*/*frsA* deletion mutant strains. **b** Colony phenotype of wild type (WT) *C. vaccinii* changes, after deletion of *vioA*, from purple (production of **4**) to white.

To confirm experimentally the proposed biosynthetic pathway, we aimed at constructing an *frsA* deletion mutant of *C. vaccinii*. For this, we employed the FRT cassette from pPS858, which has been developed for the construction of *Pseudomonas* deletion mutants^33^. In a first step, a 3708 bp internal region of *frsA* was replaced with the FRT cassette yielding the strain *C. vaccinii* Δ*frsA*::FRT. To avoid any polar effects on downstream genes, the 3′-end of *frsA* (51 bp) was left intact. Then, the FRT cassette was removed from the genome by Flp-mediated site-specific recombination, leaving only an 86 bp scar at the site of integration (Supplementary Fig. 7). This resulted in the strain *C. vaccinii* Δ*frsA*. Integration and loss of the FRT cassette during this process were verified by site-specific amplification of the *frsA* locus (Supplementary Fig. 8). Analysis of the butanolic extract of *C. vaccinii* Δ*frsA* by HPLC-MS revealed the absence of **1**, while the production of **2**, before only found in traces, appeared to be enhanced (Fig. 2a). This supports our biosynthetic hypothesis for FR and confirms the role of **2** as a biosynthetic intermediate.

*C. vaccinii* Δ*frsA* offered, however, limited potential to produce sufficient amounts of **2** for further analyses. We thus sought to optimize this strain by interrupting the biosynthesis of the purple pigment violacein (**4**, Fig. 2b), which might enhance production of **2** and would allow for an easier purification from the crude extract. To stop production of **4**, *vioA*, catalyzing the first step of violacein biosynthesis^34^, was deleted. Construction of a Δ*frsA*/Δ*vioA* double mutant was performed by replacing a 932 bp region of *vioA* with the FRT cassette in the *C. vaccinii* Δ*frsA* mutant as described above. To investigate any effects of the *vioA* deletion on FR production, a Δ*vioA* single mutant was constructed in the same fashion. Both resulting strains showed a white phenotype instead of the purple one reported for the wild type (Fig. 2b)^30^. Remarkably, the Δ*frsA*/Δ*vioA* double mutant produced substantially higher amounts of **2** compared to the wild type or the Δ*frsA* strain (Fig. 2a). This also applies to the production of **1** from a Δ*vioA* strain (Fig. 2a). Subsequently, **2** was isolated and purified in a preparative scale from the Δ*frsA*/Δ*vioA* double mutant strain. MS/MS analyses had indicated **2** to be a derivative of **1** lacking the side chain *N*-Pp-Hle^14,28^. The absence of all NMR signals of *N*-Pp-Hle in both the ^1^H NMR and ^13^C NMR spectra as well as an upfield shifted ^1^H resonance of H-33 (δ 3.42) provided further evidence for this hypothesis. Finally, analyses of the 1D and 2D NMR data unambiguously proved the structure of **2** (Fig. 1b, Supplementary Figs. 9–15, Supplementary Table 4). The configuration of **2** was deduced in analogy to the known absolute configuration of **1**^35^ and in accordance with the biosynthetic pathway (Fig. 1b). We named the compound FR-Core.

### In vitro studies on FrsA to dissect side chain biosynthesis

**Fig. 3:**
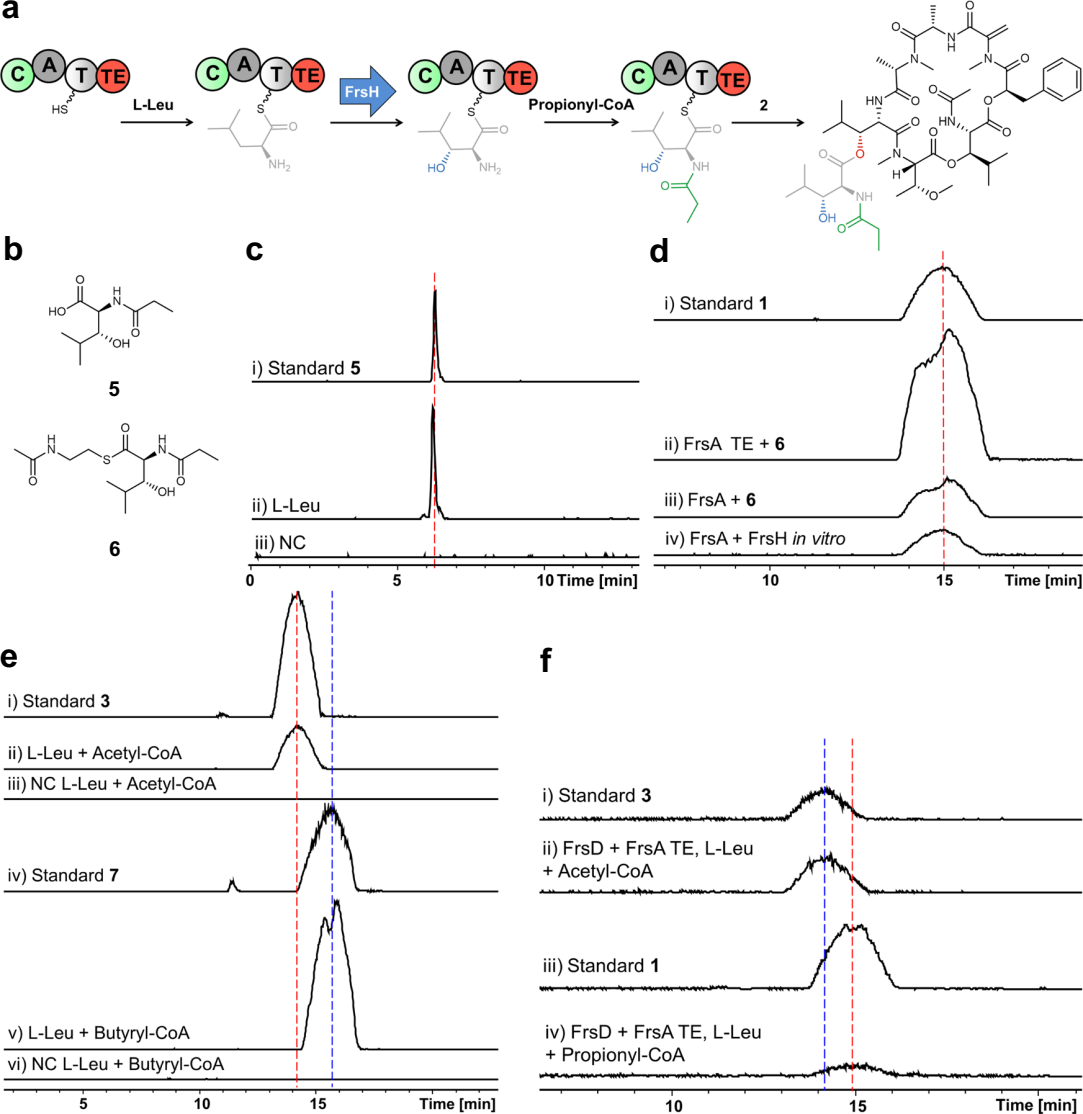
In vitro assays with FrsA/B, FrsH, and FrsA_TE_. **a** Reaction scheme of *N-*Pp-Hle formation and intermolecular transesterification onto **2** to yield **1** Biosynthetic modifications are color-coded according to the respective catalyzing domain. **b** Structures of synthesized *N*-Pp-Hle (**5**) and its SNAC (**6**). **c** In vitro production of **5** (*m/z*: 202.108). (i) Synthetic **5** (1 µg/ml). (ii) Enzymatic assay with purified FrsA_CAT_/FrsB, FrsH, incubated with l-Leu and propionyl-CoA, hydrolyzed with KOH. (iii) Negative control (NC) with heat-inactivated protein. **d** In vitro production of **1** (*m/z*: 1002.540). (i) **1** (10 µg/ml). (ii) Purified FrsA_TE_ incubated with **6** and **2**. (iii) Purified FrsA/B incubated with **6** and **2**. (iv) Purified FrsA/B, FrsH incubated with propionyl-CoA, l-Leu and **2**. **e** In vitro production of FR congeners **3** (*m/z*: 988.530) and **7** (*m/z*: 1016.550). (i) **3** (10 µg/ml). (ii) Purified FrsA/B, FrsH incubated with l-Leu, acetyl-CoA and **2**. (iii) Negative control. (iv) **7** (10 µg/ml). (v) Purified FrsA/B, FrsH incubated with l-Leu, butyryl-CoA and **2**. (vi) Negative control. **f** In vitro FrsA/D combinations: (i) **3** (1 µg/ml). (ii) Production of **3** by FrsA_TE_ and FrsD, incubated with acetyl-CoA, l-Leu and **2**. (iii) **1** (1 µg/ml). (iv) Production of **1** by FrsA_TE_ and FrsD, incubated with propionyl-CoA, l-Leu and **2**.

To investigate the assembly of *N*-Pp-Hle (**5**) in vitro (Fig. 3a), the CAT tridomain of FrsA as well as the almost identical FrsD were expressed in *E. coli* BAP1 with a histidine tag and purified via affinity chromatography. Coexpression with the MbtH-like protein FrsB, resulting in coelution and copurification, was critical for FrsA and FrsD solubility and adenylating activity (Supplementary Fig. 16). The γ-^18^O_4_-ATP exchange A domain assay^36^ with the purified proteins showed favored activation of l-Leu. Despite the exact same sequence of both A domains, FrsA_A_ exhibited noticeable broader substrate specificity than FrsD_A_, by also showing moderate activation of the isomers d-Leu and l-Ile (Supplementary Fig. 17). Next, we evaluated the activity of the FrsA C_starter_ domain. Using FrsA_CAT_/FrsB and the substrates l-Leu and propionyl-CoA, acylation of the leucinyl amino group was anticipated. However, only minor traces of propionylleucine were detected after alkaline hydrolysis, suggesting that β-hydroxylation of leucinyl-FrsA_CAT_ had to take place before acylation. By bioinformatics, we previously identified the non-heme diiron monooxygenase FrsH as a candidate to catalyze the hydroxylation reaction^28^. To date, the only characterized member of this enzyme family is CmlA from the biosynthesis of the antibiotic chloramphenicol^37,38^. Although having only 34% sequence identity to CmlA, most residues necessary for the overall structure, the formation of the phosphopantetheinyl substrate binding pocket, and coordination of the diiron cluster are conserved in FrsH^28^. We cloned and expressed FrsH in *E. coli* BL21(DE3) to prove its function as a β-hydroxylase (Supplementary Fig. 16). The complexion of iron by the purified enzyme was shown via UV/Vis spectroscopy, revealing a broad band between 300 and 400 nm, which diminished after transformation to the ferric state with Na_2_S_2_O_4_ in the presence of methylviologen as a chemical electron transmitter (Supplementary Fig. 18). Upon addition of FrsH to the enzymatic assay mixture, we were able to detect **5** after hydrolysis (Fig. 3b, c) and confirmed its identity by comparison with a synthesized standard (see Supplementary Fig. 20), proving the successful assembly of the FR side chain in vitro. We next evaluated the substrate promiscuity of the enzymatic domains by adding different amino acid precursors to the assay. Indeed, reflecting the A domain assay results, LC-MS analyses show the synthesis of altered side chains after the addition of the isomers d-Leu and l-Ile, demonstrating that also FrsH and FrsA_C_ accept alternative substrates (Supplementary Fig. 19).

### FrsA_TE_ catalyzes side chain transesterification to FR-Core

We next turned our attention to the unusual TE domain of FrsA that was hypothesized to catalyze an intermolecular transesterification, i.e., the attachment of the side chain assembled by FrsA onto the free hydroxyl group of FR-Core (**2**). To prove this experimentally, we synthesized **6**, an *N*-acetylcysteamine (SNAC) thioester of **5** (Fig. 3b, Supplementary Figs. 20–24), to mimic the phosphopantetheinyl moiety of the T domain. We overexpressed and purified FrsA_TE_ from *E. coli* BL21 (DE3) and incubated the domain with the substrates **2** and **6**. Indeed, the formation of **1**, the final product of the biosynthetic pathway, was observed in this experiment, thereby unambiguously confirming the proposed function of FrsA_TE_ (Fig. 3d). The same result was observed when incubating the whole overexpressed and purified module FrsA with **2** and **6**. We next incubated purified coexpressed FrsA/FrsB and FrsH with the precursors l-Leu and propionyl-CoA and added **2** to this reaction mixture. This experimental setup gratifyingly also yielded **1** (Fig. 3d), confirming in vitro activity of the whole enzymatic machinery. Next, we sought to utilize the substrate promiscuity of the domains to alter side chain chemistry in vitro. First, propionyl-CoA was exchanged for acetyl-CoA leading to the production of **3** (Fig. 1b), an abundant compound in *C. vaccinii*, underlining the high substrate flexibility of FrsA_C_ (Fig. 3e). After setting up the reaction with the elongated precursor butyryl-CoA, we observed the formation of a compound with a molecular weight increased by 14 Da (Fig. 3e), suggesting an FR derivative with an *N*-butyryl-3-hydroxyleucine side chain, which was supported by MS/MS analysis (Supplementary Fig. 25). We next attempted to obtain this molecule in preparative quantities: Satisfyingly, feeding of butyric acid to *C. vaccinii* resulted in the formation of the same compound, which we termed FR-5 (**7**) (Fig. 1b). **7** was then isolated and characterized with 2D NMR spectroscopy (Supplementary Fig. 26-31, Supplementary Table 5) to unambiguously confirm its structure.

We furthermore tested if FrsA_CAT_ can be replaced by the highly similar FrsD (see Fig. 1c) in vitro and if their domains can functionally interact: We incubated FrsD with FrsA_TE_ in our assay and also observed the formation of **1** (Fig. 3f), confirming the close relationship of FrsA and FrsD. Exchange of propionyl-CoA for acetyl-CoA in this setup led again to the formation of **3**, but interestingly in higher yields than **1**, highlighting the different substrate promiscuities of the FrsA and FrsD C_starter_ domains, despite their very high sequence identities. FrsA_C_ tolerates larger carbon chains, which is also reflected in the FR structure (propionyl vs. acetyl) and the precursor-directed generation of **7**.

### Comparative analyses reveal the advantage of side chain attachment

**Fig. 4:**
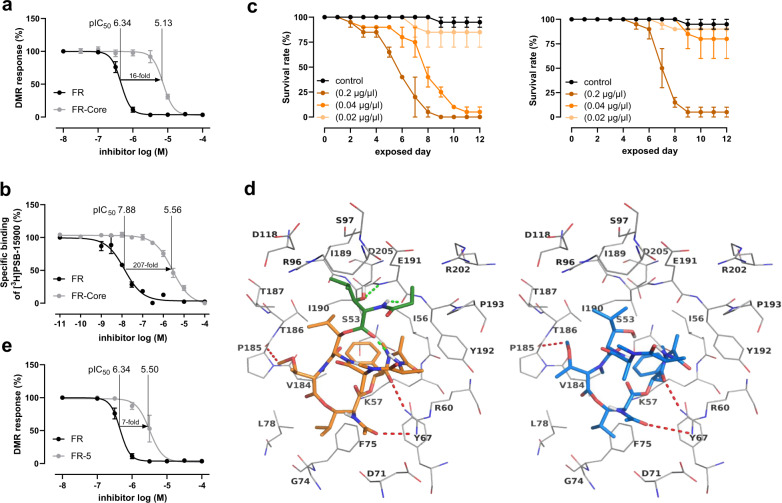
Evaluation of FR-Core and FR-5 compared to FR. **a** Concentration-dependent inhibition of activated Gαq proteins by **1** and **2** as determined by label-free whole-cell DMR biosensing. DMR recordings are representative (mean + s.e.m.) of at least four independent biological replicates conducted in triplicate. **b**. Competition binding experiments of **1** and **2** versus the FR-derived radiotracer [³H]PSB-15900 at human platelet membrane preparation (50 µg protein per vial), incubated at 37 °C for 1 h. **c** Exposure of nymphs of a stink bug (*Riptortus pedestris*) to different concentrations of **1** (left) and **2** (right), survival rate was measured. **d** Docked poses of **1** (left, represented in sticks and colored in orange, the *N*-Pp-Hle group present only in **1** is colored in green) and **2** (represented in sticks and colored in blue) in the binding pocket of the Gαq protein shown as line representation. Some of the interactions common for **1** and **2** are indicated by red dotted lines, and the interactions specific for **1** are shown as green dotted lines. Oxygen atoms are colored in red, nitrogen atoms in blue and polar hydrogen atoms in white. **e** Concentration-dependent inhibition of activated Gαq proteins by **1** and **7** as determined by label-free whole-cell DMR biosensing (see a). Source data are provided as a Source Data file.

Based on earlier SAR studies^18,27^, we reasoned that the side chain of **1** might play an important role in Gq inhibition. Thus, we first compared the Gαq inhibitory activity of **2** in comparison to **1** in CRISPR-Cas9 genome-edited HEK293 cells deficient in Gαq and Gα11 using real-time live-cell phenotypic biosensing based on dynamic mass redistribution (DMR)^39,40^. In our experimental setup, endogenous or overexpressed Gq-coupled receptors (herein: carbachol-activated muscarinic M3 receptor) show activity in DMR assays only upon re-expression of Gαq (Supplementary Fig. 32). We first examined **1** and observed the full inhibition of Gq-activity with an IC_50_ value of 0.45 µM, which correlates well with previously determined inhibitory activities in related assays^41^. Next, we examined **2** and observed significant differences in Gq-inhibition capabilities. Its potency was about 16-fold less than that of **1** (Fig. 4a, Supplementary Fig. 33, Supplementary Table 6). In addition, competition binding assays with **1** and **2** against the radiolabeled FR-derivative [³H]PSB-15900^19^, performed with human platelet membrane preparations, revealed a 207-fold decrease in binding affinity for **2** compared to **1**. Here, the measured *p*IC_50_-value decreased from 7.88 ± 0.09 for **1** to 5.56 ± 0.04 for **2** (Fig. 4b). Despite the discrepancies between binding and function, both experiments clearly underline the vital role of side chain **5** for efficient Gq-inhibition by **1** and suggest an even greater role of the side chain as a determinant for high-affinity binding of **1** to Gq. To investigate ecological relevance of the side chain, we fed **1** and **2** to nymphs of the stinkbug *Riptortus pedestris*: While high concentrations (200 µM) of both metabolites killed all insects after nine days, at 40 µM, only feeding of **1** had lethal effects, while **2** did not affect the animals (Fig. 4c), demonstrating improved in vivo toxicity of **1** compared to **2**.

To get insights into the molecular interaction of **2** to its Gαq binding pocket, a docking-based model of the Gq protein in complex with **2** based on the co-crystal structure of Gαq-βγ bound with YM (PDB ID: 3AH8, resolution: 2.9 Å)^42^ was generated and compared with the model created for **1**. We found that the macrocyclic core structures likely display identical orientation, and the isopropyl groups of both molecules are anchored inside the binding pocket through hydrophobic interactions. In both structures, *N,O*-dimethylthreonine and d-phenyllactic acid are predicted to form hydrogen bond interactions with the side chain of R60 and the main chain of P185, respectively (Fig. 4d). In addition, the side chain **5**, which is absent in **2**, is proposed to form strong interactions (two hydrogen bonds) with the backbone of E191. The intramolecular interactions formed between **5** and *N*-methylalanine likely stabilize the binding conformation of **1** inside the pocket. All these additional interactions are believed to be the reason for the much higher binding affinity of **1**. We finally tested the butyryl analog **7** in our DMR assay and compared it to **1** (Fig. 4e, Supplementary Fig. 33, Supplementary Table 6). Gq inhibition capacity of **7** was sevenfold compromised as compared to that of FR. This finding corroborates the notion that the acyl chain length is another critical determinant for optimal fit into the Gαq binding pocket and, consequently, efficient inhibition of this G protein.

### Global bioinformatics analyses on *frs* and *frsA* evolution

**Fig. 5:**
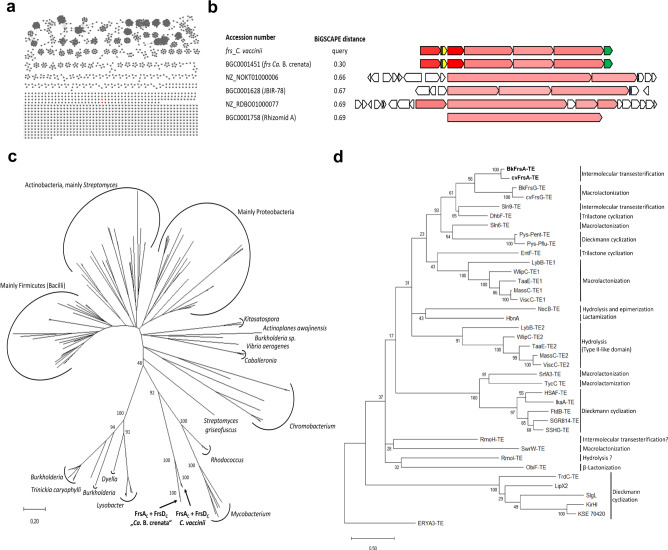
Bioinformatic analyses of the *frs* BGC and selected domains. **a** BiG-SCAPE analysis network of *frs* and 2716 NRPS BGCs, obtained from a global BiG-SLiCE query against all non-fragmented NRPS from the BiG-FAM database (distance of *d*  < 1400) at distance cutoff 0.5. The *frs* BGCs from *C. vaccinii* and “*Ca*. B. crenata” (red) do not cluster with any other BGC. **b** Alignment of *frs* and their 4 closest BGCs from the BiG-SCAPE analysis in Fig. 5a. Color intensity denotes the degree of homology to the respective protein of the *frs* BGC. Red: *frsD* (NRPS), blue: *frsB* (MbtH-like protein), yellow: *frsC* (dehydrogenase), green: *frsH* (hydroxylase). None of the listed BGCs contains homologues to *frsC* or *frsH*. Alignment adapted from Corason^45^. **c** Phylogenetic tree of C_starter_ domains. Taxonomy of clades is indicated. The scale bar represents 20 substitutions per 100 amino acids. **d** Phylogenetic tree of NRPS TE domains. The observed mode of peptide release is indicated. The scale bar represents 50 substitutions per 100 amino acids.

To obtain insights into the evolution of *frs* and side chain biosynthesis, we analyzed relationships of the two architecturally identical *frs* BGCs to other NRPS systems. We therefore employed the BiG-FAM database^43^, which contains over 1.2 million complete and fragmented BGCs based on a global analysis performed with the recently developed BiG-SLiCE tool^44^. We selected all 239,899 non-fragmented NRPS BGCs in BiG-FAM, and then conducted a pairwise Euclidean distance measurement based on the BiG-SLiCE algorithm using a distance cutoff *d* = 1400 (Supplementary Fig. 34). From this procedure, we recovered the 2716 closest matching BGCs to *frs*. These were then subjected to a more sensitive BiG-SCAPE^45^ analysis, also taking synteny into account. Here, no clustering of the *frs* BGCs with other BGCs was observed at a lenient distance cutoff of 0.5 (Fig. 5a). The most closely related BGCs cluster with *frs* at distances between 0.66 and 0.7, and show significant differences in gene and domain architectures and, at best, weak homologies to *frs* genes (Fig. 5b), highlighting the divergence of the *frs* BGCs from all other natural product BGCs currently in the databases.

We then had a closer look at *frsA* evolution: *frsA*_*CAT*_ and *frsD* share an extremely high sequence similarity, particularly a virtually identical (99.9%) 2640 bp sequence stretch encoding the complete A domain and large parts of the C and T domains (compare Fig. 1c, Supplementary Figs. 5, 6). In addition, the A_7_ domain encoding sequence of *frsG* (1634 bp) is also identical to the two abovementioned A domains (Supplementary Fig. 5), indicating more than one duplication event during the evolution of *frs*. We next focused on analyzing the highly similar but not identical C_starter_ domains to shed more light onto their evolution and collected the most similar C_starter_ domains to *frsA*_C_ and *frsD*_C_ from BLAST searches. Subsequent removal of redundant sequences from the list yielded a set of 149 domains (Supplementary Table 7). The phylogenetic analysis shows domains mostly clading according to the taxonomic origin and irrespective of their substrate specificity, which confirms observations from a recent study (Fig. 5c, Supplementary Fig. 35)^46^. All *frs* C_starter_ domains form a deeply rooted clade, supporting their close relationship and highlighting differences to the other domains in the database, even from the same taxa.

Finally, the evolutionary origin of the transesterifying FrsA_TE_ was investigated. Generally, TE domains share low sequence homologies and are known to exhibit broad substrate promiscuities^6^, which is regarded as a prerequisite for the evolution of enzymes to catalysts with altered properties^1,47,48^. Indeed, no domain with high sequence homology was found in the database; its sequence identity to FrsG_TE_, catalyzing macrolactonization of **2**, is 42% (Supplementary Fig. 36). Although TEs show a less-defined phylogenetic relationship with the catalytic reaction type^6,49^, a recent analysis yielded well-separated clades in accordance with the mode of peptide release^50^. When we added all Frs TE domains and others to this set of sequences, a clade emerged in the phylogenetic tree (Fig. 5d, Supplementary Table 8) containing the transesterifying Sln9 and FrsA TEs as well as DhbF_TE_, which catalyzes trimerization in bacillibactin^51^. FrsA_TE_ was grouped closest to FrsG_TE_, indicating an evolutionary relationship of the two TEs from the same BGC. In support, structural model comparisons of FrsA_TE_ and FrsG_TE_ show high overall similarities and only subtle differences (Supplementary Fig. 37).

## Discussion

The cyclic depsipeptide natural product FR (**1**) combines strong inhibition potency and high selectivity for its molecular target Gαq, leading to efficient post-receptor interruption of signal transduction, thereby causing strong effects in the targeted cells and organisms^16^. These properties have made FR a valuable pharmacological tool with promise for therapeutic applications^16,21–23^. Due to a high degree of conservation in its Gαq binding site across the animal kingdom^52^, FR inhibits not only human Gq, but also insect Gq proteins, which may be regarded as ecologically more relevant targets^28^. The FR scaffold has been isolated from diverse sources, such as plant leaf nodules^14^, soil^15^, and a marine ascidian^53^, suggesting the relevance of Gq inhibitors in different ecological contexts. The cultivable bacterium *C. vaccinii* provides an additional source for isolation of **1** and offers opportunities for studying its ecological role in soil. The establishment of a *C. vaccinii* knock-out system, and the subsequent creation of a violacein knock-out mutant, that produces enhanced titers of **1** and derivatives, facilitates our ongoing efforts to fully understand and to engineer FR biosynthesis for the generation of analogs with altered functionalities or G protein specificity.

The obtained experimental data on FrsA and its domains support our biosynthetic model and define **2** as the product of the heptamodular NRPS FrsD-G and substrate for side chain transesterification. A derivative without side chain has also been isolated from the YM-254890 producer^54^, suggesting analogous biosyntheses of the closely related FR and YM.

We have dissected the biosynthetic steps leading to the formation of **1** in vitro. FrsA_CAT_, in concert with the MbtH-like protein FrsB and the monooxygenase FrsH, synthesizes thioesterified *N*-Pp-Hle. Our data imply that leucinyl-FrsA_T_ β-hydroxylation is followed by *N*-acylation. Previous studies on the FrsH homologue CmlA suggest that non-heme diiron monooxygenases are interacting with cognate NRPS domains for amino acid β-hydroxylation, when tethered to their corresponding T domains^37,38^. We are currently investigating the details of this specific protein-protein interaction. SAR studies, exemplified by the far less active analog YM-9^18^, demonstrate that the side chain hydroxyl group is essential for Gq inhibition, emphasizing the importance of the β-hydroxylase FrsH for inhibitor biosynthesis.

The congener FR-2 (**3**) contains *N*-Ac-Hle as side chain and is consistently slightly less potent than **1**, comprising *N*-Pp-Hle^14,27^. Although being almost identical to FrsD_C_, FrsA_C_ prefers larger carbon chains (propionyl vs. acetyl). Further acyl chain extension to a butyryl moiety, exemplified by the characterized analogue **7**, is well tolerated in feeding experiments, but not observed in nature. This modification decreases Gαq inhibitory activity significantly, confirming C_2_ and C_3_ as appropriate acyl chain lengths for effective Gq inhibition.

We have then characterized the conjugation of thioesterified **5** with **2** to yield the final product **1**, catalyzed by FrsA_TE_. The transesterifying TE also interacts with the tridomain FrsD, causing a shift in production yields to **3**, thereby opening possibilities for biosynthetic engineering. In general, such noncanonical intermolecular transesterification reactions provide an opportunity for nature to create depsipeptide ester bonds at PK or peptide side chain hydroxyl functions. A similar reaction has been characterized in the salinamide BGC in a marine *Streptomyces* bacterium, where Sln9_TE_-catalyzed transesterification extends the molecular scaffold and gives rise to the potent antibiotic salinamide A^12^. The specificity of FrsA_TE_ regarding the acceptor substrate of transesterification has yet to be fully explored. In vitro experiments show that the free macrocycle **2** is well accepted and converted to **1**, suggesting **2** as the native substrate, also supported by the accumulation of **2** after *frsA* knockout. However, if the enzyme would also process or even prefer a non-cyclized FR-Core substrate, remains to be determined. The minimal substrate Hle as side chain acceptor yielded no transesterification product in our assay (Supplementary Fig. 38), implying that a larger acceptor substrate is needed for turnover. To this end, a systematic evaluation of diverse cyclic or linear peptide substrates is expected to shed more light on FrsA_TE_ acceptor specificity. Our ongoing structural analysis of FrsA_TE_ should however reveal its active site and mechanistic details of this reaction, which may guide further exploitation efforts of this enzyme for chemoenzymatic purposes.

The side chain attachment boosts the bioactivity of the depsipeptide scaffold, as shown by comparative activity and affinity tests of **1** and **2** on Gαq proteins and insect toxicity assessments. This improvement may be explained by the accompanied docking studies revealing that the extension of chemical space generates additional interactions with the target, as well as intramolecular interactions, thereby highlighting the enormous importance of the side chain for effective Gq inhibition by **1**.

The evolution of natural product BGCs is characterized by both evolution of enzyme promiscuities and genetic events such as horizontal gene transfer, duplications, recombination, and gene expansions^55,56^. We found that in both *frs* BGCs, the regions encoding the leucine activating A domains of *frsA*, *frsD* and *frsG* are identical in addition to large parts of *frsA*_C_ and *frsD*_C_. The close evolutionary relationship of all C_starter_ domains is further supported by a detailed phylogenetic analysis. It is thus conceivable that the evolution of FR side chain biochemistry involves at least one duplication event, leading to biosynthesis of another acylated Hle moiety. TE phylogenetic analysis additionally suggests a close evolutionary relationship of the transesterifying FrsA_TE_ and the macrocylizing FrsG_TE_, all together pointing at the evolution of all parts of *frsA* from ancestors within the BGC.

It has been suggested, that some specialized microbial natural products may have evolved from ancestral metabolites, that had once been the end product of a biosynthetic pathway^1,47^. These ancestor molecules may have served as templates for structural variations based on evolutionary processes^47^. Bioinformatics studies, such as the reconstruction of the evolutionary history of the large natural product families of glycopeptides^57^ and type II PK^58^, support this theory. Based on our analyses, it is tempting to speculate that the intermediate macrocycle **2** is the product of the ancestral BGC *frsB-H*, that was extended by duplication, gene expansion, and further domain evolution resulting in biosynthesis and incorporation of the acylated Hle side chain, thereby encoding a metabolite with improved Gq inhibition potency. This hypothetical model would be consistent with the recently published dynamic chemical matrix evolution (DCME) hypothesis for natural products evolution^1^, when considering improved Gq inhibition properties of the resulting metabolite as fitness advantage for the producer and trait for positive selection of the altered BGC. Yet, this appealing hypothesis remains to be addressed experimentally. Other scenarios for *frsA* evolution, such as the reverse duplication of a previously existing *frsA* to yield *frsD*, its de novo generation or its horizontal acquisition from another organism have thus at this stage also to be taken into account and cannot be excluded with certainty.

To conclude, our global BGC analysis revealed no closely related NRPS system in the sequence databases, coining the two *frs* BGCs a rare, small and so far uniform gene cluster family that was likely shaped by intra-BGC evolution to produce a highly specialized metabolite. Sequencing and comparative analyses of more Gq inhibitor depsipeptide BGCs, such as those of YM and sameuramide, that await discovery, is expected to reveal more details on the evolution of this fascinating family of natural products.

## Methods

### Cultivation and extraction of *C. vaccinii*

For the cultivation of *Chromobacterium vaccinii* MWU205, two different media (LB and M9) were tested for their influence on the production of FR and congeners. *C. vaccinii* was grown both in LB medium (tryptone 10 g/L, yeast extract 5 g/L, and NaCl 5 g/L), and M9 minimal medium (33.7 mM Na_2_HPO_4_, 22 mM KH_2_PO_4_, 8.55 mM NaCl, 9.35 mM NH_4_Cl, 0.4% glucose, 1 mM MgSO_4_, 0.3 mM CaCl_2_, 4.093 mM biotin, 2.965 mM thiamine HCl, 134 µM EDTA, 31 µM FeCl_3_, 6.2 µM ZnCl_2_, 0.76 µM CoCl_2_, 0.42 µM CoCl, 1.62 µM H_3_BO_3_, and 0.081 µM MgCl_2_), both media supplemented with 50 µg/mL carbenicillin to avoid contamination. Cultivation was performed at 25 °C and 200 rpm for 2 d with samples being taken for extraction every 12 h. For LB/M9 medium, 50 mL of culture were 2× extracted with 50 mL *n*-butanol by shaking, phase separation for 24 h and subsequent centrifugation (1800 × *g*, 5 min).

### HPLC-MS/MS analysis

Mass spectra were recorded on a micrOTOF-QII mass spectrometer (Bruker) with ESI-source coupled with a HPLC Dionex Ultimate 3000 (Thermo Scientific) using an EC10/2 Nucleoshell C_18_ 2.7 µm column (Macherey-Nagel) at 25 °C. MS data were acquired over a range from 100 to 3000 *m/z* in positive mode. Auto MS/MS fragmentation was achieved with rising collision energy (35–50 keV over a gradient from 500 to 2000 *m/z*) with a frequency of 4 Hz for all ions over a threshold of 100. HPLC (flow: 0.3 mL/min) conditions start with 90% A (H_2_O + 0.1% AcOH) and 10% B (acetonitrile + 0.1% AcOH). A gradient over 20 min to 100% B starts after 1 min. 5 µl of a 1 mg/mL sample solution (MeOH) were injected.

### Molecular networking

All MS/MS data were converted to.mzXML file format using the software Bruker Compass DataAnalysis 4.2 (Bruker Daltonik GmbH). The data were transferred onto the GNPS server (gnps.ucsd.edu) and molecular networking was performed via the GNPS data analysis workflow using the spectral clustering algorithm^31^, using a parent mass and MS/MS fragment ion tolerance of 0.02 Da. Consensus spectra that contained less than two spectra were discarded. A network was created where edges were filtered to have a cosine score above 0.7 and more than six matched peaks. Results were visualized in Cytoscape 3.7.1 (cytoscape.org) using the solid layout. Identified ions for FR and derivatives, when already known and published, are indicated in the respective nodes.

### Isolation and structure elucidation of FR-Core (2) and FR-5 (7)

NMR spectra were recorded either on a Bruker Ascend 600 NMR spectrometer with a Prodigy cryoprobe operating at 600 MHz (^1^H) and 150 MHz (^13^C) using acetonitrile-*d*_*3*_ as solvent (Deutero GmbH, 99.8% D) or on a Bruker Avance 300 DPX NMR spectrometer at 300 MHz (^1^H) and 75 MHz (^13^C) using CDCl_3_ as solvent (Deutero GmbH, 99.8% D). NMR spectra were processed using Bruker TopspinVersion 1.3 and MestReNova 8.0.1 software packages. Spectra were referenced to residual solvent signals with resonances at δ_H/C_ 1.93/117.7 for acetonitrile-*d*_*3*_ or δ_H/C_ 7.26/77.0 for CDCl_3_. HPLC was carried out using a Waters HPLC system, controlled by Waters Millennium software, consisting of a 600E pump, a 996 PDA detector, and a 717 plus autosampler or on a Waters Breeze HPLC system equipped with a 1525 µ dual pump, a 2998 photodiode array detector, and a Rheodyne 7725i injection system. For isolation of **2**, the crude material (butanolic extract from 4 L culture of *C. vaccinii* Δ*frsA*/Δ*vioA)* was fractionated on a Grace Reveleris X2 flash chromatography system with integrated evaporative light scattering (ELSD) and UV–Vis detection via a Reveleris C_18_ flash column (220 g, 40 µm). A stepwise gradient solvent system of increasing polarity and a flow rate of 65 mL/min was used starting with 50/50 H_2_O/MeOH for 13 min, then changing to 30/70 H_2_O/MeOH within 1 min and hold again for 13 min. The gradient was then changed within 1 min to 25/75 H_2_O/MeOH and held for 25 min, then within 1 min to 20/80 H_2_O/MeOH, held for 13 min, then within 1 min to 15/85 H_2_O/MeOH and held for 25 min. Finally, the gradient was changed within 1 min to 100% MeOH and held for an additional 10 min. According to the measured ELSD and UV signals, a FR-Core containing fraction was collected at 70 min. Final purification was done by HPLC with a semi-preparative Macherey-Nagel Nucleodur C_18_ column (250 × 8 mm, 5 µm) using an isocratic elution with 19/81 H_2_O/MeOH (flow 2.0 mL/min). Pure **2** was isolated as a white powder (t_R_: 12 min, 5 mg). For the isolation of **7**, M9 medium was supplemented with 20 mM butyric acid and *C. vaccinii* was cultivated 48 h as described above. The crude material (butanolic extract from 4.5 L culture of *C. vaccinii*) was fractionated as described above. A **7** containing fraction was collected at around 80 min. Final purification was done as described above. Pure **7** was isolated as a white powder (t_R_: 25 min, 10 mg).

FR-Core (**2**): white, solid; ^1^H NMR (acetonitrile-*d*_*3*_, 600 MHz) and ^13^C NMR (acetonitrile-*d*_*3*_, 150 MHz) data Supplementary Table 4; (+)-HRESIMS *m/z* 817.4295 [M + H]^+^ (calcd for C_40_H_61_N_6_O_12_, 817.4342).

FR-5 (**7**): white, solid; ^1^H NMR (CDCl_3_ 300 MHz) and ^13^C NMR (CDCl_3_, 75 MHz) data Supplementary Table 5; (+)-HRESIMS *m/z* 1016.5507 [M + H]^+^ (calcd for C_50_H_78_N_7_O_15_, 1016.5556).

### Cloning of frs genes and domains

Oligonucleotide primers were synthesized by Eurofins Genomics. Primers listed in Supplementary Table 1 were used for the amplification of the different regions of *frsA* (encoding either FrsA complete, FrsA_CAT_ or FrsA_TE_) and the genes *frsB*, *frsD* and *frsH* from genomic DNA of *C. vaccinii*. The resulting fragments were cloned into restriction sites of pCDFDuet-1 (*frsB) or* pET28a (all others) via standard ligation cloning. The resulting constructs were isolated from *E. coli* Alpha-Select Silver (Bioline), analyzed via restriction digest and verified by Sanger DNA sequencing. The final plasmids were then transformed into *E. coli* BL21 Star (DE3) (Thermo Scientific Fisher) or *E. coli* BAP1 via heat shock for overexpression of recombinant hexahistidine-tagged proteins.

### Protein expression and purification

Expression of constructs including T domains was performed in *E. coli* BAP1^59^, to ensure in vivo phosphopantetheinylation of the T domains. All other constructs were expressed in *E. coli* BL21 (DE3). All constructs containing an A domain were coexpressed with pCDF-Duet-*frsB* to ensure solubility and in vitro activity of the A domain. For the expression of FrsH, TB media was supplemented with 25 µM Fe(III)-citrate. A glycerol stock culture of *E. coli* BL21 (DE3)/BAP1 harboring the selected plasmids was used to inoculate an overnight culture (37 °C, 200 rpm) of LB, supplemented with appropriate antibiotics. This was then used to inoculate 250 mL terrific broth (TB), supplemented with appropriate antibiotics and incubated at 37 °C, 200 rpm for ~4 h (OD_600_ = 0.9), then the culture was chilled on ice. Protein expression was induced upon the addition of 0.4 mM isopropyl-β-d-thiogalactopyranoside (IPTG). Then the cultures were incubated at 16 °C, 200 rpm for a further 16 h. Finally, the cells were harvested via centrifugation (4000 × *g*, 4 °C, 15 min). The pellet was resuspended in 2.5 mL 10 mM imidazole lysis buffer per g pellet (50 mM NaH_2_PO_4_, 300 mM NaCl, pH 8). The cells were lysed on ice with a sonicator in 10-second intervals. The lysate was centrifuged (10,000 × *g*, 4 °C, 10 min). The supernatant was incubated on ice with 1 mL Ni-NTA-agarose (Qiagen). The suspension was kept in light movement. After 1 h, the suspension was filtered with a propylene column (Qiagen 1 mL). The Ni-NTA agarose was washed with 4 mL of a 20 mM imidazole buffer and 4 mL of a 35 mM imidazole buffer and eluted with 2.5 mL of a 250 mM imidazole buffer. All protein fractions were analyzed by SDS-PAGE with a Tris/Tricine buffer system^60^. The elution fraction was rebuffered with PD10 columns (GE). The eluate was concentrated with Vivaspin 500 columns (Sartorius) MWCO 30 kDa or 10 kDa.

### γ-^18^O_4_-ATP-exchange assay for A domains^36^

Two stock solutions were prepared for the assay. Solution 1: 3 mM of the respective amino acid, 15 mM pyrophosphate, 20 mM Tris pH 7.5; solution 2: 3 mM γ-^18^O_4_-ATP (Cambridge Isotope Laboratories), 15 mM MgCl in 20 mM Tris pH 7.5. 2 μL of solution 1, 2 μL of solution 2 and 2 μL of the 5 µM concentrated protein solution (buffer 20 mM Tris, 5 % Glycerol (v/v) pH 7.5) was mixed and incubated at 22 °C for 1.5 h. The reaction was stopped with the addition of 6 μL 9-aminoacridine in acetone (10 mg/mL). The samples were analyzed with MALDI-TOF-MS (Bruker AutoFlex III) in negative mode. Absolute substrate conversion was calculated by dividing the peak area at 506 Da through the combined 506, 508, 510, 512, and 514 Da peak areas. Divided by 83.33 for the molar ratio of the equilibrium between labeled and unlabeled PPi, the % exchange was calculated.

### C_Starter_ domain and hydroxylation assay

Phosphopantetheinylated FrsA was expressed in *E. coli* BAP1 to directly generate the *holo*-form of the T domain^59^, isolated with coexpressed FrsB as described above and buffered in 50 mM Tris pH = 7.5, 25 mM NaCl, 10 mM MgCl_2_ using PD-10 columns (GE) following the manufacturer’s protocol. Assays were performed in a one-pot-reaction: Purified FrsH was reduced with fivefold concentration of Na_2_S_2_O_4_ and equimolar concentration of methylviologen by mixing and incubation on ice for 5 min. To activate the A domain, 1 mM ATP and 1 mM of l-leucine were added. As substrate for the C domain, 500 µM propionyl-S-CoA (CoALA Biosciences, Austin, USA) or acetyl-S-CoA (Sigma) were added. The protein solution of FrsA/B (500 µL, 10 µM) was mixed with 500 µL of the activated FrsH, the resulting concentration of the proteins were 5 µM FrsA/B and 25 µM FrsH. The assay was incubated at 20 °C for 3 h. The reaction was stopped by adding 10% (w/v) TCA and incubated on ice for 30 min. Precipitated proteins were pelleted and washed twice with assay buffer. Alkaline hydrolysis was performed with 0.1 M KOH at 70 °C for 20 min. The solution was lyophilized and the pellet solved in a minimal volume of MeOH for LC-MS analysis. For the negative controls, the proteins were heat-inactivated at 80 °C for 20 min prior to the addition of the substrates.

### TE domain assay

Substrate **2** was dissolved in MeOH to a final concentration of 50 mM, and substrate **6** was dissolved in MeOH to a final concentration of 100 mM. The enzymatic assays were conducted in 50 mM Tris pH = 7.5 and 25 mM NaCl, 10 mM MgCl_2_, 2 mM **6** and 0.5 mM **2** in a total volume of 1 mL. Reactions were initiated by the addition of FrsA_TE_ (5 μM). The assays were incubated at 22 °C for 5 h and then extracted three times with CH_2_Cl_2_. The organic phase was evaporated and the pellet resuspended in 50 μL MeOH. Alternatively, for the in vitro assay, FrsA with coexpressed FrsB and FrsH were used with l-leucine and propionyl-S-CoA to form the side chain under the same conditions as in the C_starter_ domain assay. In addition, 0.5 mM **2** was added and the reaction was incubated and processed like the TE domain assay described above. In this assay mixture the substrates were eventually varied to d-leucine and l-isoleucine for the amino acid and to acetyl-S-CoA (Sigma) and butyryl-S-CoA (CoALA Biosciences, Austin, USA) for the C domain substrate. To test TE acceptor specificity, Hle (Iris Biotech GmbH, Marktredwitz, Germany) was added in varying concentrations (0.5–2 µM) instead of **2** to the assay. For the negative controls, FrsH was heat-inactivated at 80 °C for 20 min prior to the addition of the substrates.

### Label-free dynamic mass redistribution (DMR) assay

Cell culture materials were purchased from Invitrogen (Carlsbad, CA, USA). Generation of genetically engineered HEK293 cells using CRISPR/Cas9 technology to knockout the subunits of Gαq and Gα11 (Gαq/Gα11-null cells) is described elsewhere^16^. Cells were maintained in Dulbecco’s modified Eagle’s medium (DMEM) supplemented with 10% (v/v) fetal calf serum (FCS, PAN biotech, Germany), penicillin (100 units/mL), and streptomycin (0.1 mg/mL), and kept in a humidified atmosphere with 5% CO_2_ at 37 °C. All cell lines were screened by PCR on a monthly basis for mycoplasma contamination and were tested negative. DMR measurements were performed analogous to prior studies^39,40^ using the Epic DMR reader (Corning, NY, USA) together with the Cybi-SELMA semi-automated electronic pipetting system (Analytik Jena AG, Jena, Germany). 24 h after transfection with the G protein αq subunit, HEK293 cells were counted and seeded on 384-well fibronectin-coated biosensor plates at a density of 17,000 cells per well. On the next day, cells were washed twice with Hanks’ buffered salt solution containing 20 mM HEPES (HBSS + HEPES) and incubated for 1 h at 37 °C in the EPIC reader. **1**, **2**, **3** or **7** in HBSS + HEPES were added 1 h before the measurement. The sensor plate was scanned to record a baseline optical signature (no change in basal DMR), and after the addition of compounds, DMR alterations were monitored for 3000 s at 37 °C. All data were analyzed using GraphPad Prism 8.0.0 (GraphPad Inc, La Jolla, CA, USA). Quantification of DMR signals was performed by calculation of the maximum responses. Data points from inhibition curves of individual functional experiments were fitted to a logistic function (Equation 1):

1 $$Y = {\mathrm{bottom}} + \frac{{\left( {{\mathrm{top}} - {\mathrm{bottom}}} \right)}}{{1 + 10^{\left( {{\mathrm{logEC}}_{50} - {\mathrm{x}}} \right){\mathrm{slope}}}}}$$

Concentration-effect curves in Fig. 4a–c were normalized by setting each experimental maximal effect as 100% response. All data are expressed as mean + or ± standard error of the mean (s.e.m.) of at least three independent experiments performed in technical triplicates.

### Competition binding assay

Competition binding assays of **1** and **2** were conducted versus the radiolabeled FR derivative [³H]PSB-15900 utilizing human platelet membrane preparations that natively express Gαq proteins^19^. The experiments were performed in 50 mM Tris, pH=7.4, in a final assay volume of 200 µl. Each assay tube contained 50 µl [³H]PSB-15900 solution in the buffer (final concentration: 5 nM), 50 µl of human platelet membrane preparation in buffer (50 µg of protein), 95 µl of buffer, and 5 µl of **1** or **2** solution, respectively, in varying concentrations dissolved in DMSO. Non-specific-binding was determined in the presence of 5 µM FR dissolved in DMSO. Incubation was performed at 37 °C for 1 h with gentle shaking and was started by the addition of the membrane preparation. The incubation was terminated by rapid filtration through GF/C glass fiber filters using a Brandel 24-well-harvester. Filters were dried, punched out, transferred to scintillation vials, and incubated for at least 6 h in scintillation cocktail (2.5 ml ProSafe FC+®, Meridian Biotechnologies Ltd, UK) prior to measurement in a liquid scintillation counter at an efficiency of 53%. Data were evaluated using GraphPad Prism 7 (GraphPad Inc., San Diego, CA, USA), and IC_50_ values were determined by the One site – Fit logIC_50_-equation.

### Molecular docking

Gαi/qβγ had been co-crystallized with the Gq selective inhibitor YM (3AH8.pdb, resolution 2.9 Å)^42^. The structure was prepared using the structure preparation tool implemented in Molecular Operating Environment (MOE 2019.01; Chemical Computing Group, Montreal, Quebec, Canada, 2019). Afterwards, the hydrogen atoms were assigned according to the Protonate-3D module in MOE 2019.01. **1** and **2** were docked into the binding site of the Gq protein using Autodock 4.2^61^. The atomic partial charges were added using AutoDockTools^62,63^, and three-dimensional energy scoring grids of 60 × 60 ×  60 points with a spacing of 0.375 Å were computed and centered based on the ligand YM. During docking simulations, the ligands **1** and **2** were fully flexible while the residues of the protein were treated as rigid. 50 independent docking calculations using the varCPSO-ls algorithm from PSO@Autodock implemented in AutoDock4.2 were performed and terminated after 500,000 evaluation steps^63^. Parameters of the varCPSO-ls algorithm, the cognitive and social coefficients c1 and c2, were set at 6.05 with 60 individual particles as swarm size. All other parameters were set at their default values. A plausible binding mode of **1** or **2** was selected on the basis of the lowest binding energy and on visual inspection of the interactions.

### Feeding and effect determination of 1 and 2 on insects

A small cotton pad soaked with 500 µl of **1** or **2**-containing water was served to first instar nymphs of the stinkbug *R. pedestris* for oral intake. For feeding, **1** and **2** were each dissolved in 100% MeOH and diluted with water to concentrations of 0.2, 0.04, and 0.02 µg/µl. Distilled water containing 2% MeOH was used as control. Each experiment included 20 stinkbug individuals. Insects were reared in a moisture chamber at 25 °C under a long day regimen (16 h light, 8 h dark), supplied with dried soybean seeds. The **1** or **2**-containing water was exchanged every 3 d, and the survival rate was monitored every day at the same time for 9 d.

### BiG-FAM, BiG-SLiCE, and BiG-SCAPE analyses

A pairwise Euclidean distance measurement based on the BiG-SLiCE algorithm^44^ was done to compare the *frs* BGCs of *C. vaccinii* and “*Ca*. B. crenata” (MiBIG BGC0001451) against all 239,899 non-fragmented NRPS BGCs retrieved from the BiG-FAM database (bigfam.bioinformatics.nl)^43^. Using a distance cutoff *d* = 1400, 2,716 BGCs (Supplementary Data 1) were selected for more sensitive BGC network analysis using BiG-SCAPE^45^ (parameters: --include_gbk_str region BGC --include_singletons --mix --no_classify --clans-off --cutoff 0.5, 0.7 --mode global, using Pfam library version 33.1). The network was visualized with Cytoscape 3.7.1.

### Alignments, structural model, and phylogenetic trees

All sequence alignments of genes and proteins were performed with the online platform EMBOSS needle^64^. The structural models of the TE domains were calculated by the I-TASSER server^65,66^ and aligned with the Swiss-PDB-viewer^67^. For the phylogenetic trees of C and TE domains, protein sequences were retrieved from public databases (for source organisms and accession numbers see Supplementary Tables 7 and 8) and aligned using the MUSCLE algorithm^68^ in MEGA 10^69^. In case of multi-domain proteins, only the C, resp. TE domain was analyzed. With this program, maximum likelihood trees were calculated as follows: The evolutionary history was inferred by using the Maximum Likelihood method and Le_Gascuel_2008 model^70^. The tree with the highest log likelihood (C: -58208.75, TE: -8887.94) is shown. Initial tree(s) for the heuristic search were obtained automatically by applying Neighbor-Join and BioNJ algorithms to a matrix of pairwise distances estimated using a JTT model, and then selecting the topology with superior log likelihood value. A discrete Gamma distribution was used to model evolutionary rate differences among sites (5 categories (+G, parameter C = 1.6801, TE = 1.9188)). The rate variation model allowed for some sites to be evolutionarily invariable ([+I], C: 0.61%, TE: 1.14% sites). The trees are drawn to scale, with branch lengths measured in the number of substitutions per site. C domain analysis involved 149; TE analysis 39 amino acid sequences. For C domain analysis, all positions with <95% site coverage were eliminated, i.e., fewer than 5% alignment gaps, missing data, and ambiguous bases were allowed at any position (partial deletion option). For TE analysis, all positions containing gaps and missing data were eliminated (complete deletion option). There were a total of 409 (C) and 132 (TE) positions in the final datasets.

### Reporting summary

Further information on research design is available in the Nature Research Reporting Summary linked to this article.

## Supplementary information

Supplementary Information

Description of Additional Supplementary Files

Supplementary Data 1

Reporting Summary

## Data Availability

*frs* sequences have been deposited at GenBank, accession number MT876545. MS/MS data are deposited as MassIVE dataset MSV000085975 [gnps.ucsd.edu/ProteoSAFe/result.jsp?task=4cafd85c04a54e5097d0cc78bc1e2be1]. The molecular network can be found under: gnps.ucsd.edu/ProteoSAFe/status.jsp?task=8e524f93dc66405b98c9d77cc0f9c596. The Gq protein structure was retrieved from rcsb.org/structure/3AH8. NRPS BGCs were downloaded from the BiG-FAM database [bigfam.bioinformatics.nl/home]. BGCs used for the BiG-SCAPE analysis are listed in Supplementary data 1. All other data are available from the corresponding author upon request. Source data are provided with this paper.

## Source data

Source Data
