# Supplementary material for: Thioesterase-mediated side chain transesterification generates potent Gq signaling inhibitor FR900359
Source: Nat Commun. 2021 Jan 8;12:144. doi: 10.1038/s41467-020-20418-3 (PMC7794379; doi:10.1038/s41467-020-20418-3)
Supplement: Supplementary file 1 — Supplementary Information [file 41467_2020_20418_MOESM1_ESM.pdf]

# Supplementary Information for

Hermes et al: Thioesterase-mediated Side Chain Transesterification Generates Potent Gq Signaling Inhibitor FR900359, *Nat. Comm.* **2020**

## Content **Page**


**Characterization of *C. vaccinii* as FR Producer** **3**
Supplementary Figure 1: Production curve for FR (**1**) from *C. vaccinii* cultivation in LB 3
Supplementary Figure 2: EIC of FR (**1**) and FR-2 (**3**) from *C. vaccinii n*-butanol extracts 3
Supplementary Figure 3: Molecular network of **1** and its derivatives from different producers 4

**Gap Closure and Bioinformatic Analysis of *C. vaccinii frs*** **5**
Supplementary Table 1: Primers used in this study. 6
Supplementary Figure 4: Gap closure of *C. vaccinii frs* 7
Supplementary Table 2: Annotation of *C. vaccinii* Frs proteins 7
Supplementary Table 3: Comparison of genes and encoded proteins in the *frs* BGCs 8
Supplementary Figure 5: Emboss Needle Alignment of *frsA* and *frsD* in *C. vaccinii.* 7
Supplementary Figure 6: Emboss Needle Alignment of FrsA and FrsD in *C. vaccinii.* 11

**Establishment of Knock-out System in *C. vaccinii* and Creation of Mutant Strains** **12**
Supplementary Figure 7: Schematic representation of the deletion strategy used for *frsA* and *vioA* 13
Supplementary Figure 8: Verification of mutants in *C. vaccinii* 15

**Structure elucidation of FR-Core (2)** **16**
Supplementary Figure 9: Chemical structure of FR-Core (**2**) 16
Supplementary Table 4: $^{1}$H and $^{13}$C NMR spectroscopic data of **2** in acetonitrile-$d_3$ 16
Supplementary Figure 10: $^{1}$H NMR spectrum of compound **2** in acetonitrile-$d_3$ (600 MHz) 17
Supplementary Figure 11: $^{13}$C NMR spectrum of compound **2** in acetonitrile-$d_3$ (150 MHz) 18
Supplementary Figure 12: $^{1}$H-$^{1}$H COSY NMR spectrum of compound **2** in acetonitrile-$d_3$ (600 MHz) 18
Supplementary Figure 13: $^{1}$H-$^{13}$C HSQC NMR spectrum of compound **2** in acetonitrile-$d_3$ (600 MHz) 19
Supplementary Figure 14: $^{1}$H-$^{13}$C HMBC NMR spectrum of compound **2** in acetonitrile-$d_3$ (600 MHz) 19
Supplementary Figure 15: $^{1}$H-$^{1}$H ROESY NMR spectrum of compound **2** in acetonitrile-$d_3$ (600 MHz) 20

***In vitro* Characterization of NRPS Domains and FrsH** **21**
Supplementary Figure 16: SDS-PAGE gels of FrsA, FrsD and FrsH constructs 21
Supplementary Figure 17: $^{18}O_4$-ATP exchange adenylation assay results for $FrsA_{CAT}$ and FrsD. 21
Supplementary Figure 18: Absorption spectra of FrsH. 22
Supplementary Figure 19: EIC (*m/z* = 202.108) of $FrsA_{CAT}$ incubated with D-Leu and Ile 22

**Chemical synthesis of compounds 5 and 6** **23**
Supplementary Figure 20: Synthesis of *N*-Pp-Hle SNAC thioester **6** 24
Supplementary Figure 21: $^{1}$H NMR spectrum of compound **5** in DMSO-$d_6$ (500 MHz) 25
Supplementary Figure 22: $^{13}$C NMR spectrum of compound **5** in DMSO-$d_6$ (125 MHz) 25
Supplementary Figure 23: $^{1}$H NMR spectrum of compound **6** in DMSO-$d_6$ (500 MHz) 26
Supplementary Figure 24: $^{13}$C NMR spectrum of compound **6** in DMSO-$d_6$ (125 MHz) 26

**Structure Elucidation of FR-5 (7)** **27**
Supplementary Figure 25: MS/MS spectrum of FR-5 (**7**) (*m/z*: 1016.55) 27
Supplementary Figure 26: Chemical structure of FR-5 (**7**) 27
Supplementary Table 5: $^{1}$H and $^{13}$C NMR spectroscopic data of **7** in $CDCl_3$ 28
Supplementary Figure 27: $^{1}$H NMR spectrum of compound **7** in $CDCl_3$ (300 MHz). 29
Supplementary Figure 28: $^{13}$C NMR spectrum of compound **7** in $CDCl_3$ (300 MHz). 29

Supplementary Figure 29: $^1$H-$^1$H COSY NMR spectrum of compound **7** in $CDCl_3$ (300 MHz). 30
Supplementary Figure 30: $^1$H-$^{13}$C HSQC NMR spectrum of compound **7** in $CDCl_3$ (300 MHz). 30
Supplementary Figure 31: $^1$H-$^{13}$C HMBC NMR spectrum of compound **7** in $CDCl_3$ (300 MHz). 31

**Gq Inhibition Assays** **32**
Supplementary Figure 32: CRISPR-Cas9 HEK293 Gαq/Gα11-null cells were unresponsive to Carbachol 32
Supplementary Figure 33: Pharmacological characterization of **2** and **7** on Gαq-mediated signaling. 32
Supplementary Table 6: Quantification of FR, FR-Core and FR-5 inhibitory activities 32

**Bioinformatic Analyses on *frs* BGC and FrsA domains** **33**
Supplementary Figure 34: Distances of 239,899 BGC relative to *frs* revealed by a BiG-SliCE analysis. 33
Supplementary Table 7: Information on C domains used for phylogenetic tree 33
Supplementary Figure 35: Phylogenetic tree of NRPS C starter domains 35
Supplementary Figure 36: Alignment of $FrsA_{TE}$ and $FrsG_{TE}$ 36
Supplementary Table 8: Information on TE domains used for phylogenetic tree 36
Supplementary Figure 37: Structural models of $FrsA_{TE}$ and $FrsG_{TE}$ 37

**Discussion: $FrsA_{TE}$ acceptor substrate specificity** **38**
Supplementary Figure 38: EICs of TE assays with the minimal substrate Hle 38

**Supplementary References** **39**

## Characterization of *C. vaccinii* as FR Producer

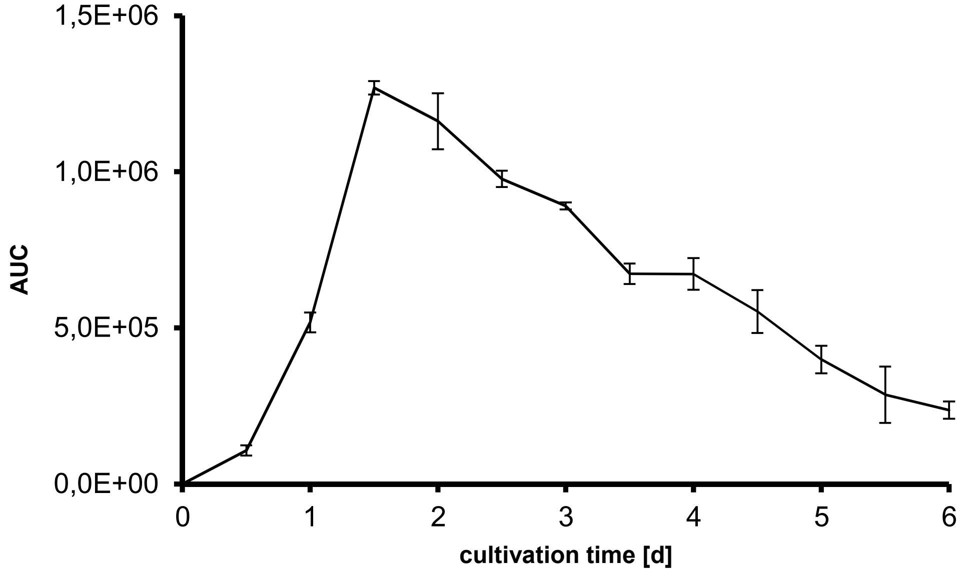


**Supplementary Figure 1: FR production in *C. vaccinii*.** Production curve for FR (**1**) from *C. vaccinii* cultivation in LB. Area under the curve of extracted *m/z:* 1002.54 is plotted. Data are presented as mean values ± SD. Experiments were performed in triplicate.

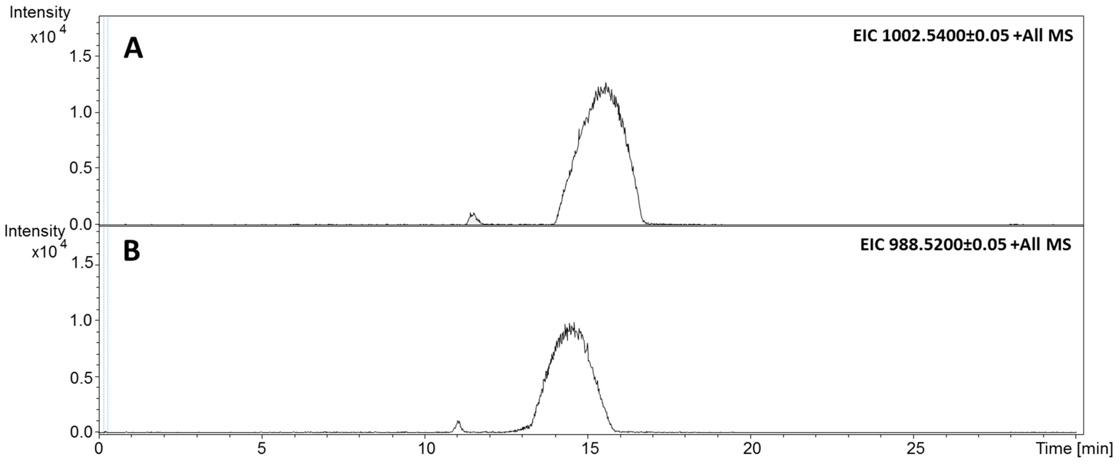


**Supplementary Figure 2: FR and FR-2 comparison.** Extracted ion chromatograms (EIC) of FR (**1**, *m/z*: 1002.54 ± 0.05, A) and FR-2 (**3**, *m/z*: 988.52 ± 0.05, B) from *C. vaccinii n*-butanol extracts. Cultures were grown in LB medium for 36 h.

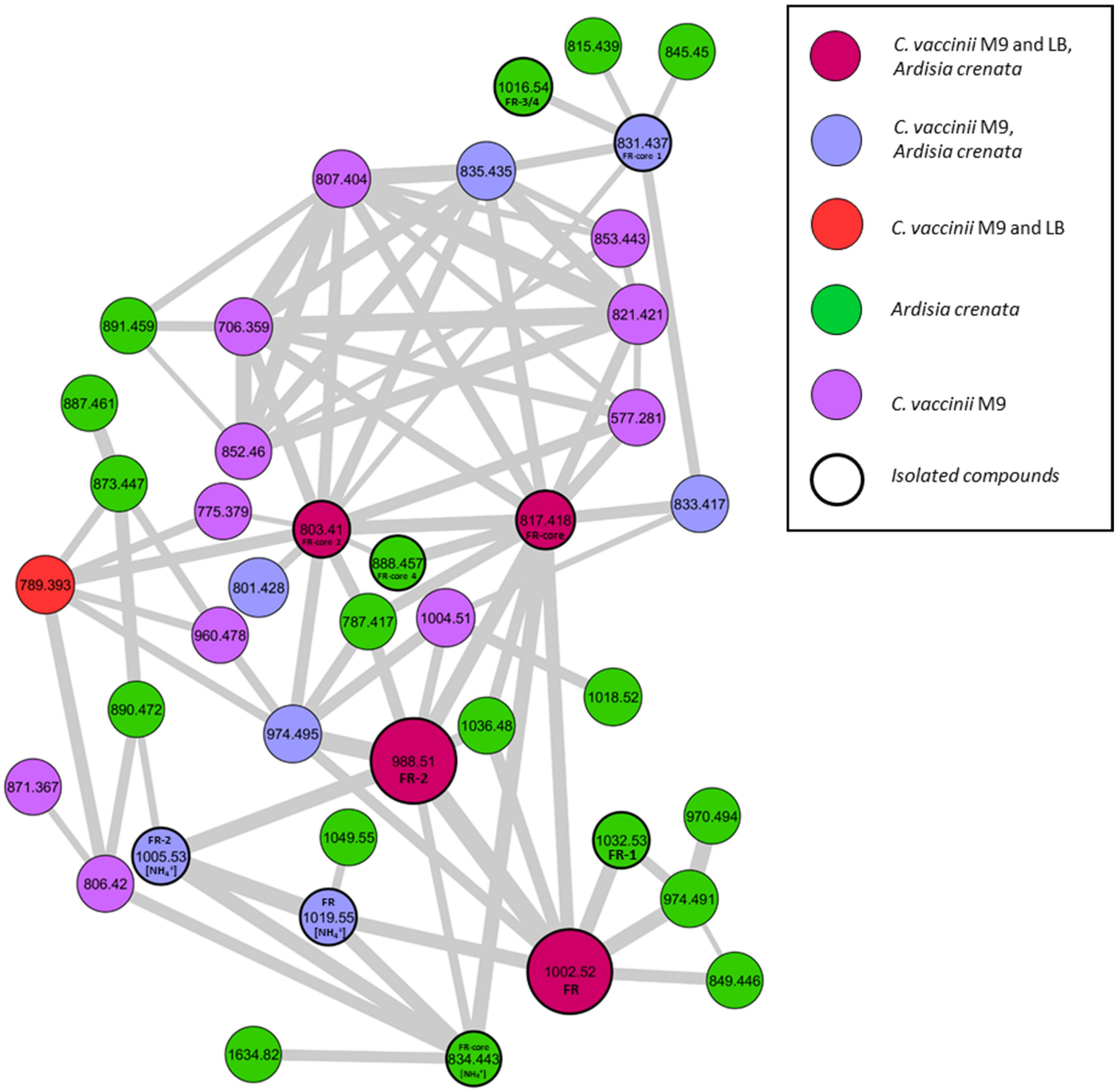


**Supplementary Figure 3: Molecular network of FR and its derivatives from different producers:** *C. vaccinii* in M9 and LB medium and "*Ca.* B. crenata" in *A. crenata*. Nodes display distinct *m/z* features, representing their parent mass and their size represents the number of spectra found. The width of the edges represents the similarity of the fragmentation spectra of the two masses, while the color of the node displays the origin of production (magenta: all extracts; purple: only produced by *C. vaccinii* in M9 medium; green: only produced by *Ca.* B. crenata; red: produced by *C. vaccinii* in LB and M9 medium; blue: produced by "*Ca.* B. crenata" and *C. vaccinii* in M9 medium). Derivatives already known and published are indicated in the respective nodes.

# Gap Closure and Bioinformatic Analysis of *C. vaccinii frs*

## Gap closure of *C. vaccinii frs* BGC

To analyze the genome of *C. vaccinii* MWU205, genomic DNA was prepared from a freshly grown overnight culture with the GenElute Bacterial Genomic DNA Kit (Merck) and sent to Göttingen Genomics Laboratory (G2L, Göttingen) for Illumina sequencing. The resulting draft genome (4.998 Mb in 79 contigs) was then applied to homology guided alignments using the Geneious Pro 5.6.7 software (Biomatters Ltd.), which yielded eight contigs (42, 43, 46, 47, 50, 61, 62, 63) harboring parts of a potential FR gene cluster. When mapped to the *frs* gene cluster of "*Ca*. B. crenata",[1] a potential position for six of them (42, 43, 46, 47, 50, 63) could be determined (Supplementary Fig. 4). For the remaining two contigs, no clear mapping was possible, as they covered reoccurring regions within the *frs* gene cluster (61: A domain identity, 62: MT domain identity). This contig assignment suggested the presence of five gaps. To close these, specific primers binding 130-450 bp upstream of the contig ends were designed (Supplementary Table 1) and applied for PCR-based gap closure. All PCR reactions (25 µL scale) consisted of: 1x Q5 reaction buffer, 1x Q5 GC enhancer, 200 µM dNTPs, 500 nM of forward and reverse primer, 2.25 ng of *C. vaccinii* gDNA and 0.01 U/µL Q5 High-Fidelity DNA polymerase (NEB). Thermal cycling was performed in a Biometra TGradient cycler with an initial denaturation step at 98 °C for 30 s, followed by 30 to 33 cycles of DNA denaturation at 98 °C for 10 s, primer annealing for 20 s and DNA amplification at 72 °C for 30 s/kb amplified, and a final extension step at 72 °C for 2 min. Optimal annealing temperature for specific primer combinations was determined experimentally using temperature gradients from 46–64 °C. PCR products of the expected size were purified and subsequently submitted to terminal-end Sanger sequencing along with the primers used for amplification. Where necessary, primer walking was applied for the sequencing of larger PCR products (see primers in Supplementary Table 1).

**Supplementary Table 1:** Primers used in this study. Restriction sites are bold, Gibson homology arms are underlined. All overhangs are given in lowercase, while target specific sequences are given in uppercase. Restriction sites are bold.

| Name | Sequence (5' → 3') | Description |
|---|---|---|
| BamHI-FRT_for | tga **ggatcc** AGCTTCAAAAGCGCTCTGA | Sequential cloning of FRT into pUC19 |
| SalI-FRT_rev | tgt **gtcgac** GGGGATCTTGAAGTTCCT | Sequential cloning of FRT into pUC19 |
| SphI-frsA-up_for | agt **gcatgc** GGAAAGTACGTCTGGTCTTG | Sequential cloning of the *frsA*-up region into pUC19::FRT |
| SalI-frsA-up_rev | tct **gtcgac** TACATCCAGCTGTGCTGAAG | Sequential cloning of the *frsA*-up region into pUC19::FRT |
| BamHI-frsA-dn_for | ttc **ggatcc** ATTGGTCCTGTTCTCGAGTC | Sequential cloning of the *frsA*-dn region into pUC19::*frsA*-up-FRT |
| SacI-frsA-dn_rev | tga **gagctc** AGTCCCGCATATGATCGATG | Sequential cloning of the *frsA*-dn region into pUC19::*frsA*-up-FRT |
| FRT_for | CGAATTAGCTTCAAAAGCGCTCTGA | One step cloning of FRT into pEX18Tc |
| FRT_rev | CGAATTGGGGATCTTGAAGTTCCT | One step cloning of FRT into pEX18Tc |
| Gib-vioA-up_for | gcatgcctgcaggtcgactctaga**g gatcc** TGACCCTTGGAACAGGATG | One step cloning of *vioA*-up into pEX18Tc |
| FRT-vioA-up_rev | aggaacttcaagatccccaattcg CTGCTGCATGTCGAAAATG | One step cloning of *vioA*-up into pEX18Tc |
| FRT-vioA-dn_for | tcagagcgcttttgaagctaattcg CGTCCATGTGCACAAGTAC | One step cloning of *vioA*-dn into pEX18Tc |
| Gib-vioA-dn_rev | tacgaattcgagctcggtacccggg GCTCGCCATTGATCGAAAC | One step cloning of *vioA*-dn into pEX18Tc |
| PCR-frsA-KO_for | GTAATGTCAAAGGCTTGG | Mutant verification of *C. vaccinii* Δ*frsA* by PCR / Sequencing |
| PCR-frsA-KO_rev | ATTGAATTGCTGACACCG | Mutant verification of *C. vaccinii* Δ*frsA* by PCR / Sequencing |
| PCR-vioA-KO_for | AGCTCTACCTGTGGCAG | Mutant verification of *C. vaccinii* Δ*vioA* by PCR / Sequencing |
| PCR-vioA-KO_rev | TCCCAGGAGAAATGGTTG | Mutant verification of *C. vaccinii* Δ*vioA* by PCR / Sequencing |
| Gap1_for | TCGAGATGATGAAGGCTG | PCR-based closure of gap 1/ Sequencing |
| Gap1_rev | GGCATCAACACTTGATAAG | PCR-based closure of gap 1/ Sequencing |
| Gap2_for | TCCACCTCGATTTGTACG | PCR-based closure of gap 2 / Sequencing |
| Gap2_rev | CATGATCAACTCCTGACAG | PCR-based closure of gap 2 / Sequencing |
| Gap3_for | ATCTGGAACACCCGAATC | PCR-based closure of gap 3 / Sequencing |
| Gap3_rev | CAACTGGGCAACATAGCTG | PCR-based closure of gap 3 / Sequencing |
| Gap4_for | CGACTTGTTCTTCAATCTG | PCR-based closure of gap 4 / Sequencing |
| Gap4_rev | TCGAAACGATACAGGAACC | PCR-based closure of gap 4 / Sequencing |
| Gap5_for | CCCAATCATCTGGTATTGC | PCR-based closure of gap 5 / Sequencing |
| Gap5_rev | CCCAATCATCTGGTATTGC | PCR-based closure of gap 5 / Sequencing |
| Gap1_rev2 | CATCCAGCACGTACAGC | Sequencing / Primer walking |
| Gap1_rev3 | AGGCTTTGCAGATGGCG | Sequencing / Primer walking |
| Gap2_for2 | CCTATGTGATCTACACCTC | Sequencing / Primer walking |
| Gap3_for2 | TGCTGGAAATCGGCGTC | Sequencing / Primer walking |
| Gap4_for2 | GGGTGACGCTGAATACG | Sequencing / Primer walking |
| Gap4_for3 | AATACCCAGACCTACGTG | Sequencing / Primer walking |
| Gap4_rev2 | CGAAGAAGCTGTCGTCC | Sequencing / Primer walking |
| Gap4_rev3 | CCAGCAGCAGTTCCTTC | Sequencing / Primer walking |
| Cv_frsA-His6-N_for_BamHI | gat**ggatcc**ATGAAAAACAGTGAATCGC | In vitro studies |
| Cv_frsA-His6-N_rev_HindIII | tat**aagctt**TTATTGCTTGACAGCGGTGAC | In vitro studies |
| Cv-frsA-PCP-His6-N_rev_HindIII | tat**aagctt**tcaGCTGTCGCCGCCTTCGGC | In vitro studies |
| Cv-frsA-A-His6-N_for_BamHI | tat**ggatcc**CCGTCGCAGCCGGTGTCC | In vitro studies |
| Cv-frsA-TE-His6-N_for_BamHI | tat**ggatcc**GCCGAAGGCGGCGACAGC | In vitro studies |
| Cv_frsB_for_NdeI | gcg**catATG**AGCAATCCCTTTGATGAT | In vitro studies |
| Cv_frsB_rev_PacI_pCDF | gcg**ttaattaa**ttaTTTATCATCGCACTCCAT | In vitro studies |
| Cv_frsD-His6-N_for_HindIII | cac**aagctt**tgATGGAAATATGGCTGGCG | In vitro studies |
| Cv_frsD-His6-N_rev_XhoI | tat**ctcgag**TCAACTCCTGACAGCGTG | In vitro studies |
| Cv_frsH-His6-N_for_BamHI | gat**ggatcc**ATGACCGTATCCGATAAC | In vitro studies |
| Cv_frsH-His6-N_rev_XhoI | gata**ctcgag**tTACAGCAGCATGGTTTG | In vitro studies |

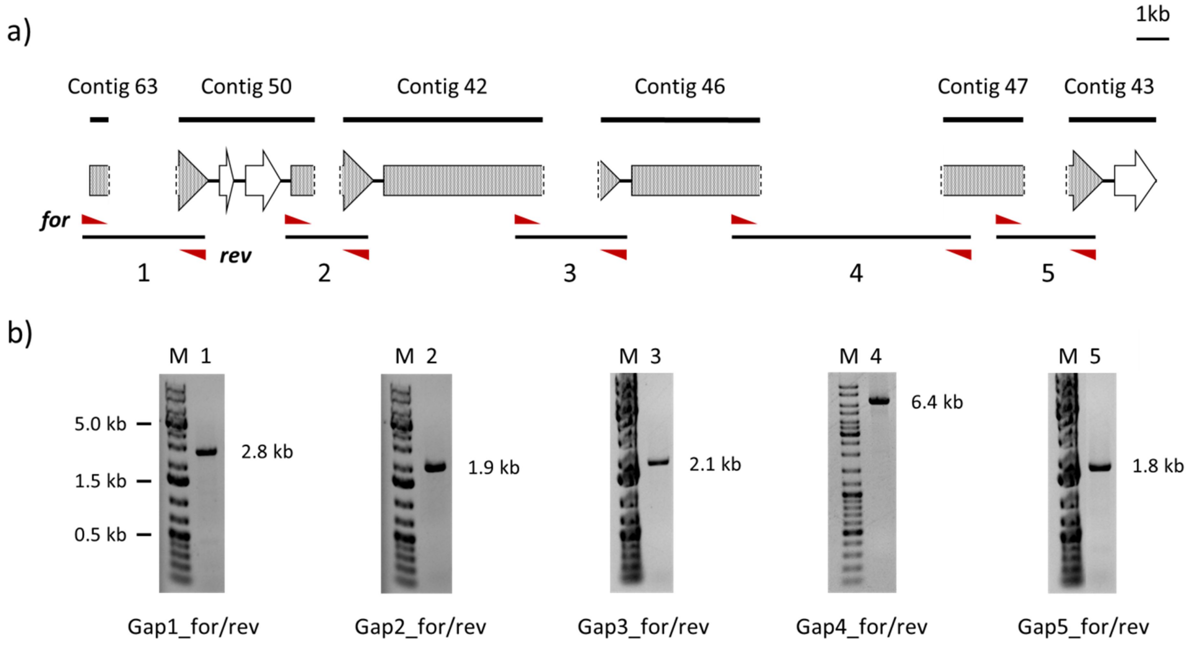


**Supplementary Figure 4: Gap closure of the *C. vaccinii frs* BGC.** a) PCR-based approach for closing the five potential gaps (1-5) within the gene cluster. Organization of the six contigs with *frs* identity shown here is based on the *frs* gene cluster from "*Ca.* B. crenata". Two additional contigs with *frs* identity (not shown) couldn't be assigned to a specific position. Binding sites of the respective forward (for) and reverse (rev) primers for each gap are indicated (red triangles). b) Q5-PCRs with the five primer pairs designed for gap closure (Gap1_for/rev to Gap5_for/rev). These experiments were independently repeated at least two times with similar results.

**Supplementary Table 2: Annotation of *C. vaccinii* Frs proteins and closest homologues in the NCBI database** (apart from "*Ca.* B. crenata" Frs proteins), revealed by BLAST searches.

| Protein | Functional annotation | Closest homologue (NCBI Accession number) | Similarity/Identity (%/%) |
|---|---|---|---|
| FrsA | Nonribosomal Peptide Synthetase (C-A-T-TE) | WP_048411362. Non-ribosomal peptide synthase [Chromobacterium sp. LK1] | 57/41 |
| FrsB | MbtH-like protein | MBC7269998.1 MbtH family protein [Streptomyces sp.] | 88/69 |
| FrsC | Phenylpyruvate reductase | NCT40463. malate dehydrogenase [Alpha-proteobacteria bacterium] | 65/53 |
| FrsD | Nonribosomal Peptide Synthetase (C-A-T) | WP_184971800.1 amino acid adenylation domain-containing protein [Streptomyces echinatus] | 59/46 |
| FrsE | Nonribosomal Peptide Synthetase (C-A-T-E-C-A-MT-T) | WP_162791553.1 non-ribosomal peptide synthase/polyketide synthase [Dyella sp. L4-6] | 63/52 |
| FrsF | Nonribosomal Peptide Synthetase (C-A-T-C-A-MT-T) | WP_073766514.1 non-ribosomal peptide synthetase [Streptomyces sp. CB02923] | 63/50 |
| FrsG | Nonribosomal Peptide Synthetase (C-A-T-C-A-MT-MT-T-TE) | WP_162791553.1 non-ribosomal peptide synthase/polyketide synthase [Dyella sp. L4-6] | 68/55 |
| FrsH | Leucine-ß-hydroxylase | WP_168647361.1 MBL fold metallo-hydrolase [Dyella sp. SG609] | 76/65 |

**Supplementary Table 3: Comparison of genes and encoded proteins in the *frs* BGCs**. Identities were calculated by using the EMBOSS needle alignment tool (EMBL-EBI).[2]

| gene | *C. vaccinii* (nt) | "*Ca.* B crenata" (nt) | Identity nt (%) | *C. vaccinii* (aa) | "*Ca.* B crenata" (aa) | Identity aa (%) |
|---|---|---|---|---|---|---|
| *frsA* | 3819 | 3768 | 70 | 1272 | 1255 | 71 |
| *frsB* | 219 | 219 | 72 | 72 | 72 | 75 |
| *frsC* | 987 | 987 | 68 | 328 | 328 | 72 |
| *frsD* | 3081 | 3078 | 70 | 1026 | 1025 | 70 |
| *frsE* | 9051 | 9048 | 70 | 3016 | 3015 | 71 |
| *frsF* | 7557 | 7560 | 73 | 2518 | 2519 | 75 |
| *frsG* | 9408 | 9411 | 72 | 3135 | 3136 | 73 |
| *frsH* | 1596 | 1599 | 77 | 531 | 532 | 85 |

```
frsA       1                                                   atgaaaaacagtgaatcgccaatccatcattttcaggcatcttcagc-ac     49
                                                               |||.|||      .||.|               |.|||       || ||
frsD       1                                                   atggaaa------tatgg---------------ctggc-------gcaac     22

frsA      50 agctggatgtatggatttctcaggaagtttcaccgaatctgcccaacaatattgccgagtatctgaatctcgccggctcgttggatgctggattgtttct    149
             |||| ||||           |.|||   |||.||||||         ||||||||||||||||||.||||..||||..||||||||.|.|..||||||.|
frsD      23 agct-gatg-----------ccgga---ttcgccgaat---------aatattgccgagtatctgcatctttccggtccgttggatccagatttgttttt     98

frsA     150 gcaggctttaagccaggtcgccagtgagagcgcggagc--tgcaatacaactt---ccgtcacgatggtc-tccagttgaccaagtttcgtcgagatgat    243
             ..|..|.||..|.||||||||.||.|||| |||.| ||  ||||...|||.||   |..|.|.|||||.| .||  |||.||  |.||.|.||.|...||
frsD      99 caaaaccttgcggcaggtcgcgagcgaga-cgccg-gcattgcaggtcaatttttccattgaggatggacggcc--ttgccc--ggttagccgcgtccat    192

frsA     244 gaaggctgggag-ccggacttcatcgatgtatcgacgcacggcgagccggaacacgcagccctgcgcgccatgcgggagcgggtggagaaacccttcgat    342
             ||.|.|| |||| ||.||.|||||||||||||||||||||||||||||||||||||||||||||||||||||||||||||||||||||||||||||||||
frsD     193 gaggact-ggagtcctgatttcatcgatgtatcgacgcacggcgagccggaacacgcagccctgcgcgccatgcgggagcgggtggagaaacccttcgat    291

frsA     343 ctggcgcgggacgcgttgtttcgctggaccttgatccgcctggccgacgagcgccacatcttctgccatgtgtatcaccacatcgcgatggatggggccg    442
             ||||||||||||||||||||||||||||||||||||||||||||||||||||||||||||||||||||||||||||||||||||||||||||..||||
frsD     292 ctggcgcgggacgcgttgtttcgctggaccttgatccgcctggccgacgagcgccacatcttctgccatgtgtatcaccacatcgcgatggatgtcgccg    391

frsA     443 gctatgtgatgctgctgcagcgcatagccgaggtttacggcgcgctgcgggaaggccagccggcaccggcctgcggtttcgccgatgcggatgccatcgt    542
             ||||||||||||||||||||||||||||||||||||||||||||||||||||||||||||||||||||||||||||||||||||||||||||||||||||
frsD     392 gctatgtgatgctgctgcagcgcatagccgaggtttacggcgcgctgcgggaaggccagccggcaccggcctgcggtttcgccgatgcggatgccatcgt    491

frsA     543 ccgcgaggaagagcgctaccgccagtcggagcagttcgcggtcgaccgggcattctggcaagcgcgctcggccgagctggcgacggcggagccgccgctg    642
             ||||||||||||||||||||||||||||||||||||||||||||||||||||||||||||||||||||||||||||||||||||||||||||||||||||
frsD     492 ccgcgaggaagagcgctaccgccagtcggagcagttcgcggtcgaccgggcattctggcaagcgcgctcggccgagctggcgacggcggagccgccgctg    591

frsA     643 ccggcggccgatggcccgttcctggcgttcgcccagacggcggtgattccggaagacgcctgcggcgggctgcggatgacggccgagcggctgggcgtct    742
             ||||||||||||||||||||||||||||||||||||||||||||||||||||||||||||||||||||||||||||||||||||||||||||||||||||
frsD     592 ccggcggccgatggcccgttcctggcgttcgcccagacggcggtgattccggaagacgcctgcggcgggctgcggatgacggccgagcggctgggcgtct    691

frsA     743 cccagtcccgtttgctgacagcagccatcgtcgcttatttccatcgctggggcggccagcaagagatcttgttccggctggcggtatcggcgcgcagcga    842
             ||||||||||||||||||||||.|||||||||||||||||||||||||||||||||||||||||||||||||||||||||||||||||||||||||||||
frsD     692 cccagtcccgtttgctgacagcggccatcgtcgcttatttccatcgctggggcggccagcaagagatcttgttccggctggcggtatcggcgcgcagcga    791

frsA     843 tgcgacgcgacacgcgcccggccacctggcgcatgcgttgccgctgctggccagcctgccgccgcgcgccagtctggccgacatcgcgcgacagctggac    942
             ||||||||||||||||||||||||||||||||||||||||||||||||||||||||||||||||||||||||||||||||||||||||||||||||||||
frsD     792 tgcgacgcgacacgcgcccggccacctggcgcatgcgttgccgctgctggccagcctgccgccgcgcgccagtctggccgacatcgcgcgacagctggac    891

frsA     943 ggcgaggtggagcggatgcgtccgcatacccgctatcgggctgaggacatcgtgcgcgaccaggccggtgccggtttggggcgcggggcgcaggggcctg   1042
             ||||||||||||||||||||||||||||||||||||||||||||||||||||||||||||||||||||||||||||||||||||||||||||||||||||
frsD     892 ggcgaggtggagcggatgcgtccgcatacccgctatcgggctgaggacatcgtgcgcgaccaggccggtgccggtttggggcgcggggcgcaggggcctg    991

frsA    1043 tgatcaacctcatgccttttgcttaccgcttcgagtttggcgcctgtcgcgtggagtccgcccatcagctgaccgtcggcgtgctggacacgctggaagt   1142
             ||||||||||||||||||||||||||||||||||||||||||||||||||||||||||||||||||||||||||||||||||||||||||||||||||||
frsD     992 tgatcaacctcatgccttttgcttaccgcttcgagtttggcgcctgtcgcgtggagtccgcccatcagctgaccgtcggcgtgctggacacgctggaagt   1091

frsA    1143 ggcggtgcacgaccgcaagaacggtgacggcctccacctcgatttgtacgcatccgagcgcggctgcccgcccgaaccgctgcggcggcatgccctgcgg   1242
             ||||||||||||||||||||||||||||||||||||||||||||||||||||||||||||||||||||||||||||||||||||||||||||||||||||
frsD    1092 ggcggtgcacgaccgcaagaacggtgacggcctccacctcgatttgtacgcatccgagcgcggctgcccgcccgaaccgctgcggcggcatgccctgcgg   1191
```

```
                                  |A domain start
frsA  1243 ctggcccggttcatcgtcgaggcggcggcggagccgtcgcagccggtgtccgacatcgagctgctggacgaggccgagcgccggcaactgctggtcgact  1342
           ||||||||||||||||||||||||||||||||||||||||||||||||||||||||||||||||||||||||||||||||||||||||||||||||||||
frsD  1192 ctggcccggttcatcgtcgaggcggcggcggagccgtcgcagccggtgtccgacatcgagctgctggacgaggccgagcgccggcaactgctggtcgact  1291
                                   ||||||||||||||||||||||||||||||||||||||||||||||||||||||||||||||||||||||||||||
frsG  1270                         gcggcggagccgtcgcagccggtgtccgacatcgagctgctggacgaggccgagcgccggcaactgctggtcgact  1345

frsA  1343 ggaaccgcaccggaccggaccacggccaggccaccttcccgcaactgttcgaaacccaggcggccctcaccccgcacgccgtcgcgctggaaagcccgga  1442
           ||||||||||||||||||||||||||||||||||||||||||||||||||||||||||||||||||||||||||||||||||||||||||||||||||||
frsD  1292 ggaaccgcaccggaccggaccacggccaggccaccttcccgcaactgttcgaaacccaggcggccctcaccccgcacgccgtcgcgctggaaagcccgga  1391
           ||||||||||||||||||||||||||||||||||||||||||||||||||||||||||||||||||||||||||||||||||||||||||||||||||||
frsG  1346 ggaaccgcaccggaccggaccacggccaggccaccttcccgcaactgttcgaaacccaggcggccctcaccccgcacgccgtcgcgctggaaagcccgga  1445

frsA  1443 cgcccggctcagctatgccgaactggacgcccgcgccaaccggctggcgcgccatctgcaaagcctgggcgtcggcgccgacgtgctggtcggcatctgc  1542
           ||||||||||||||||||||||||||||||||||||||||||||||||||||||||||||||||||||||||||||||||||||||||||||||||||||
frsD  1392 cgcccggctcagctatgccgaactggacgcccgcgccaaccggctggcgcgccatctgcaaagcctgggcgtcggcgccgacgtgctggtcggcatctgc  1491
           ||||||||||||||||||||||||||||||||||||||||||||||||||||||||||||||||||||||||||||||||||||||||||||||||||||
frsG  1446 cgcccggctcagctatgccgaactggacgcccgcgccaaccggctggcgcgccatctgcaaagcctgggcgtcggcgccgacgtgctggtcggcatctgc  1545

frsA  1543 ctggagcgctcgatcgacatggtggtcgcggtgctgggcgcgctgaagtccggcgccgcctatctgccgctgtcgccggagtacccgacggaacggctgg  1642
           ||||||||||||||||||||||||||||||||.|||||||.|||||||||||||||||||||||||||||||||||||||||||||||||||||||||||
frsD  1492 ctggagcgctcgatcgacatggtggtcgcggtactgggcgtgctgaagtccggcgccgcctatctgccgctgtcgccggagtacccgacggaacggctgg  1591
           ||||||||||||||||||||||||||||||||.|||||||.|||||||||||||||||||||||||||||||||||||||||||||||||||||||||||
frsG  1546 ctggagcgctcgatcgacatggtggtcgcggtgctgggcgcgctgaagtccggcgccgcctatctgccgctgtcgccggagtacccgacggaacggctgg  1645

frsA  1643 cctacatgctgggcgactcgatggcccccgtgctgctgaccgactcggcacaagtcgagcggctgccgtcgtattggggccgggtagtcgaactggaccg  1742
           ||||||||||||||||||||||||||||||||||||||||||||||||||||||||||||||||||||||||||||||||||||||||||||||||||||
frsD  1592 cctacatgctgggcgactcgatggcccccgtgctgctgaccgactcggcacaagtcgagcggctgccgtcgtattggggccgggtagtcgaactggaccg  1691
           ||||||||||||||||||||||||||||||||||||||||||||||||||||||||||||||||||||||||||||||||||||||||||||||||||||
frsG  1646 cctacatgctgggcgactcgatggcccccgtgctgctgaccgactcggcacaagtcgagcggctgccgtcgtattggggccgggtagtcgaactggaccg  1745

frsA  1743 gctcgacctggacgctctgccggacagcgcgccggaacgggcgctgcgcgccgagcacctggcctatgtgatctacacctccggctccaccggccaaccg  1842
           ||||||||||||||||||||||||||||||||||||||||||||||||||||||||||||||||||||||||||||||||||||||||||||||||||||
frsD  1692 gctcgacctggacgctctgccggacagcgcgccggaacgggcgctgcgcgccgagcacctggcctatgtgatctacacctccggctccaccggccaaccg  1791
           ||||||||||||||||||||||||||||||||||||||||||||||||||||||||||||||||||||||||||||||||||||||||||||||||||||
frsG  1746 gctcgacctggacgctctgccggacagcgcgccggaacgggcgctgcgcgccgagcacctggcctatgtgatctacacctccggctccaccggccaaccg  1845

frsA  1843 aagggcgtggcggtcagccacgccggcctggccggcctggccggcagccagacagagcggttcgcgctgcaaggcccgacgcgggtgctgcaattcgcct  1942
           ||||||||||||||||||||||||||||||||||||||||||||||||||||||||||||||||||||||||||||||||||||||||||||||||||||
frsD  1792 aagggcgtggcggtcagccacgccggcctggccggcctggccggcagccagacagagcggttcgcgctgcaaggcccgacgcgggtgctgcaattcgcct  1891
           ||||||||||||||||||||||||||||||||||||||||||||||||||||||||||||||||||||||||||||||||||||||||||||||||||||
frsG  1846 aagggcgtggcggtcagccacgccggcctggccggcctggccggcagccagacagagcggttcgcgctgcaaggcccgacgcgggtgctgcaattcgcct  1945

frsA  1943 cgctgagtttcgacgcggcggtgatggaaatgctgatggccttctgcagcggcggccggctggtgctgccggcggcggggccgctgctgggcgaacagct  2042
           ||||||||||||||||||||||||||||||||||||||||||||||||||||||||||||||||||||||||||||||||||||||||||||||||||||
frsD  1892 cgctgagtttcgacgcggcggtgatggaaatgctgatggccttctgcagcggcggccggctggtgctgccggcggcggggccgctgctgggcgaacagct  1991
           ||||||||||||||||||||||||||||||||||||||||||||||||||||||||||||||||||||||||||||||||||||||||||||||||||||
frsG  1946 cgctgagtttcgacgcggcggtgatggaaatgctgatggccttctgcagcggcggccggctggtgctgccggcggcggggccgctgctgggcgaacagct  2045

frsA  2043 gctggacacgctgaaccgccatgaaattagccacgcgctgatctcgccgtcggcgctgagcaccgcggacgcggcgttggcgccggtcctgcggacgctg  2142
           |||||||||||||||||||||||||||.||||||||||||||||||||||||||||||||||||||||||||||||||||||||||||||||||||||||
frsD  1992 gctggacacgctgaaccgccatgaaatcagccacgcgctgatctcgccgtcggcgctgagcaccgcggacgcggcgttggcgccggtcctgcggacgctg  2091
           |||||||||||||||||||||||||||.||||||||||||||||||||||||||||||||||||||||||||||||||||||||||||||||||||||||
frsG  2046 gctggacacgctgaaccgccatgaaattagccacgcgctgatctcgccgtcggcgctgagcaccgcggacgcggcgttggcgccggtcctgcggacgctg  2145

frsA  2143 gtggtgggcggggaagcctgcccggcgcgacggtggcggcctggtcggcgggacggcggatggtgaacgcctacggtccgaccgaggcgacggcctgcg  2242
           ||||||||||||||||||||||||||||||||||||||||||||||||||||||||||||||||||||||||||||||||||||||||||||||||||||
frsD  2092 gtggtgggcggggaagcctgcccggcgcgacggtggcggcctggtcggcgggacggcggatggtgaacgcctacggtccgaccgaggcgacggcctgcg  2191
           ||||||||||||||||||||||||||||||||||||||||||||||||||||||||||||||||||||||||||||||||||||||||||||||||||||
frsG  2146 gtggtgggcggggaagcctgcccggcgcgacggtggcggcctggtcggcgggacggcggatggtgaacgcctacggtccgaccgaggcgacggcctgcg  2245

frsA  2243 tgacgatgagcgagccgctgtccggcgacggcgcgccgaagctgggccgtccgacgcacaacgcgcggctgtacgtgctggatggcgcgctgcaactggc  2342
           ||||||||||||||||||||||||||||||||||||||||||||||||||||||||||||||||||||||||||||||||||||||||||||||||||||
frsD  2192 tgacgatgagcgagccgctgtccggcgacggcgcgccgaagctgggccgtccgacgcacaacgcgcggctgtacgtgctggatggcgcgctgcaactggc  2291
           ||||||||||||||||||||||||||||||||||||||||||||||||||||||||||||||||||||||||||||||||||||||||||||||||||||
frsG  2246 tgacgatgagcgagccgctgtccggcgacggcgcgccgaagctgggccgtccgacgcacaacgcgcggctgtacgtgctggatggcgcgctgcaactggc  2345

frsA  2343 gccggtgggggtggcgggcgagctgtacatcgcgggggccgggctggcgcgcggctatctgaaccggccggggctgacggcggagcgcttcgtggcgaat  2442
           ||||||||||||||||||||||||||||||||||||||||||||||||||||||||||||||||||||||||||||||||||||||||||||||||||||
frsD  2292 gccggtgggggtggcgggcgagctgtacatcgcgggggccgggctggcgcgcggctatctgaaccggccggggctgacggcggagcgcttcgtggcgaat  2391
           ||||||||||||||||||||||||||||||||||||||||||||||||||||||||||||||||||||||||||||||||||||||||||||||||||||
frsG  2346 gccggtgggggtggcgggcgagctgtacatcgcgggggccgggctggcgcgcggctatctgaaccggccggggctgacggcggagcgcttcgtggcgaat  2445

frsA  2443 ccgtacggagagggtgagcggctgtaccgcagcggcgacctggcgcggtggacggaagaaggcgagctggaatacctggggcgcagcgaccagcaggtga  2542
           ||||||||||||||||||||||||||||||||||||||||||||||||||||||||||||||||||||||||||||||||||||||||||||||||||||
frsD  2392 ccgtacggagagggtgagcggctgtaccgcagcggcgacctggcgcggtggacggaagaaggcgagctggaatacctggggcgcagcgaccagcaggtga  2491
           ||||||||||||||||||||||||||||||||||||||||||||||||||||||||||||||||||||||||||||||||||||||||||||||||||||
frsG  2446 ccgtacggagagggtgagcggctgtaccgcagcggcgacctggcgcggtggacggaagaaggcgagctggaatacctggggcgcagcgaccagcaggtga  2545

frsA  2543 aggtgcggggtttccgtatcgagccgggcgagatcgaagcggtgctgaaccggcatccgcaagtgagccagtcggtggtggtggcgcggcagagccaggg  2642
           ||||||||||||||||||||||||||||||||||||||||||||||||||||||||||||||||||||||||||||||||||||||||||||||||||||
frsD  2492 aggtgcggggtttccgtatcgagccgggcgagatcgaagcggtgctgaaccggcatccgcaagtgagccagtcggtggtggtggcgcggcagagccaggg  2591
           ||||||||||||||||||||||||||||||||||||||||||||||||||||||||||||||||||||||||||||||||||||||||||||||||||||
frsG  2546 aggtgcggggtttccgtatcgagccgggcgagatcgaagcggtgctgaaccggcatccgcaagtgagccagtcggtggtggtggcgcggcagagccaggg  2645

frsA  2643 cggcgacagccagttggtggcgtacgtggcggccgtcggcggggtggaggggtcggagctgcggcgcctggcggcggggcagctgccggagcacatggtg  2742
           ||||||||||||||||||||||||||||||||||||||||||||||||||||||||||||||||||||||||||||||||||||||||||||||||||||
frsD  2592 cggcgacagccagttggtggcgtacgtggcggccgtcggcggggtggaggggtcggagctgcggcgcctggcggcggggcagctgccggagcacatggtg  2691
           ||||||||||||||||||||||||||||||||||||||||||||||||||||||||||||||||||||||||||||||||||||||||||||||||||||
frsG  2646 cggcgacagccagttggtggcgtacgtggcggccgtcggcggggtggaggggtcggagctgcggcgcctggcggcggggcagctgccggagcacatggtg  2745
```

```
frsA  2743 ccggcggcggtggtggtgctggaatcgctgccgcagttgccgaacgggaagctggaccgcaagtcgctgccggcgccggagtttggcggctcgcattatc   2842
           ||||||||||||||||||||||||||||||||||||||||||||||||||||||||||||||||||||||||||||||||||||||||||||||||||||
frsD  2692 ccggcggcggtggtggtgctggaatcgctgccgcagttgccgaacgggaagctggaccgcaagtcgctgccggcgccggagtttggcggctcgcattatc   2791
           ||||||||||||||||||||||||||||||||||||||||||||||||||||||||||||||||||||||||||||||||||||||||||||||||||||
frsG  2746 ccggcggcggtggtggtgctggaatcgctgccgcagttgccgaacgggaagctggaccgcaagtcgctgccggcgccggagtttggcggctcgcattatc   2845

frsA  2843 agcggccgcgcaacgcgcaggaggaaatgctgtgcgggctgttcgcggaagtgctggacatggagaaggttggcaggggagacagcttcttcgatctggg   2942
           ||||||||||||||||||||||||||||||||||||||||||||||||||||||||||..|||.||..||.||.|..|..|||||.||||||||||||||
frsD  2792 agcggccgcgcaacgcgcaggaggaaatgctgtgcgggctgttcgcggaagtgctggaagtggggagcgtcgggatagacgacagtttcttcgatctggg   2891
           ||||||||||||||||||||||||||||||||||||||||||||||||||||||||||..|||.||..||.||.|..|..|||||.||||||||||||||
frsG  2846 agcggccgcgcaacgcgcaggaggaaatgctgtgcgggctgttcgcggaagtgctggaagtggggagcgtcgggatagacgacagtttcttcgatctggg   2945
                A domain end |

frsA  2943 cgggcactcgttgctggcgacgcggctgatccgccgcatccgcgaaaccttggatgtggagctgtcgatccgcgatctgttcgaggct-ccctgcgtcac   3041
           |||||||||||||||||||||||||||||||.||||||||||.|...|||||.|||||||||||.||||||||.|.|||||||| .|| ||||.||||.|
frsD  2892 cgggcactcgttgctggcgacgcggctgatcagccgcatccgggcggccttgaatgtggagctgccgatccgccagctgttcga-cctgccctccgtcgc   2990
           |||||||||||||||||||||||||||||||.||||||||||.|...|||||.|||||||||||.|||||||..|.|||||||| .|||||
frsG  2946 cgggcactcgttgctggcgacgcggctgatcagccgcatccgggcggccttgaatgtggagctgtcgatccgggagctgttcga-catgcc           3035

frsA  3042 ggaactg----------tcccggcatatc-------------------------gccgaaggcggcgacagcaaga-gccctttatcaagtgttgatgcct   3105
           .||.|||          ||||...|||.|                         |||| .|.||.||.||||||.| ||   |.|||.|.|||||
frsD  2991 agagctgcttgaggttctccccaataccaaggcgctgcccggccggccctgcagccg-cgccgccgccagcaacacgc---tgtcaggagttga-----   3081

frsA  3106 attcgggccactggcggccgccatcccttgttctgcattcatcccgagggcgggttgggttggagctatatcgggctggctttgcatctggaccatgagc   3205

frsD  3082 ----------------------------------------------------------------------------------------------------   3081

frsA  3206 aaccgatctacaccctgcaagcccggggcctggacggcatgtcggagttggctccgtcgattccggatatggctgccgactatatcgagcaaatccgcag   3305

frsD  3082 ----------------------------------------------------------------------------------------------------   3081

frsA  3306 cattcagccgaatggcccctatcacttgctgggctggtcgctgggggggtgatcgctcaggaagtggcggtgcagttggagcgggtcggggaaaagaca   3405

frsD  3082 ----------------------------------------------------------------------------------------------------   3081

frsA  3406 gcgttgctggcaattctggatacgtttccgattgaaatcctgcatgaggcgatgtttggcaagcaagcctgcgcttatgaccttttttgcccgtgtggttc   3505

frsD  3082 ----------------------------------------------------------------------------------------------------   3081

frsA  3506 aggaaatgtatttgatgccgatcgaggaggcccgattgaagagcatgtatctgatcggtctcaaccatatgaagatcactgcggcctttttcctcctctca   3605

frsD  3082 ----------------------------------------------------------------------------------------------------   3081

frsA  3606 ttatggtggcgatttgctgctatttcgctccttgattccatatgccgaagacgcgctgatgccagaggcggatacatggcagccttatttgtctggccaa   3705

frsD  3082 ----------------------------------------------------------------------------------------------------   3081

frsA  3706 ttggaagttcatgacatcgagtgcacacatatggacatgatgcaaagagatgttttgaaaataattggtcctgttctcgagtcgaagttgtctgtcaccg   3805

frsD  3082 ----------------------------------------------------------------------------------------------------   3081

frsA  3806 ctgtcaagcaataa   3819

frsD  3082 --------------   3081
```

**Supplementary Figure 5: Emboss Needle Alignment of *frsA* and *frsD* and the A domain encoding sequence of *frsG* (bp 1270-3035) in *C. vaccinii.*** Identical base pairs are highlighted in yellow.

```
FrsA    1 MKNSESPIHHFQASSAQLDVWISQEVSPNLPNNIAEYLNLAGSLDAGLFLQALSQVASESAELQYNFRHDGLQLTKFRRDDEGWEPDFIDVSTHGEPEHA    100
                           :::|::|::.|:.||||||||:|:|.||..||.:.|.|||||:..||.||..:..:.....|..|.|.|||||||||||||||
FrsD    1 -----------------MEIWLAQQLMPDSPNNIAEYLHLSGPLDPDLFFKTLRQVASETPALQVNFSIEDGRPCPVSRVHEDWSPDFIDVSTHGEPEHA     83

FrsA  101 ALRAMRERVEKPFDLARDALFRWTLIRLADERHIFCHVYHHIAMDGAGYVMLLQRIAEVYGALREGQPAPACGFADADAIVREEERYRQSEQFAVDRAFW    200
          |||||||||||||||||||||||||||||||||||||||||||||.||||||||||||||||||||||||||||||||||||||||||||||||||||||
FrsD   84 ALRAMRERVEKPFDLARDALFRWTLIRLADERHIFCHVYHHIAMDVAGYVMLLQRIAEVYGALREGQPAPACGFADADAIVREEERYRQSEQFAVDRAFW    183

FrsA  201 QARSAELATAEPPLPAADGPFLAFAQTAVIPEDACGGLRMTAERLGVSQSRLLTAAIVAYFHRWGGQQEILFRLAVSARSDATRHAPGHLAHALPLLASL    300
          ||||||||||||||||||||||||||||||||||||||||||||||||||||||||||||||||||||||||||||||||||||||||||||||||||||
FrsD  184 QARSAELATAEPPLPAADGPFLAFAQTAVIPEDACGGLRMTAERLGVSQSRLLTAAIVAYFHRWGGQQEILFRLAVSARSDATRHAPGHLAHALPLLASL    283

FrsA  301 PPRASLADIARQLDGEVERMRPHTRYRAEDIVRDQAGAGLGRGAQGPVINLMPFAYRFEFGACRVESAHQLTVGVLDTLEVAVHDRKNGDGLHLDLYASE    400
          ||||||||||||||||||||||||||||||||||||||||||||||||||||||||||||||||||||||||||||||||||||||||||||||||||||
FrsD  284 PPRASLADIARQLDGEVERMRPHTRYRAEDIVRDQAGAGLGRGAQGPVINLMPFAYRFEFGACRVESAHQLTVGVLDTLEVAVHDRKNGDGLHLDLYASE    383

FrsA  401 RGCPPEPLRRHALRLARFIVEAAAEPSQPVSDIELLDEAERRQLLVDWNRTGPDHGQATFPQLFETQAALTPHAVALESPDARLSYAELDARANRLARHL    500
          ||||||||||||||||||||||||||||||||||||||||||||||||||||||||||||||||||||||||||||||||||||||||||||||||||||
FrsD  384 RGCPPEPLRRHALRLARFIVEAAAEPSQPVSDIELLDEAERRQLLVDWNRTGPDHGQATFPQLFETQAALTPHAVALESPDARLSYAELDARANRLARHL    483

FrsA  501 QSLGVGADVLVGICLERSIDMVVAVLGALKSGAAYLPLSPEYPTERLAYMLGDSMAPVLLTDSAQVERLPSYWGRVVELDRLDLDALPDSAPERALRAEH    600
          |||||||||||||||||||||||||||.||||||||||||||||||||||||||||||||||||||||||||||||||||||||||||||||||||||||
FrsD  484 QSLGVGADVLVGICLERSIDMVVAVLGVLKSGAAYLPLSPEYPTERLAYMLGDSMAPVLLTDSAQVERLPSYWGRVVELDRLDLDALPDSAPERALRAEH    583

FrsA  601 LAYVIYTSGSTGQPKGVAVSHAGLAGLAGSQTERFALQGPTRVLQFASLSFDAAVMEMLMAFCSGGRLVLPAAGPLLGEQLLDTLNRHEISHALISPSAL    700
          ||||||||||||||||||||||||||||||||||||||||||||||||||||||||||||||||||||||||||||||||||||||||||||||||||||
FrsD  584 LAYVIYTSGSTGQPKGVAVSHAGLAGLAGSQTERFALQGPTRVLQFASLSFDAAVMEMLMAFCSGGRLVLPAAGPLLGEQLLDTLNRHEISHALISPSAL    683

FrsA  701 STADAALAPVLRTLVVGGEACPGATVAAWSAGRRMVNAYGPTEATACVTMSEPLSGDGAPKLGRPTHNARLYVLDGALQLAPVGVAGELYIAGAGLARGY    800
          ||||||||||||||||||||||||||||||||||||||||||||||||||||||||||||||||||||||||||||||||||||||||||||||||||||
FrsD  684 STADAALAPVLRTLVVGGEACPGATVAAWSAGRRMVNAYGPTEATACVTMSEPLSGDGAPKLGRPTHNARLYVLDGALQLAPVGVAGELYIAGAGLARGY    783

FrsA  801 LNRPGLTAERFVANPYGEGERLYRSGDLARWTEEGELEYLGRSDQQVKVRGFRIEPGEIEAVLNRHPQVSQSVVVARQSQGGDSQLVAYVAAVGGVEGSE    900
          ||||||||||||||||||||||||||||||||||||||||||||||||||||||||||||||||||||||||||||||||||||||||||||||||||||
FrsD  784 LNRPGLTAERFVANPYGEGERLYRSGDLARWTEEGELEYLGRSDQQVKVRGFRIEPGEIEAVLNRHPQVSQSVVVARQSQGGDSQLVAYVAAVGGVEGSE    883

FrsA  901 LRRLAAGQLPEHMVPAAVVVLESLPQLPNGKLDRKSLPAPEFGGSHYQRPRNAQEEMLCGLFAEVLDMEKVGRGDSFFDLGGHSLLATRLIRRIRETLDV   1000
          ||||||||||||||||||||||||||||||||||||||||||||||||||||||||||||||||||::..||..|||||||||||||||||.|||..|:|
FrsD  884 LRRLAAGQLPEHMVPAAVVVLESLPQLPNGKLDRKSLPAPEFGGSHYQRPRNAQEEMLCGLFAEVLEVGSVGIDDSFFDLGGHSLLATRLISRIRAALNV    983

FrsA 1001 ELSIRDLFEAPCVTELSRHIAEGGDSKSPYQVLMP-------IRATGGRHPLFCIHPEGGLGWSYIGLALHLDHEQPIYTLQARGLDGMSELAPSIPDMA   1093
          ||.||.||:.|.|.||...:.:...:..|  .|.|       :|:.
FrsD  984 ELPIRQLFDLPSVAELLEVLPQYQGAARP--ALQPRRRQQHAVRS*-------------------------------------------------------   1027

FrsA 1094 ADYIEQIRSIQPNGPYHLLGWSLGGVIAQEVAVQLERVGEKTALLAILDTFPIEILHEAMFGKQACAYDLFARVVQEMYLMPIEEARLKSMYLIGLNHMK   1193

FrsD 1028 ----------------------------------------------------------------------------------------------------   1027

FrsA 1194 ITAAFSSSHYGGDLLLFRSLIPYAEDALMPEADTWQPYLSGQLEVHDIECTHMDMMQRDVLKIIGPVLESKLSVTAVKQ*   1273

FrsD 1028 --------------------------------------------------------------------------------   1027
```

**Supplementary Figure 6: Emboss Needle Alignment of FrsA and FrsD in *C. vaccinii.*** Identical amino acids are highlighted in yellow.

# Establishment of Knock-out System in *C. vaccinii* and Creation of Mutant Strains

## Construction of the *frsA* and *vioA* knock-out vectors

At first, a classical multiple cloning procedure (sequential cloning) was used to generate the *C. vaccinii* MWU205 Δ*frsA* mutant. Later, a more elaborate method, based on Gibson assembly (one step cloning) was employed to delete *vioA* (Supplementary Fig. 7). For both approaches, the upstream (up) and downstream (dn) DNA sequences of the respective genes (ca. 1.1 kb for *frsA* and ca. 0.4 kb for *vioA*) were amplified by Q5 PCR with suitable primers (Supplementary Table 1). In addition the 1.8 kb FRT cassette (Gem$^R$ Gfp$^+$) (FRT) from pPS858[3] was amplified by Q5 PCR. All PCR reactions were prepared in a 25 µL scale as described above with either 2–22 ng *C. vaccinii* MWU205 gDNA (*frsA* and *vioA* up- and downstream) or 0.5–4.5 ng pPS858 (FRT) as template. The fragments for sequential cloning were then cloned into pUC19[4] in a directional manner in the following order: FRT > *frsA*-up > *frsA*-dn. For this, standard restriction/ligation based cloning techniques were used. The insert of the resulting plasmid pUC19::Δ*frsA*, consisting of all three fragments, was then subcloned into pEX18Tc by using the flanking restriction sites for *Sac*I and *Sph*I. This yielded the final knock-out vector pEX18TC::Δ*frsA*. In contrast, the one step cloning approach followed the protocol of Gibson et al.[5] For this, a reaction mixture (20 µL scale) containing 4.5 nM of the PCR amplified inserts (FRT, *vioA*-up, *vioA*-dn), 1.375 nM *Bam*HI linearized pEX18Tc, 0.0054 U/µL T5 exonuclease (NEB), 0.034 U/µL Phusion polymerase (NEB), 5.4 U/µL *Taq* DNA ligase (NEB) and 1x ISO buffer (10 mM $MgCl_2$, 200 µM deoxynucleotide triphosphates, 10 mM DTT, 5% (w/v) PEG-8000, 1 mM NAD, 100 mM Tris-HCl pH 7.5) was set up. This mixture was then incubated at 50 °C for 1 h. 5 µL of this reaction mixture were then transformed into chemically competent *E. coli* NEB Turbo cells (NEB). Positive clones were initially identified by colony PCR. For this, screening reactions were performed in a 25 µL scale with 1x GoTaq reaction buffer with 1.5 mM $MgCl_2$, 4% DMSO, 200 µM dNTP, 100 nM of forward and reverse primer, DNA template (bacterial suspension in water) and GoTaq G2 DNA polymerase (0.025 U/µL, Promega). Plasmids from positive clones were then verified by restriction digest analysis as well as terminal-end Sanger sequencing. This yielded the final knock-out vector pEX18Tc::Δ*vioA*.

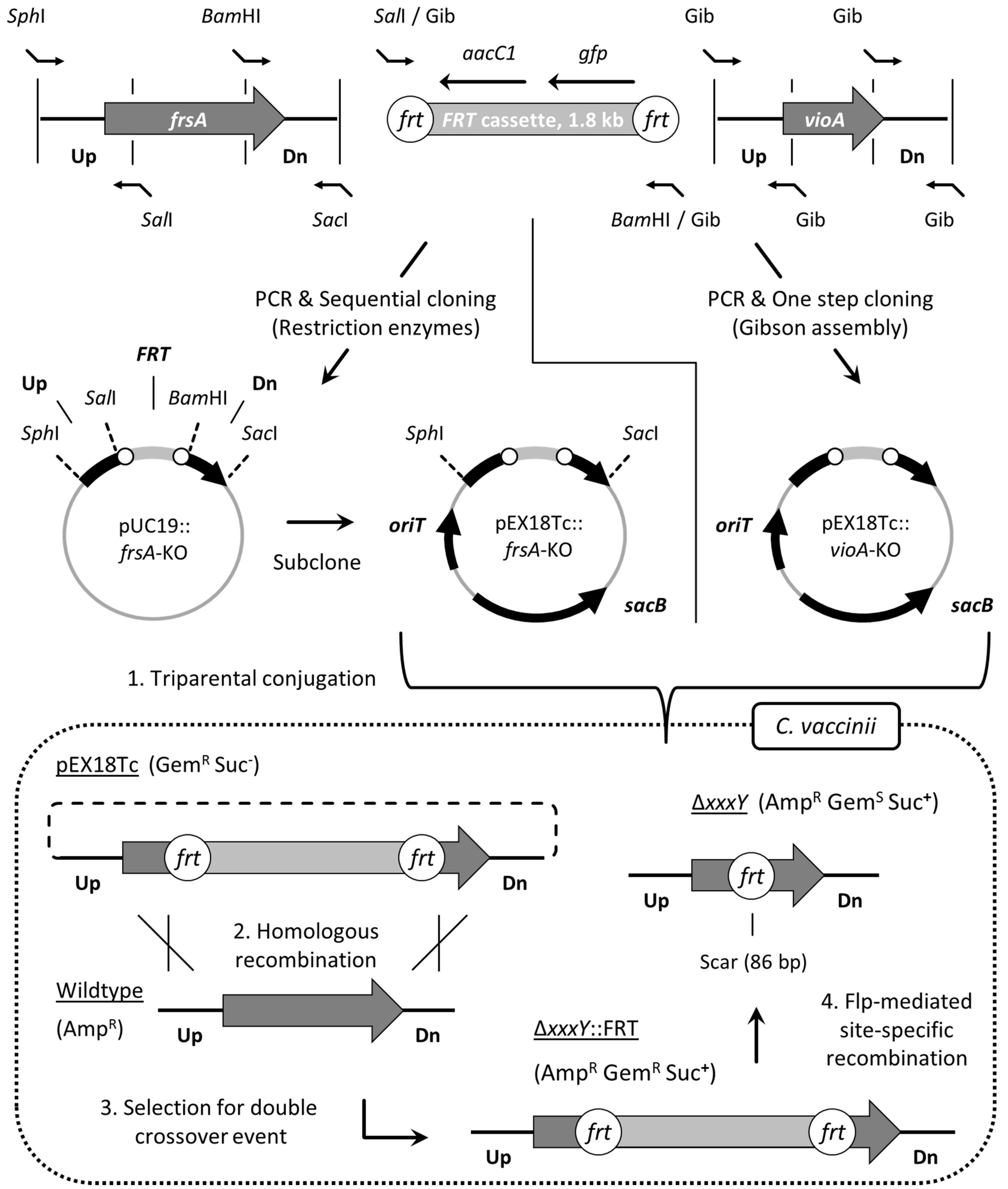


**Supplementary Figure 7: Schematic representation of the deletion strategy used for *frsA* and *vioA*.** This involves the construction of the knock-out vectors pEX18Tc::*frsA*-KO (sequential cloning) and pEX18Tc::*vioA*-KO (Gibson assembly) (upper half) and their use for generation of the *C. vaccinii* Δ*frsA* and Δ*vioA* deletion mutants (lower half). This involves 1. Transfer of the knock-out vectors by triparental conjugation, 2. Exchange of the targeted gene with the *FRT* cassette (*aacC1* and *gfp* flanked by two *frt* sites) by homologous recombination, 3. Selection for an exchange by double homologous recombination as well as loss of the knock-out vector and 4. Removal of the *FRT* cassette by Flp-mediated site-specific recombination between the two *frt* sites. Restriction enzyme cutting sites used for cloning as well as overhangs necessary for Gibson assembly (Gib) are indicated. For further details see respective sections in manuscript and Materials and Methods. (Δ*xxxY*::FRT = deleted gene with integrated FRT cassette; Δ*xxxY* = deleted gene with scar; $Amp^R$ = Ampicillin resistant; $Gem^R$ / $Gem^S$ = Gentamicin resistant / sensitive; $Suc^+$ / $Suc^-$ = Sucrose unsusceptible / susceptible).

## Preparation of knockout mutants

Transfer of pEX18Tc::Δ*frsA* or pEX18Tc::Δ*vioA* into *C. vaccinii* MWU205 occurred by triparental conjugation employing *E. coli* NEBTurbo harboring one of the knockout vectors and the conjugational helper strain *E. coli* ET12567 pUB307. All three strains were grown in 15 mL LB at 30 °C, 220 rpm to an OD600 of 0.4 - 0.6. Cells were then washed two times with 10 mL LB to remove any antibiotics and were finally resuspended in an appropriate amount of LB. The resulting suspensions were then mixed in a 3:1 ratio of donor strains and acceptor strain (600 µL *E. coli* NEBTurbo with pEX18Tc::Δ*frsA* or pEX18Tc::Δ*vioA*, 600 µL *E. coli* ET12567 pUB307, 200 µL *C. vaccinii* MWU205), centrifuged (2 min, 9,000 rpm, RT) and the pellet resuspended in a small part of the supernatant (ca. 100 µL). This mixture was applied to a LB agar plate without antibiotics in the form of a 'puddle'. After drying, the plate was incubated for 24 h at 30 °C. On the next day, the resulting cell layer was scraped off and resuspended in 1 mL LB without NaCl (NS-LB). The suspension was then plated on NS-LB agar containing Amp200, Gem30 and 15% sucrose (w/v) and the plates were incubated for 60–72 h at 25 °C. Resulting clones were screened for a successful double homologous recombination event by colony PCR as described above. Three positive clones were then further tested for an integration of the FRT cassette at the right genomic locus. For this, the respective regions were amplified by Q5 PCR with primers binding outside the sequences used for construction of the knockout vectors (Supplementary Table 1, Supplementary Fig. 8a). All PCR reactions were prepared in a 25 µL scale with 75 - 200 ng of *C. vaccinii* MWU205 mutant gDNA as template as described above. Resulting PCR products which showed the expected size difference (minus 1,897 bp for Δ*frsA*::FRT, plus 879 bp for Δ*vioA*::FRT) compared to the wild type (6,144 bp for *frsA*, 2,043 bp for *vioA*) (Supplementary Fig. 8b), were purified and applied to terminal-end Sanger sequencing along with the primers used for amplification. As pUB307 is a self-transferable helper plasmid, all correct clones were routinely tested for the loss of pUB307 by plating them on kanamycin and tetracycline. Only clones, who failed to grow on either antibiotic, were considered for further usage as this indicates a loss of the plasmid. Alternatively, cells were cured from pUB307 by growing them in LB supplemented with 5 % (w/v) SDS at 30 °C for 24–48 h and replating them on selective agar. To remove the FRT cassette from the genome of the *C. vaccinii* Δ*frsA*::FRT or Δ*vioA*::FRT deletion mutants, the genes *flp* and *sacB* from pFlp2[3] were introduced into the broad host vector pBMTL-2[6] as described by Wang et al.[7] The resulting vector pBMTL-2::*flp-sacB* was then transferred to the deletion mutants by electroporation following the protocol established for *Chromobacterium violaceum*.[8] After the transformation clones were grown overnight at 30 °C, which has been shown to be sufficient for FRT removal by Flp mediated site specific recombination.[3] Positive clones that lost the FRT cassette were identified by colony PCR and plated on NS-LB with Amp200 and 15% sucrose (w/v) to remove the pBMTL-2::*flp* plasmid. Loss of the plasmid was then confirmed by colony PCR as well as testing the clones for kanamycin susceptibility. To verify modification of the correct genomic locus, three clones were further investigated by Q5 PCR as described above. In this case, removal of the FRT cassette was indicated by the formation of a smaller

PCR product (loss of 1,725 bp) compared to the mutants with FRT cassette. The mutants verified in this manner were termed *C. vaccinii* MWU205 Δ*frsA* and *C. vaccinii* MWU205 Δ*vioA*, respectively. The double mutant *C. vaccinii* MWU205 Δ*frsA*/Δ*vioA* was constructed as described above by using *C. vaccinii* MWU205 Δ*frsA* as starting strain. Mutant strains were grown and extracted analogous to the wild type strain (see above).

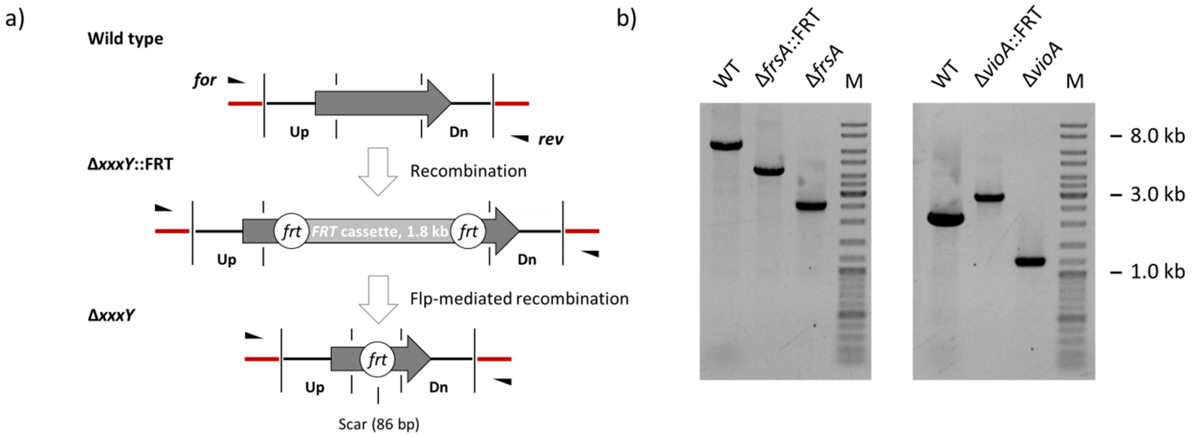


**Supplementary Figure 8: Verification of *C. vaccinii* deletion mutants.** a) PCR-based genotype verification applied during the construction of markerless *C. vaccinii* deletion mutants. All primers used for amplification (for/rev) bind to the regions outside the sequences used for knock-out vector construction (Up/Dn), which are shown here in red. This ensures a modification at the right locus. b) Q5-PCR of the *C. vaccinii* wild type as well as the different deletion mutants (Δ*frsA*::FRT, Δ*frsA*, Δ*vioA*::FRT, Δ*vioA*) with the respective verification primers (PCR-frsA_for/rev or PCR-vioA_for/rev). PCRs performed with the same primer pairs for the *C. vaccinii* Δ*frsA/*Δ*vioA* double mutant yielded identical results as for the single knock-out mutants. (ΔxxxY::FRT = deleted gene with integrated FRT cassette; ΔxxxY = deleted gene with scar). These experiments were independently repeated at least three times with similar results.

## Structure elucidation of FR-Core (2)

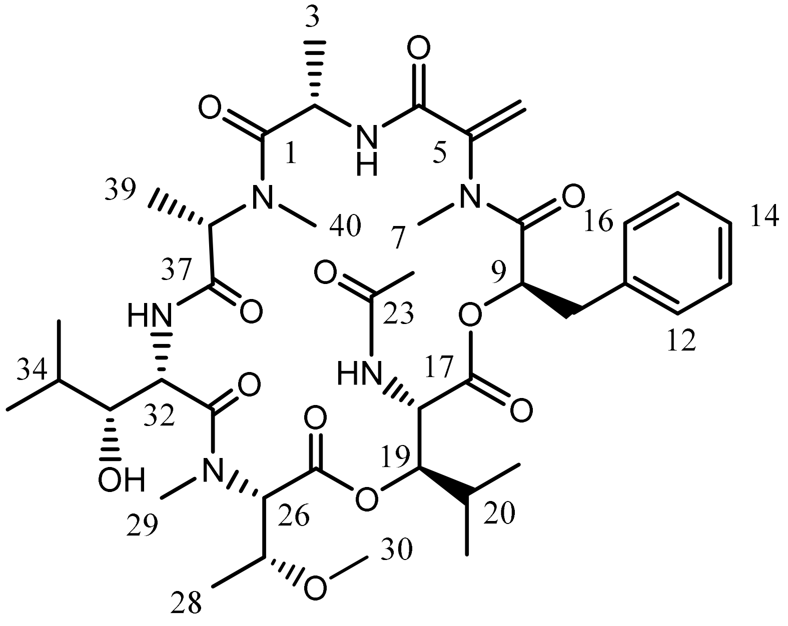


**Supplementary Figure 9: Chemical structure of FR-Core** (**2**). Carbon atoms are numbered.

**Supplementary Table 4:** $^{1}$H and $^{13}$C NMR spectroscopic data of **2** (see Supplementary Figure 9) in acetonitrile-*d*$_3$ ($^{1}$H: 600 MHz; $^{13}$C: 150 MHz).

| Residue[a] | No C/H | $\delta_C$, mult | $\delta_H$ ( *J* [Hz]) |
|---|---|---|---|
| Ala | 1 | 172.5, C | – |
| | 2 | 44.3, CH | 5.11 (dq, 9.1, 6.7) |
| | 2-NH | – | 7.12 (d, 9.1) |
| | 3 | 16.7, $CH_3$ | 1.19 (d, 6.7) |
| *N*-Me-Dha | 4 | 162.2, C | – |
| | 5 | 142.1, C | – |
| | 6a | 122.8, $CH_2$ | a 5.62 (br s) |
| | 6b | – | b 3.60 (br s) |
| | 7 | 36.6, $CH_3$ | 2.89 (s) |
| Pla | 8 | 168.5, C | – |
| | 9 | 71.6, CH | 5.56 (dd, 4.3, 10.5) |
| | 10a | 38.5, $CH_2$ | a 3.15 (dd, 4.3, 12.5) |
| | 10b | – | b 3.10 (dd, 10.5, 12.5) |
| | 11 | 136.3, C | – |
| | 12/16 | 130.5, CH | 7.24[b] |
| | 13/15 | 128.9, CH | 7.29[b] |
| | 14 | 127.5, CH | 7.27[b] |
| *N*-Ac-*β*-OH-Leu | 17 | 169.7, C | – |
| | 18 | 53.1, CH | 4.91 (dd, 2.2, 9.6) |
| | 18-NH | – | 6.88, (d, 9,6) |
| | 19 | 78.3, CH | 5.41 (br d, 10.0) |
| | 20 | 30.4, CH | 1.79 (m) |
| | 21 | 18.6, $CH_3$ | 0.83 (d, 6.8) |
| | 22 | 18.1, $CH_3$ | 0.87 (d, 6.8) |
| | 23 | 170.5, C | – |

| | | | |
|---|---|---|---|
| | 24 | 22.4, $CH_3$ | 2.02 (s) |
| *N,O*-$Me_2$-Thr | 25 | 168.1, C | – |
| | 26 | 67.9, CH | 3.55 (d, 9.8) |
| | 27 | 74.1, CH | 3.95 (dq, 9.8, 5.9 ) |
| | 28 | 17.8, $CH_3$ | 1.26 (d, 5.9) |
| | 29 | 40.0, $CH_3$ | 3.23 (s) |
| | 30 | 56.5, $CH_3$ | 3.27 (s) |
| *β*-OH-Leu | 31 | 170.5, C | – |
| | 32 | 50.3, CH | 4.95 (dd, 6.9, 10.4) |
| | 32-NH | | 6.94, (d, 10.4) |
| | 33 | 77.1, CH | 3.42 (br t, 5.8) |
| | 34 | 28.8, CH | 1.93 (m) |
| | 35 | 20.3, $CH_3$ | 1.02 (d, 6.7) |
| | 36 | 15.6, $CH_3$ | 0.93 (d, 6.7) |
| *N*-Me-Ala | 37 | 169.6, C | – |
| | 38 | 61.4, CH | 3.61 (q, 6.7) |
| | 39 | 12.3, $CH_3$ | 1.38 (d, 6.7) |
| | 40 | 38.0, $CH_3$ | 3.17 (s) |

[a] Residues: Ala = alanine, *N*-Me-Dha = *N*-methyldehydroalanine, Pla = 3-phenyllactic acid, *N*-Ac-*β*-OH-Leu= *N*-acetyl-3-hydroxyleucine, *N*,*O*-$Me_2$-Thr = *N*,*O*-dimethylthreonine, *β*-OH-Leu = 3-hydroxyleucine, *N*-Me-Ala = *N*-methylalanine,. [b] overlapping resonances.

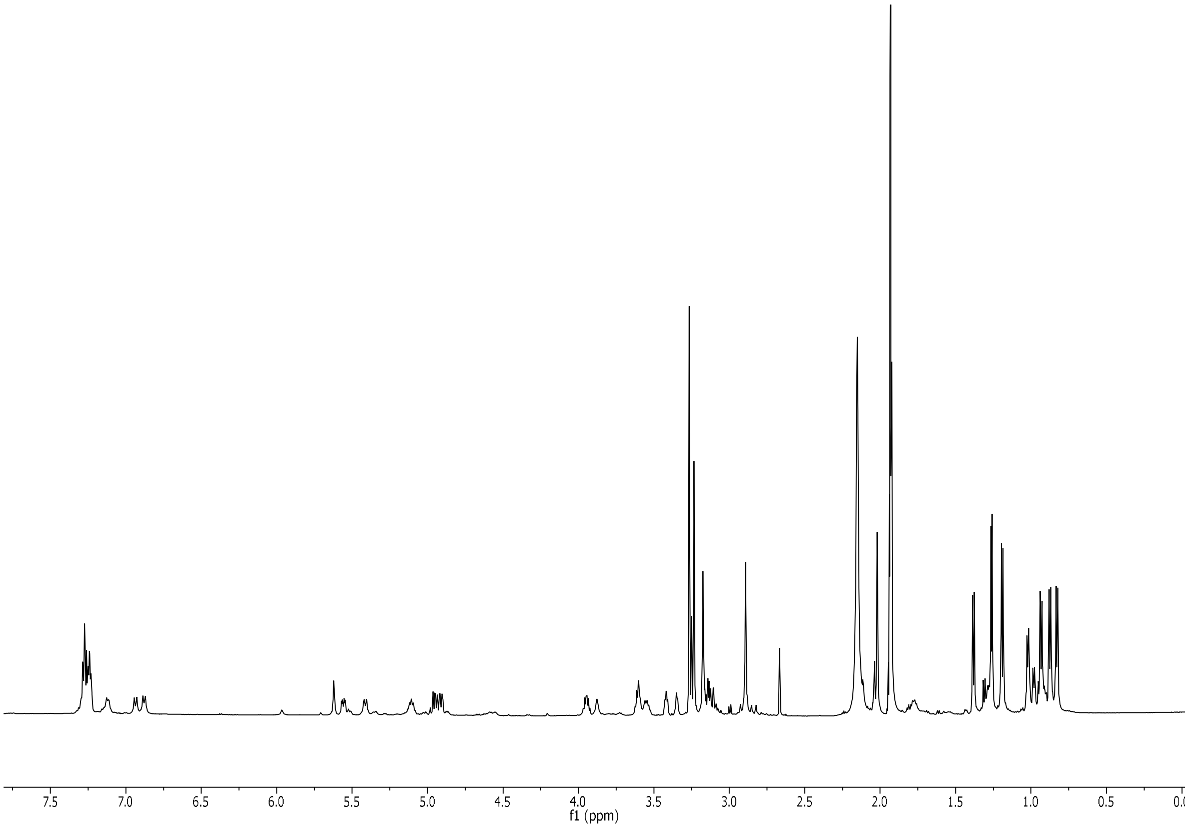


**Supplementary Figure 10:** $^1H$ NMR spectrum of compound **2** in acetonitrile-*$d_3$* (600 MHz).

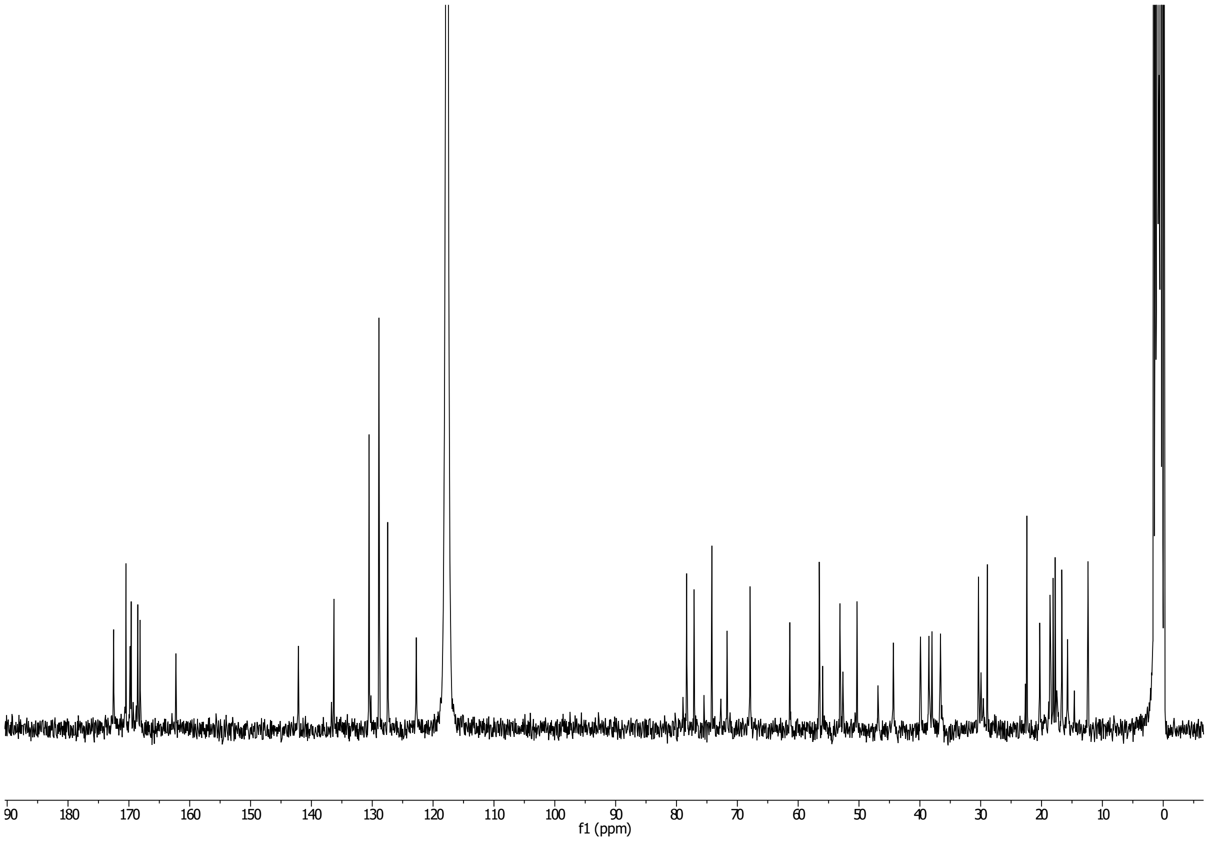


**Supplementary Figure 11:** $^{13}C$ NMR spectrum of compound **2** in acetonitrile-$d_3$ (150 MHz).

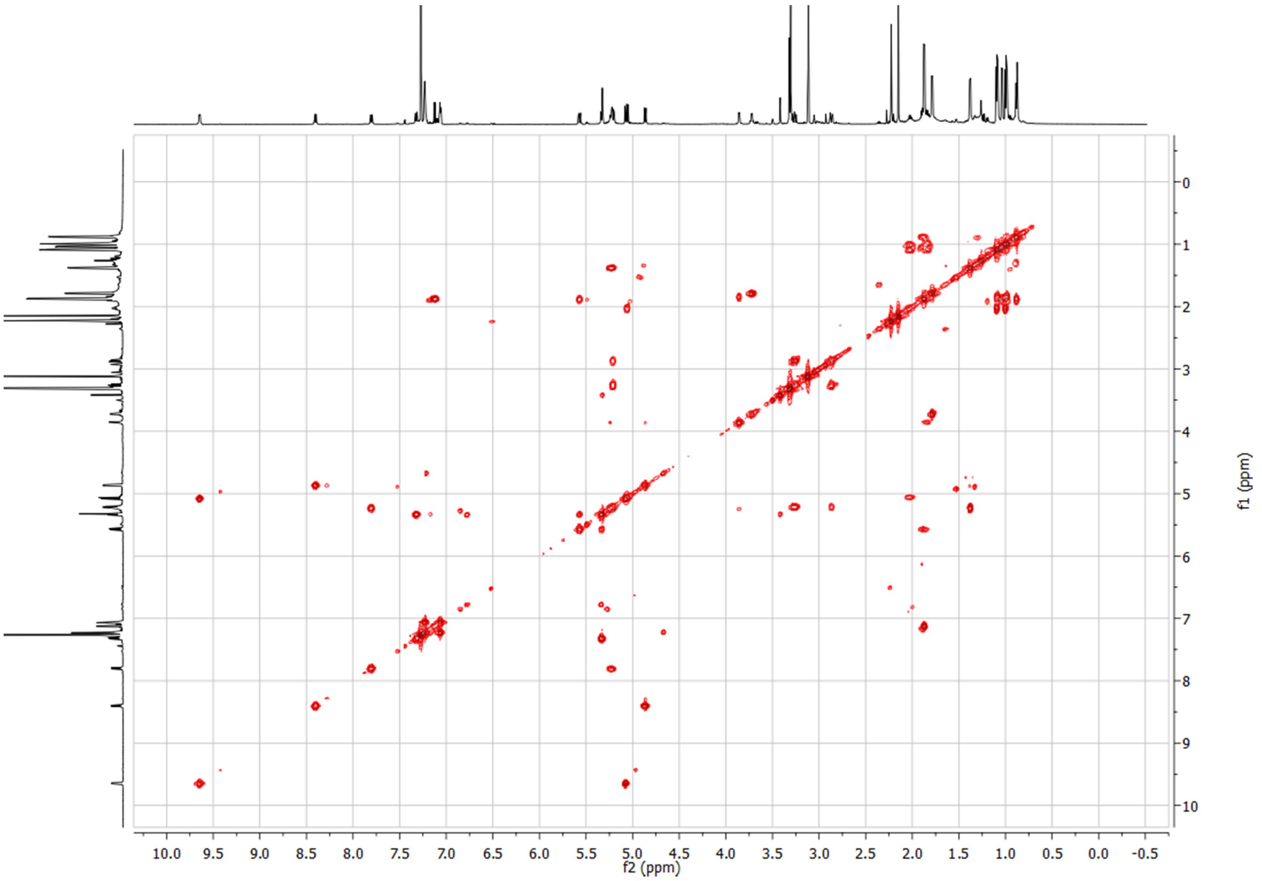


**Supplementary Figure 12:** $^{1}H$-$^{1}H$ COSY NMR spectrum of compound **2** in acetonitrile-$d_3$ (600 MHz).

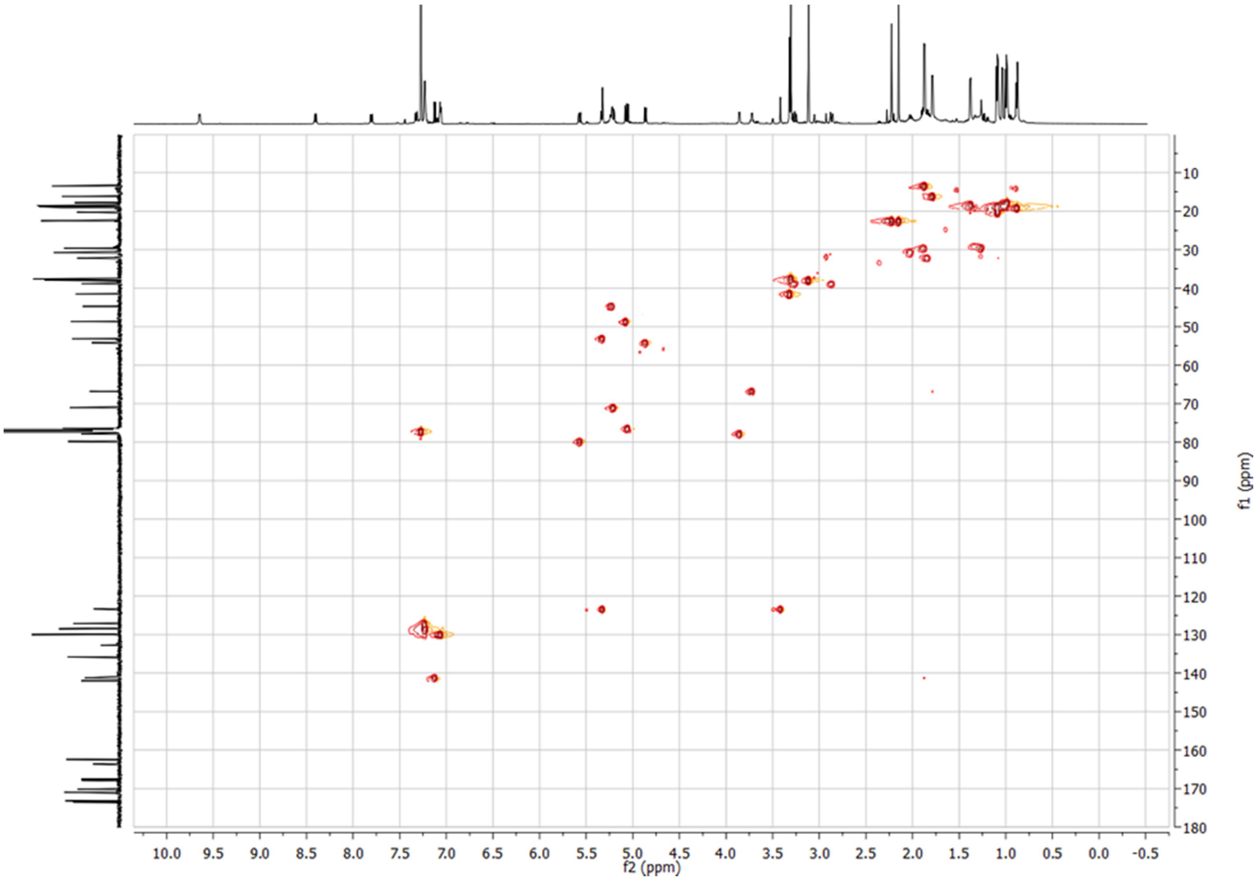


**Supplementary Figure 13:** $^{1}H$-$^{13}C$ HSQC NMR spectrum of compound **2** in acetonitrile-$d_3$ (600 MHz).

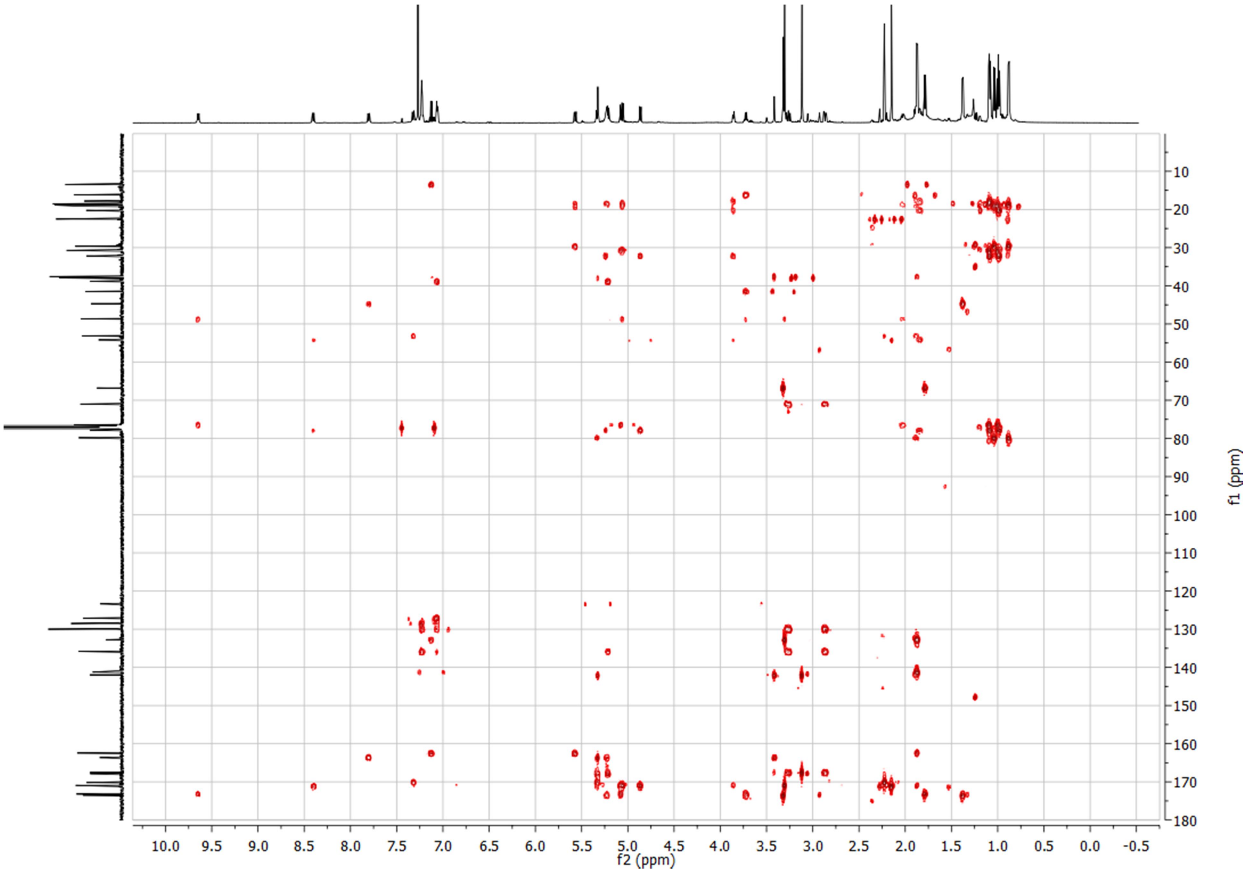


**Supplementary Figure 14:** $^{1}H$-$^{13}C$ HMBC NMR spectrum of compound **2** in acetonitrile-$d_3$ (600 MHz).

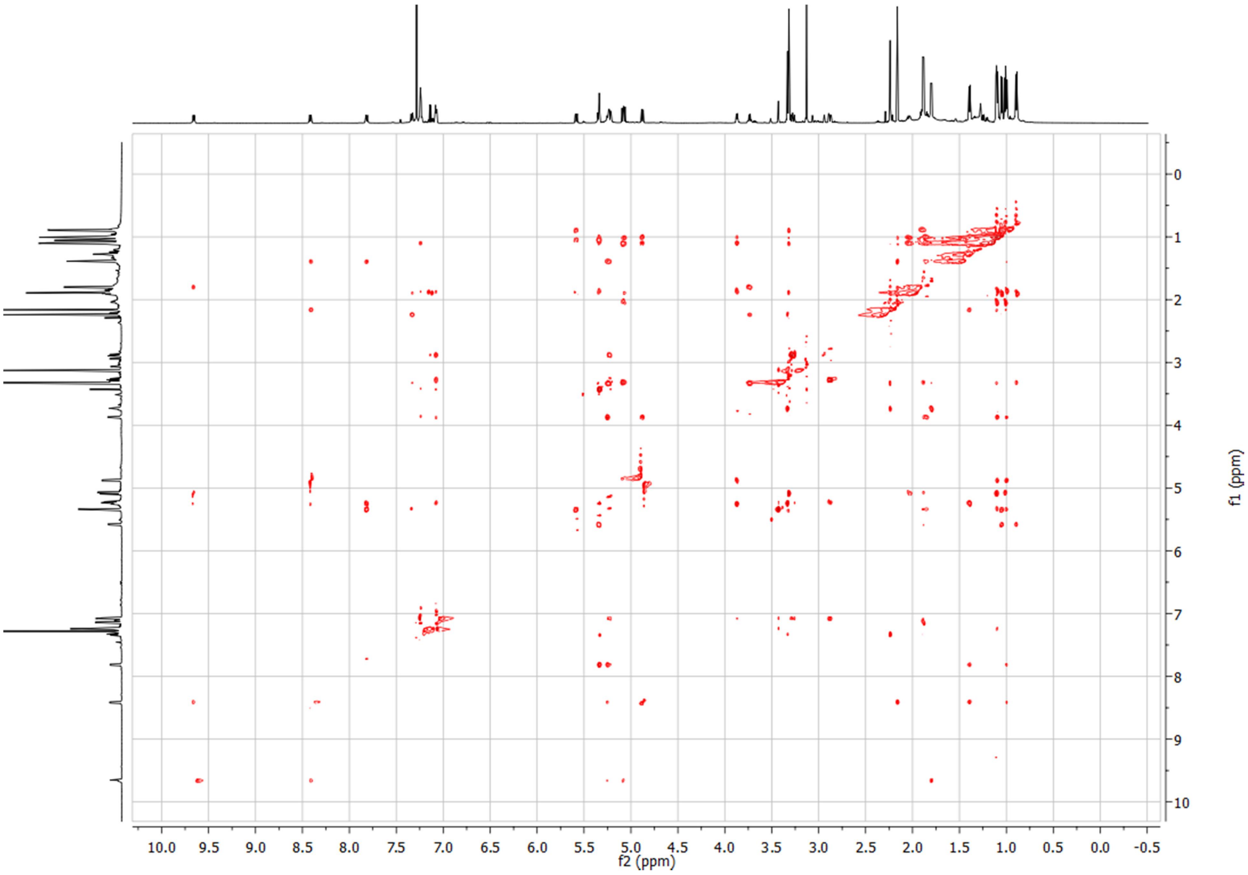


**Supplementary Figure 15:** $^{1}H$-$^{1}H$ ROESY NMR spectrum of compound **2** in acetonitrile-$d_3$ (600 MHz)

## *In vitro* Characterization of NRPS Domains and FrsH

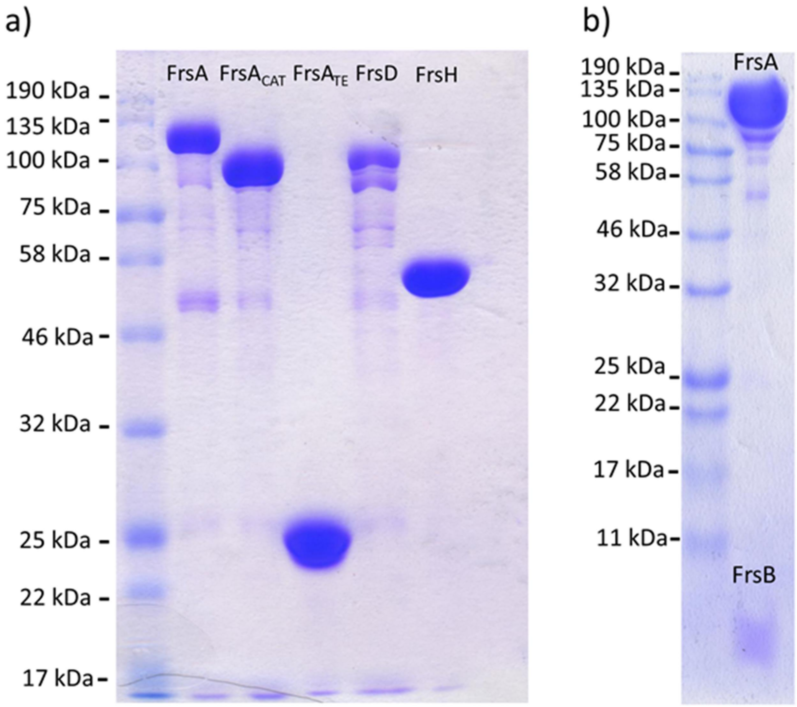


**Supplementary Figure 16: a) SDS-PAGE gel of FrsA, FrsD and FrsH constructs.** FrsA and FrsD were always coexpressed with the MbtH-like protein FrsB (not visible on the a) gel), which was crucial for solubility and activity of the NRPS proteins. These experiments were independently repeated at least three times with similar results. **b) SDS-PAGE gel of coexpressed FrsA and FrsB.** The FrsB, expressed without $His_6$-tag, coelutes in approximately 1:1 molar ratio with FrsA and is visible in SDS buffer systems for small proteins, Size FrsB: 8.12 kDa. These experiments were independently repeated at least three times with similar results.

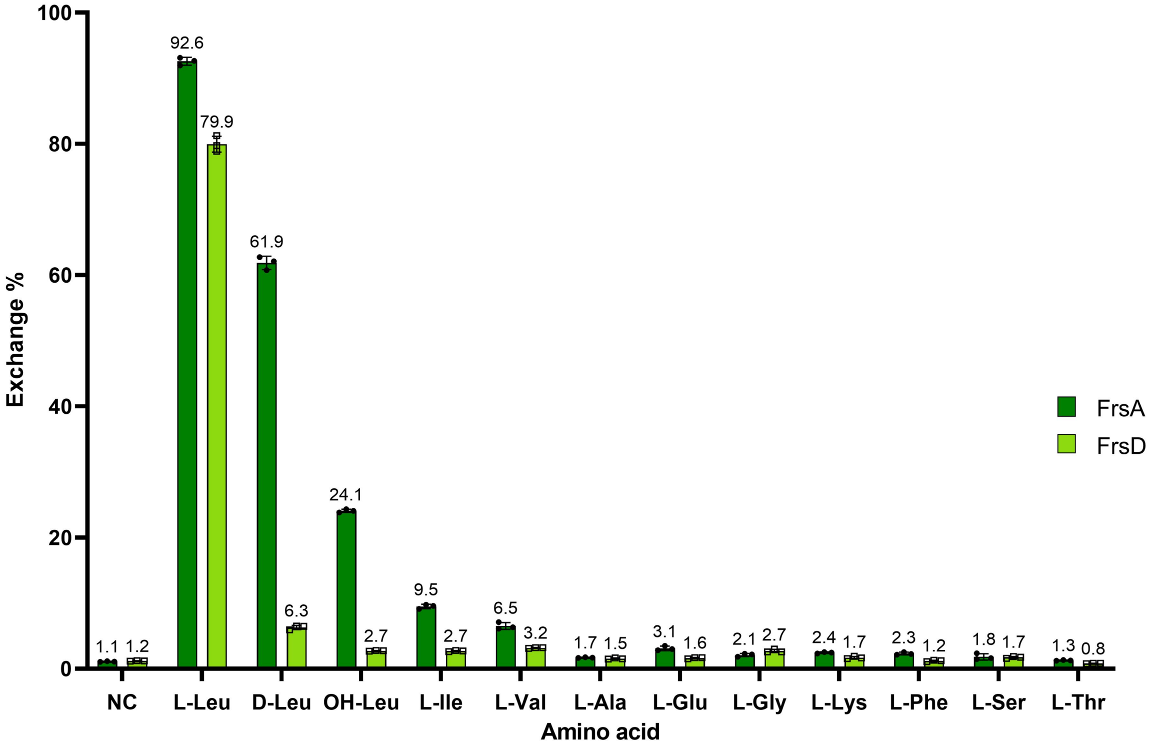


**Supplementary Figure 17: $^{18}O_4$-ATP exchange adenylation assay results for FrsA and FrsD, both coexpressed with FrsB**. Data are presented as mean values ± SD. All experiments were performed in triplicate.

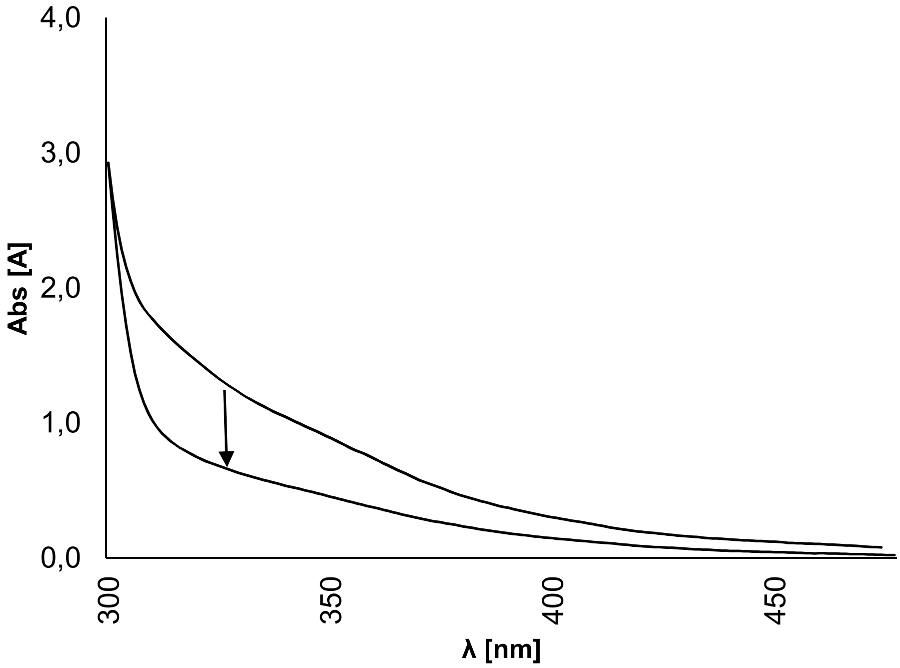


**Supplementary Figure 18: Absorption spectra of FrsH.** The iron cluster is visible as a broad feature between 300 – 400 nm in the ferrous state. Reduction to the ferric state diminishes the feature.

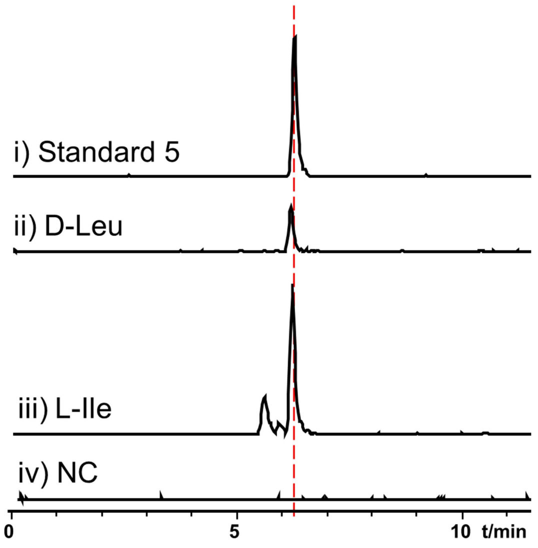


**Supplementary Figure 19:** EIC ($m/z$ = 202.108) showing production of **5**. i) **5**, 1 µg/mL. ii) Enzymatic assay with purified $FrsA_{CAT}$/FrsB, FrsH, incubated with D-Leu and propionyl-CoA, hydrolyzed with KOH. iii) Enzymatic assay with L-Ile v) negative control with heat inactivated proteins.

# Chemical synthesis of compounds 5 and 6

## General Procedures

Thin-layer chromatography was carried out on Merck (Darmstadt, Germany) aluminium sheets, silica gel 60 F254. Detection was performed with UV light at 254 nm. Preparative column chromatography was performed on Merck silica gel (0.063-0.200 mm, 60 Å). Melting points were determined on a Büchi (Essen, Germany) 510 oil bath apparatus. $^{1}$H NMR (500 MHz) and $^{13}$C NMR (125 MHz) spectra were recorded on a Bruker Avance DRX 500. Chemical shifts δ are given in ppm referring to the signal centre using the solvent peaks for reference: DMSO-$d_6$ 2.49/39.7 ppm. LC-MS analyses were carried out on an API2000 (Applied Biosystems, Darmstadt, Germany) mass spectrometer coupled to an Agilent (Santa Clara, CA, USA) 1100 LC system using a EC50/2 Nucleodur C18 Gravity column (Macherey-Nagel, Düren, Germany; 50 × 2.0 mm, particle size 3 µm). Purity of the compounds was determined using the diode array detector (DAD) of the LC-MS instrument between 200 and 400 nm. HRMS were recorded on a microTOF-Q (Bruker, Köln, Germany) mass spectrometer connected to a Dionex (Thermo Scientific, Braunschweig, Germany) Ultimate 3000 LC via an ESI interface using a Nucleodur $C_{18}$ Gravity column (50 × 2.0 mm I.D., 3 µm, Macherey-Nagel, Düren, Germany).

## (2*S*,3*R*)-3-Hydroxy-4-methyl-2-propionamidopentanoic acid (5).

A stirred solution of propanoic acid (370 mg, 5.00 mmol) and *N*-methylmorpholine (506 mg, 5.00 mmol) in THF (8.6 mL) was cooled to -10 °C. Isobutyl chloroformate (683 mg, 5.00 mmol) was added and the reaction was allowed to stir for 0.5 h. The temperature was adjusted to 0 °C, followed by treatment with (2*S*,3*R*)-2-amino-3-hydroxy-4-methylpentanoic acid (1.10 g, 7.50 mmol) in 1 M NaOH (5.2 mL). Since excess of base was avoided, racemization was prevented After stirring the reaction for further 24 h at room temperature, the mixture was diluted with $H_2O$ (16 mL) and washed with ethyl acetate (2 × 16 mL). The combined ethyl acetate layer was extracted with sat. aq. $NaHCO_3$ solution (3 × 16 mL). All aqueous layers were combined, adjusted to pH ~2 by adding 1 M HCl and extracted with ethyl acetate (3 × 32 mL). This combined organic layer was dried over $Na_2SO_4$, filtered and evaporated to dryness. The crude residue was purified by column chromatography on silica gel using a gradient of petroleum ether/ethyl acetate (1:1) + 1% AcOH to 100% ethylacetate + 1% AcOH to give a white solid (762 mg, 75%); mp 104–106 °C.

$^{1}$H NMR (500 MHz, DMSO-$d_6$) δ 0.77 (d, $^{3}J$ = 6.7 Hz, 3H) and 0.90 (d, $^{3}J$ = 6.6 Hz, 3H, $CH(CH_3)_2$), 0.98 (t, $^{3}J$ = 7.6 Hz, 3H, $CH_2CH_3$), 1.50 – 1.58 (m, 1H, $CH(CH_3)_2$), 2.12 – 2.20 (m, 2H, $CH_2$), 3.50 (dd, $^{3}J$ = 8.7 Hz, $^{3}J$ = 2.8 Hz, 1H, *CH*-OH), 4.40 (dd, $^{3}J$ = 9.1 Hz, $^{3}J$ = 2.8 Hz, 1H, *CH*-NH), 7.53 (d, $^{3}J$ = 9.1 Hz, 1H, NH). Two proton signals ($CO_2H$, OH) do not appear. $^{13}$C NMR (125 MHz, DMSO-$d_6$) δ 10.08 ($CH_2CH_3$), 19.12, 19.21 ($CH(CH_3)_2$), 28.48 ($CH(CH_3)_2$), 30.92 ($CH_2CH_3$), 54.54 (CHNH), 76.18 (CHOH), 173.09, 173.30 ($CO_2H$, CONH). LC-MS (ESI) (90% $H_2O$ to 100% MeOH in 10 min, then 100% MeOH for 10 min, DAD 196–400 nm), *m/z* = 204.0 ($[M+H]^+$).

### (2*S*,3*R*)-*S*-2-Acetamidoethyl 3-hydroxy-4-methyl-2-propionamidopentanethioate (6).

(2*S*,3*R*)-3-Hydroxy-4-methyl-2-propionamidopentanoic acid (**5**, 610 mg, 3.00 mmol) was dissolved in anhydrous acetonitrile (120 mL) under nitrogen atmosphere. A solution of DCC (650 mg, 3.15 mmol) and HOBt × $H_2O$ (482 mg, 3.15 mmol) in acetonitrile (120 mL) was slowly added, followed by *N*-acetylcysteamine (375 mg, 3.15 mmol). The reaction mixture was stirred at room temperature for 24 h. Subsequently, the urea was filtered off and the filtrate was evaporated to dryness. The crude residue was purified by preparative column chromatography using ethyl acetate/MeOH (9:1) as eluent to obtain clear oil (82 mg, 9%).

$^1$H NMR (500 MHz, DMSO-$d_6$) δ 0.74 (d, $^3J$ = 6.7 Hz, 3H) and 0.90 (d, $^3J$ = 6.6 Hz, 3H, CH(C$H_3$)$_2$), 1.02 (t, $^3J$ = 7.6 Hz, 3H, $CH_2CH_3$), 1.53 – 1.61 (m, 1H, C*H*($CH_3$)$_2$), 1.78 (s, 3H, $COCH_3$), 2.26 (q, $^3J$ = 7.6 Hz, 2H, C$H_2$$CH_3$), 2.79 – 2.89 (m, 2H, $CH_2$S), 3.08 – 3.19 (m, 2H, NHC$H_2$), 3.59 (ddd, $^3J$ = 9.1 Hz, $^3J$ = 6.9 Hz, $^3J$ = 2.4 Hz, 1H, C*H*OH), 4.54 (dd, $^3J$ = 8.9 Hz, $^3J$ = 2.4 Hz, 1H, C*H*NH), 5.01 (d, $^3J$ = 6.9 Hz, 1H, OH), 7.98 (t, $^3J$ = 5.7 Hz, 1H, N*H*$CH_2$), 8.08 (d, $^3J$ = 8.9 Hz, 1H, CHN*H*). $^{13}$C NMR (125 MHz, DMSO-$d_6$) δ 9.90 ($CH_2$*C*$H_3$), 18.83, 19.17 (CH(*C*$H_3$)$_2$), 22.66 (*C*$H_3$CO), 27.99 (S$CH_2$), 28.39 (*C*H($CH_3$)$_2$), 30.99 (*C*$H_2$$CH_3$), 38.24 (NH$CH_2$), 61.53 (CHNH), 76.00 (CHOH), 169.44 ($H_3$C*C*ONH), 174.03 ($CH_2$*C*ONH), 201.86 (COS). LC-MS (ESI) (90% $H_2O$ to 100% MeOH in 10 min, then 100% MeOH for 10 min, DAD 220–400 nm), *m/z* = 305.0 ([M+H]$^+$). HRMS, calcd. for $C_{13}H_{24}N_2O_4S$: [M+H]$^+$ *m/z* 305.1530; found: 305.1544.

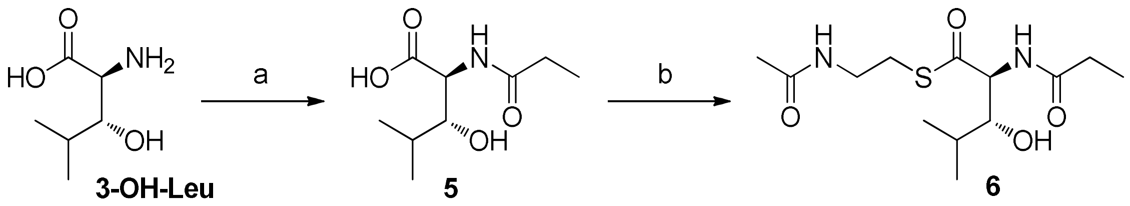


**Supplementary Figure 20**: Synthesis of the *N*-Pp-Hle SNAC thioester **6**. Reagents and conditions: (a) propanoic acid, $ClCO_2$*i*-Bu, NMM, THF, 1M NaOH, -10 °C (0.5 h) to rt (24 h); (b) N-acetylcysteamine, DCC, HOBt × $H_2O$, MeCN, rt, 24 h, $N_2$.

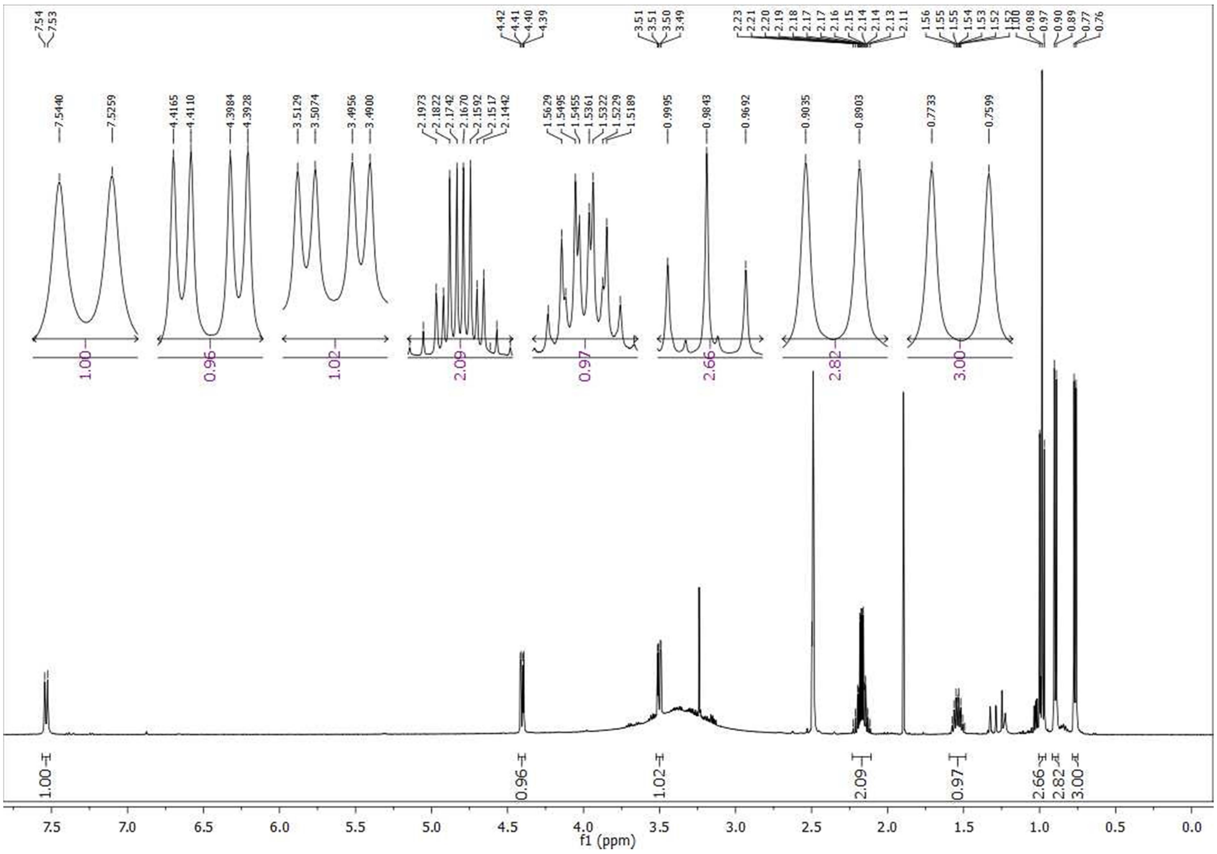

**Supplementary Figure 21:** $^{1}$H NMR spectrum of compound **5** in DMSO-$d_6$ (500 MHz).

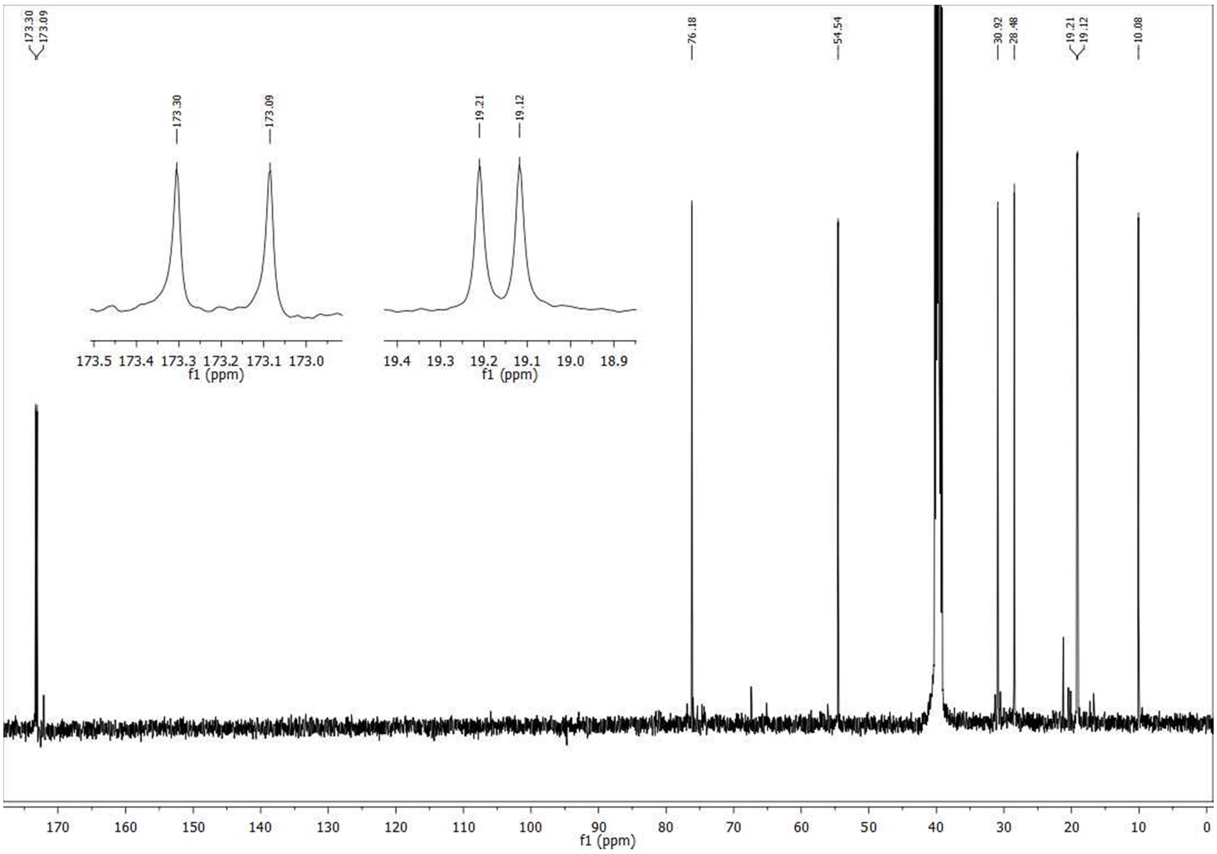

**Supplementary Figure 22:** $^{13}$C NMR spectrum of compound **5** in DMSO-$d_6$ (125 MHz).

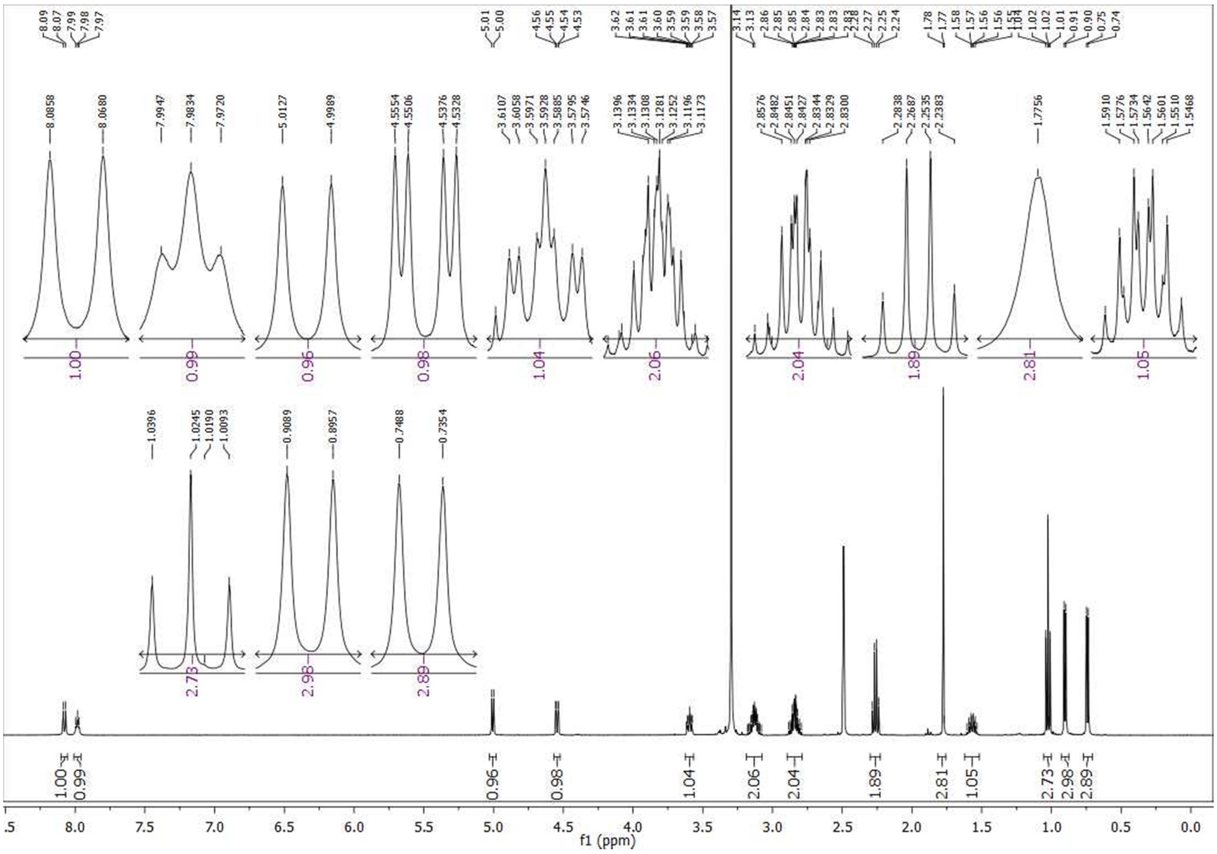


**Supplementary Figure 23:** $^{1}$H NMR spectrum of compound **6** in DMSO-$d_6$ (500 MHz).

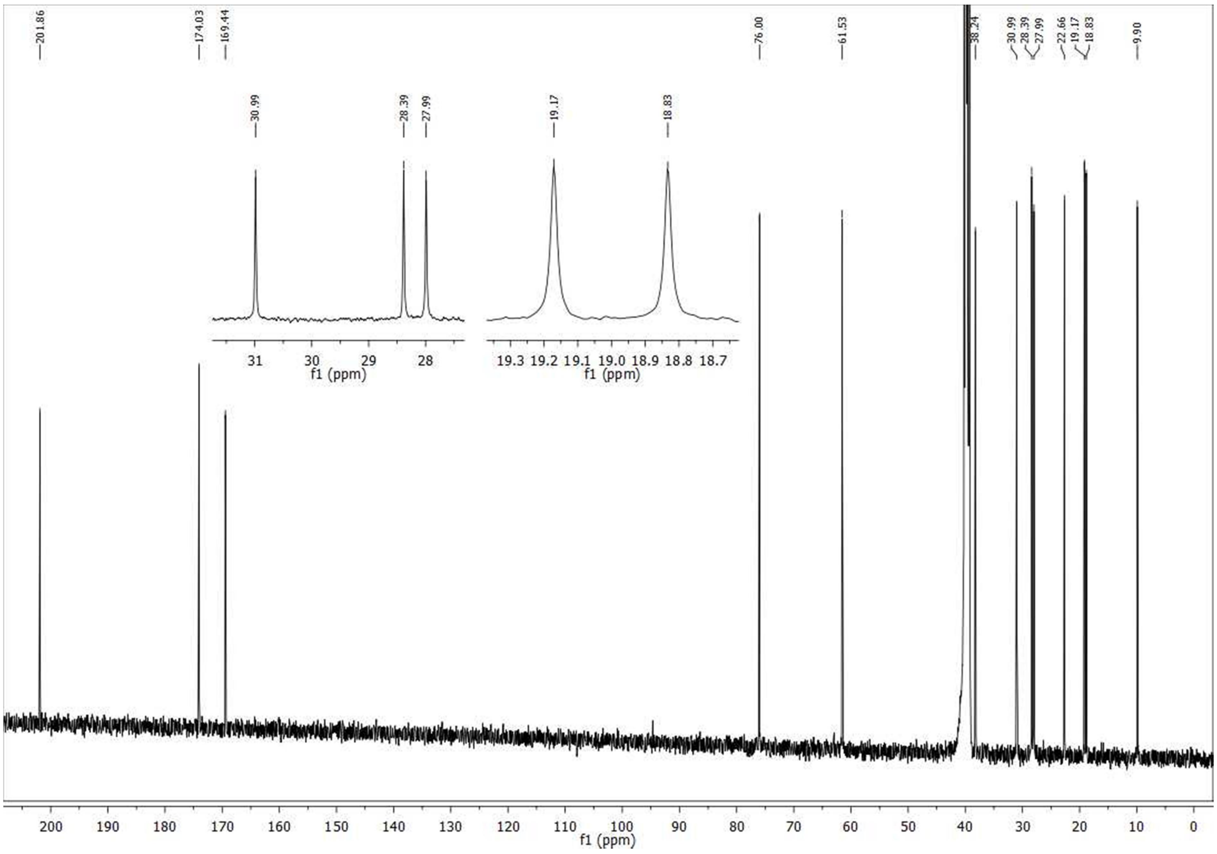


**Supplementary Figure 24:** $^{13}$C NMR spectrum of compound **6** in DMSO-$d_6$ (125 MHz).

## Structure Elucidation of FR-5 (7)

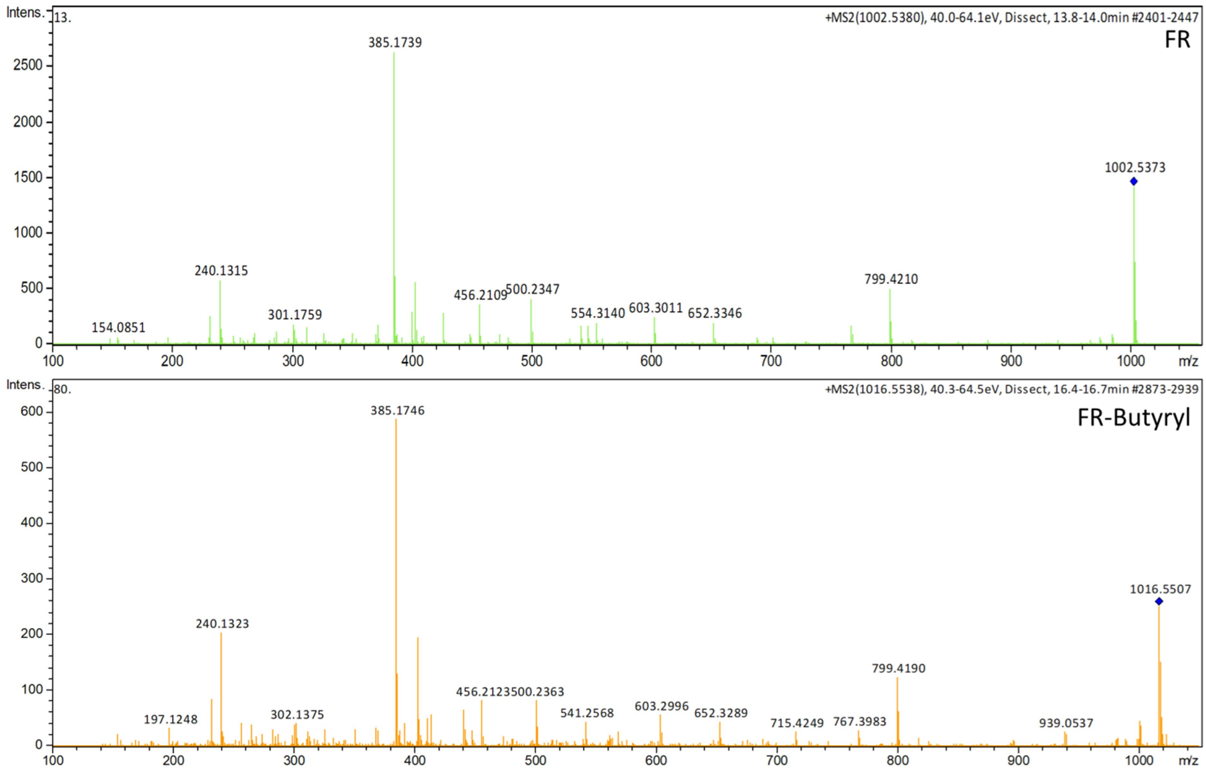


**Supplementary Figure 25: MS/MS spectra of 1 (*m/z:* 1002.54) and 7 (*m/z*: 1016.55).** From *m/z:* 799.42 (loss of side chain, and loss of $H_2O$, see Ref. 9), the compounds have the same fragmentation pattern, indicating, that the methylation of FR (M+14 Da) is in the side chain and stems most likely from the incorporation of a butyryl group from the precursor butyryl-CoA instead of the propionyl residue in FR.

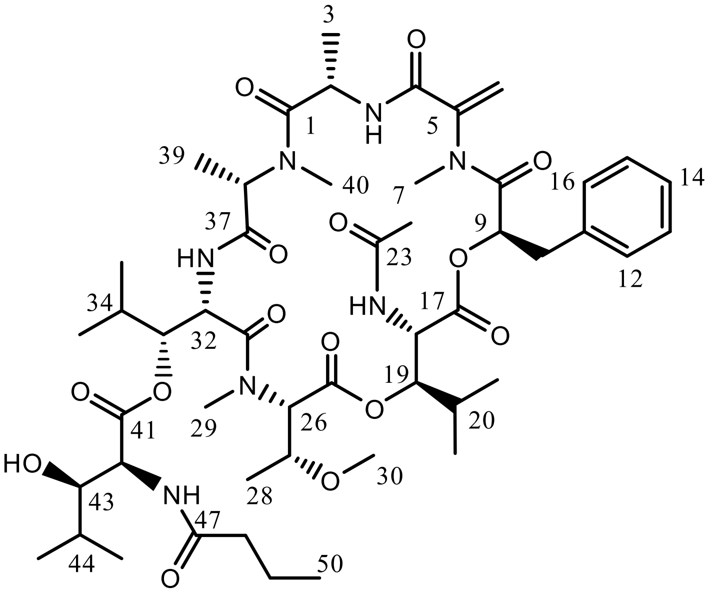


**Supplementary Figure 26**: **Chemical structure of FR-5** (**7**). Carbon atoms are numbered.

**Supplementary Table 5:** $^{1}$H and $^{13}$C NMR spectroscopic data of compound **7** in $CDCl_3$ ($^{1}$H: 300 MHz; $^{13}$C: 75 MHz).

| Residue[c] | No C/H[b] | $\delta_C$[a], mult | $\delta_H$[a] (mult, *J* in Hz) | COSY | HMBC |
|---|---|---|---|---|---|
| Ala | 1 | 172.5, C | – | | |
| | 2 | 45.7, CH | 4.90 (m) | 3, 2-NH | 1, 3, 4 |
| | 2-NH | – | 8.53 (d, 9.1) | 2 | 2, 4 |
| | 3 | 18.0, $CH_3$ | 1.38 (d, 6.7) | 2 | 1, 2 |
| *N*-MeDha | 4 | 163.9, C | – | | |
| | 5 | 145.3, C | – | | |
| | 6 | 106.7, $CH_2$ | a 5.31 (brs) | 6b | 5 |
| | | – | b 5.07 (brs) | 6a | 5 |
| | 7 | 36.2, $CH_3$ | 3.13 (s) | | 5, 8 |
| D-Pla | 8 | 167.7, C | – | | |
| | 9 | 72.6, CH | 5.20 (dd, 4.2, 8.3) | 10a, 10b | 8, 10, 17 |
| | 10 | 36.6, $CH_2$ | a 3.08 (dd, 4.2, 14.8) | 9, 10b | 9, 11, 12/16 |
| | | – | b 2.97 (dd, 8.3, 14.8) | 10a | 9, 11, 12/16 |
| | 11 | 136.0, C | – | | |
| | 12/16 | 129.6, CH | 7.24[d] | 13/15 | 10, 14 |
| | 13/15 | 128.6, CH | 7.27[d] | 14, 12/16 | 11 |
| | 14 | 126.9, CH | 7.23[d] | 13/15 | 12/16 |
| *N*-Ac-β- | 17 | 169.2, C | – | | |
| OH-Leu | 18 | 50.3, CH | 5.24 (brd, 10.0) | 18-NH, 19 | 17, 19 |
| | 18-NH | – | 7.55, (d, 10,0) | 18 | 18, 23 |
| | 19 | 77.7, CH | 5.10 (brd, 10.0) | 18, 20 | 20, 25 |
| | 20 | 28.8, CH | 1.86 (m) | 19, 21, 22 | |
| | 21 | 18.9, $CH_3$ | 1.01 (d, 6.8) | 20 | 20, 22 |
| | 22 | 18.8, $CH_3$ | 0.85 (d, 6.8) | 20 | 20, 21 |
| | 23 | 171.4, C | – | | |
| | 24 | 22.5, $CH_3$ | 2.21 (s) | | 23 |
| *N*-MeThr(*O*Me) | 25 | 166.5 C | – | | |
| | 26 | 64.4, CH | 4.05 (d, 9.6) | 27 | 25 |
| | 27 | 72.3, CH | 3.74 (m) | 26, 28 | 26, 28 |
| | 28 | 16.3, $CH_3$ | 1.16 (d, 5.8) | 27 | 26, 27 |
| | 29 | 28.7, $CH_3$ | 2.68 (s) | | 26, 31 |
| | 30 | 57.2, $CH_3$ | 3.40 (s) | | 27 |
| β-OH-Leu | 31 | 171.2, C | – | | |
| | 32 | 46.6, CH | 5.35 (d, 9.9) | 32-NH, 33 | 31, 33 |
| | 32-NH | | 6.74, (d, 9.9) | 32 | 32, 37 |
| | 33 | 77.0, CH | 5.30, (d, 10.0) | 32, 34 | 41 |
| | 34 | 30.5, CH | 1.70 (m) | 33, 35, 36 | 35, 36 |
| | 35 | 19.4, $CH_3$ | 1.08 (d, 6.7) | 34 | 33, 34, 36 |
| | 36 | 18.3, $CH_3$ | 0.82 (d, 6.7) | 34 | 33, 34, 35 |
| *N*-MeAla | 37 | 169.9, C | – | | |
| | 38 | 56.4, CH | 4.70 (q, 6.8) | 39 | 37, 39 |
| | 39 | 14.3, $CH_3$ | 1.37 (d, 6.8) | 38 | 37, 38 |
| | 40 | 31.4, $CH_3$ | 2.87 (s) | | 1, 38 |
| *N*-But-β- | 41 | 170.2, C | – | | |
| OH-Leu | 42 | 56.8, CH | 4.55 (brd, 7.8) | 42-NH | 41 |
| | 42-NH | – | 7.17 (d, 7.8) | 42 | 42, 47 |
| | 43 | 78.2, CH | 3.71 (m) | 43-OH, 44 | 41, 44 |
| | 43-OH | – | 6.87 (d, 4.2) | 43 | |
| | 44 | 30.0, CH | 1.96 (m) | 43, 45, 46 | 45, 46 |
| | 45 | 20.5, $CH_3$ | 1.15 (6.7) | 44 | 43, 44, 46 |
| | 46 | 18.5, $CH_3$ | 0.85 (d, 6.7) | 44 | 43, 44, 45 |
| | 47 | 173.9, C | – | | |
| | 48 | 37.4, $CH_2$ | 2.47, m | 49 | |
| | 49 | 19.1, $CH_2$ | 1.68, m | 48, 50 | 50 |
| | 50 | 13.8, $CH_3$ | 0.95 (t, 7.5) | 49 | 48, 49 |

[**a**]Assignments are based on extensive 1D and 2D NMR measurements (HMBC, HSQC, COSY). $^{13}$C-NMR spectra were recorded at 75 MHz. [**b**]Numbers according to Supplementary Figure 24. [**c**] Residues: Ala = alanine, *N*-MeDha = *N*-methyldehydroalanine, D-Pla = D-3-phenyllactic acid, *N*-Ac-β-OH-Leu= *N*-acetylhydroxyleucine, *N*-MeThr(OMe) = *N*,*O*-dimethylthreonine, β-OH-Leu = β-hydroxyleucine, *N*-MeAla = *N*-methylalanine, *N*-But-β-OH-Leu = *N*-butyryl-β-hydroxyleucine.[d] overlaying resonances

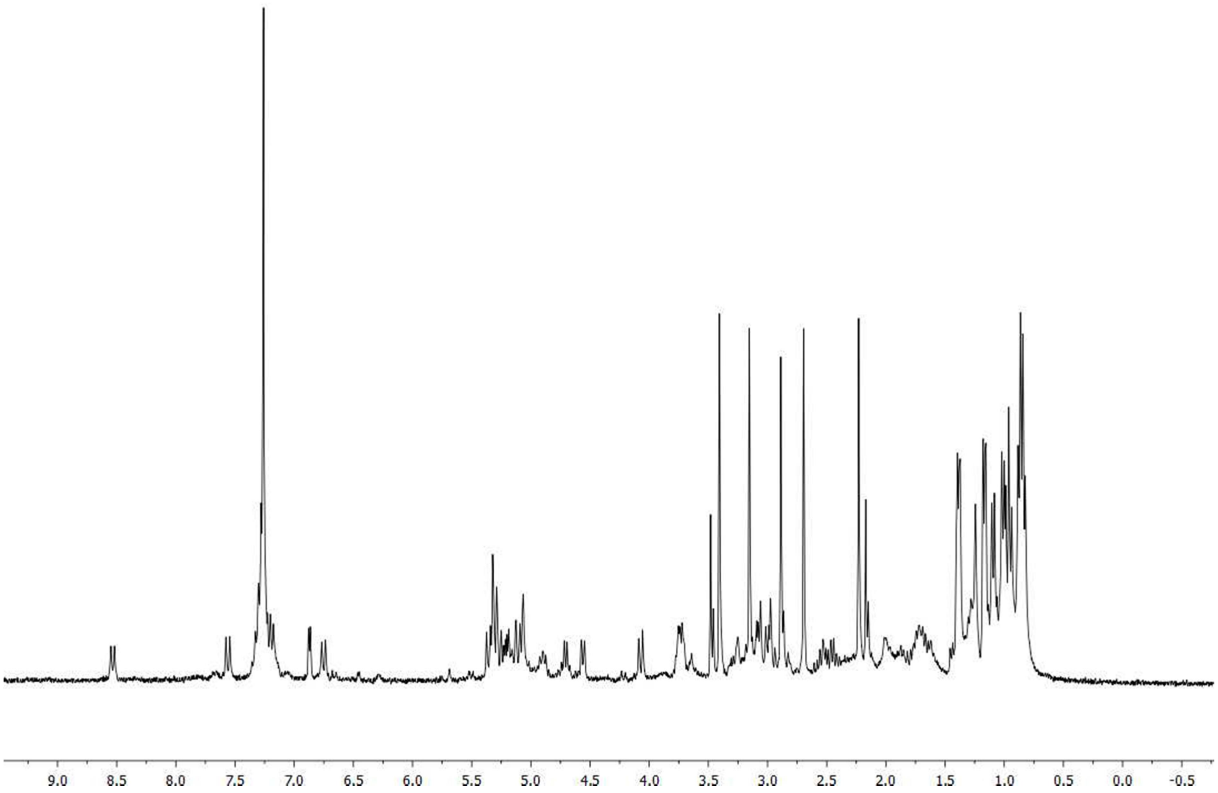


**Supplementary Figure 27**: $^{1}H$ NMR spectrum of compound **7** in $CDCl_3$ (300 MHz).

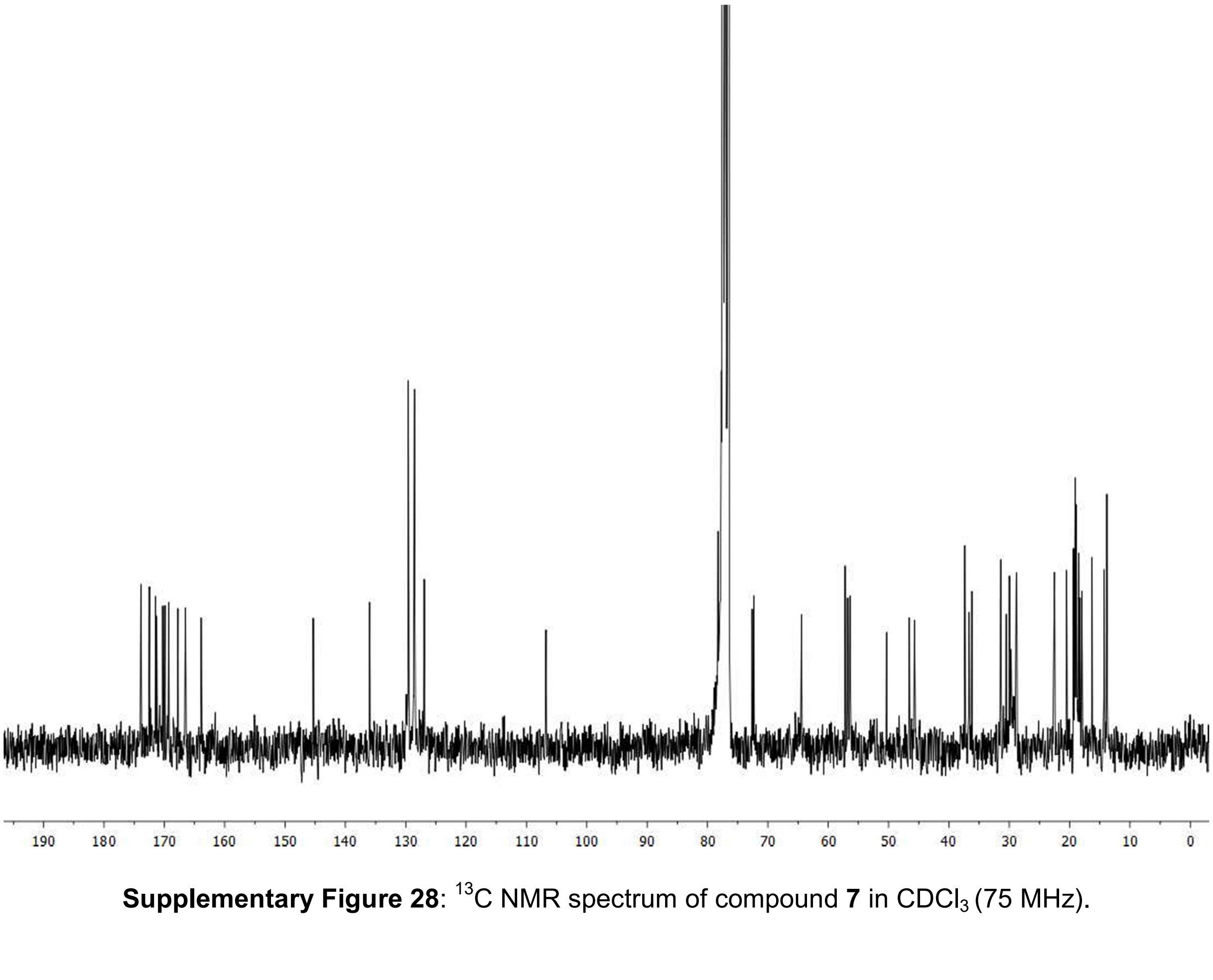


**Supplementary Figure 28**: $^{13}C$ NMR spectrum of compound **7** in $CDCl_3$ (75 MHz).

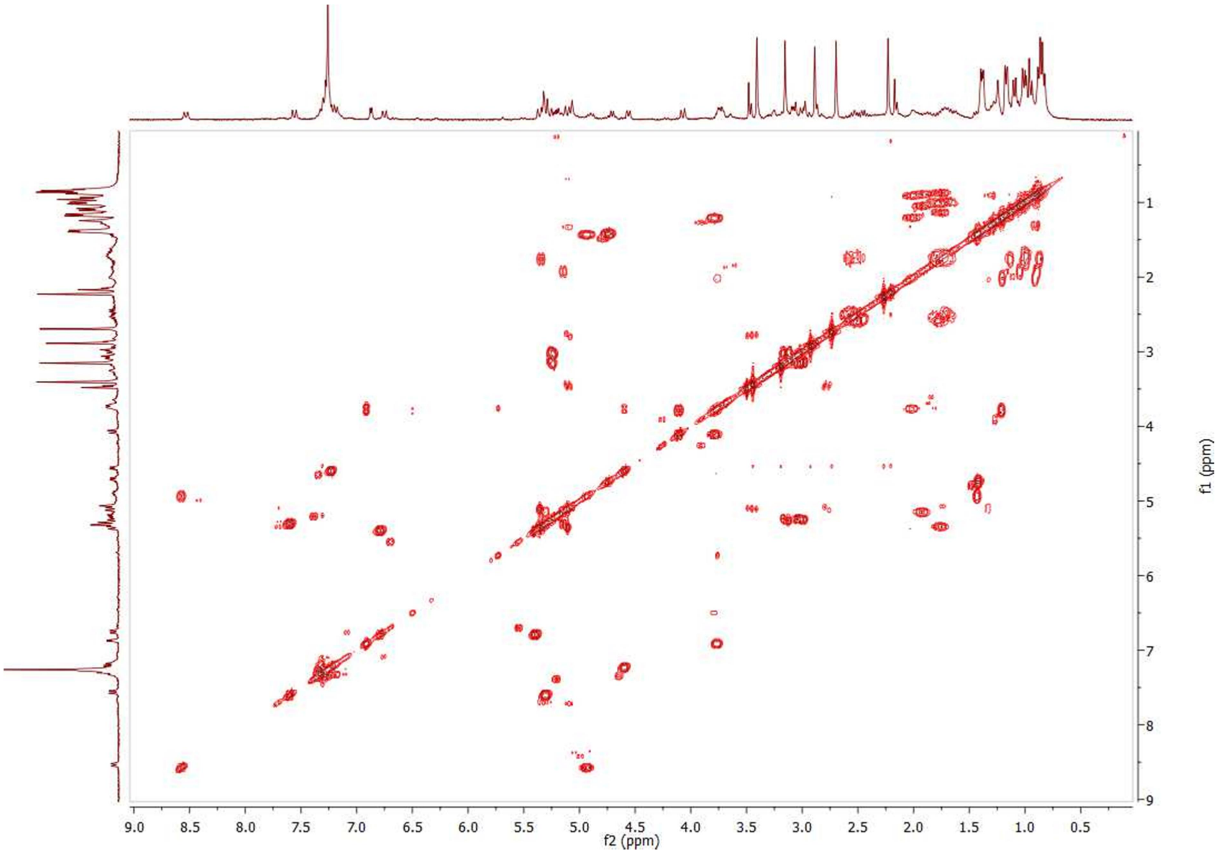


**Supplementary Figure 29**: $^{1}H$-$^{1}H$ COSY NMR spectrum of compound **7** in $CDCl_3$ (300 MHz).

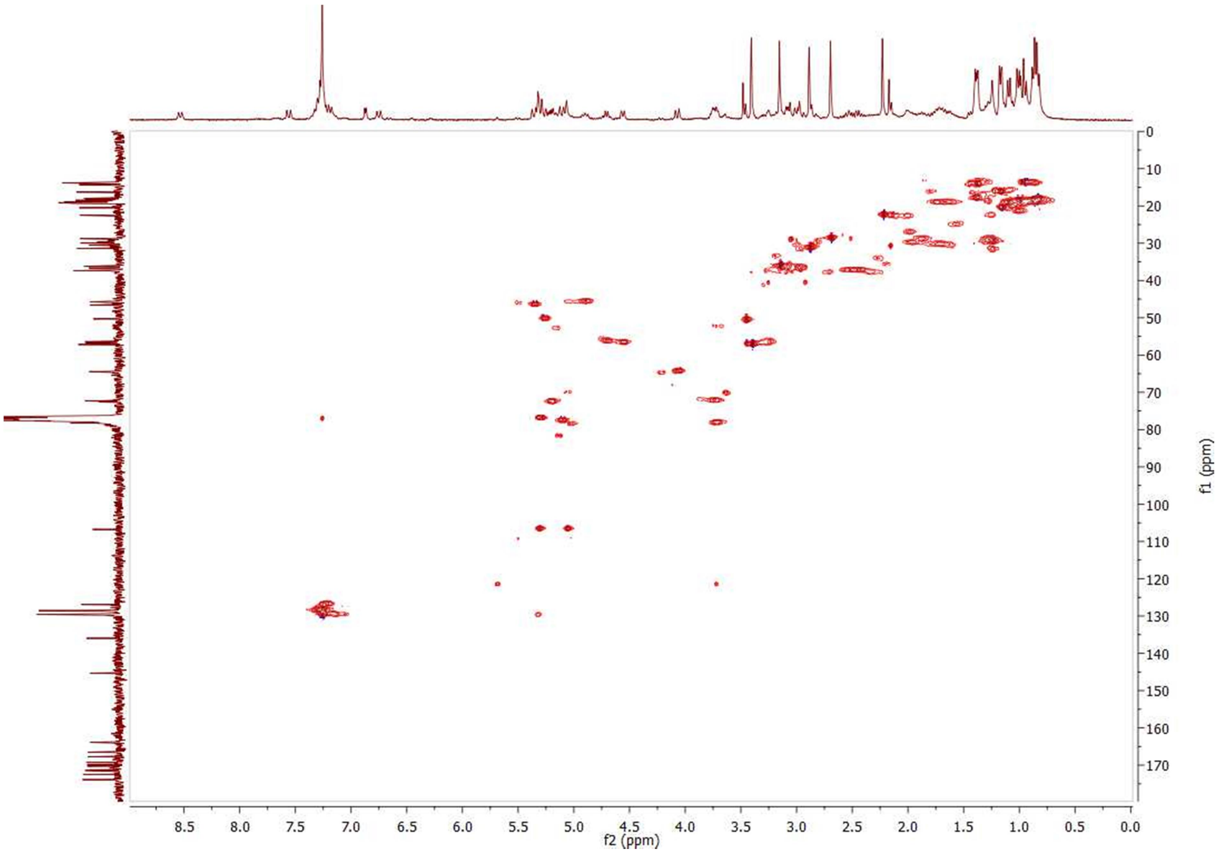


**Supplementary Figure 30**: $^{1}H$-$^{13}C$ HSQC NMR spectrum of compound **7** in $CDCl_3$ (300 MHz).

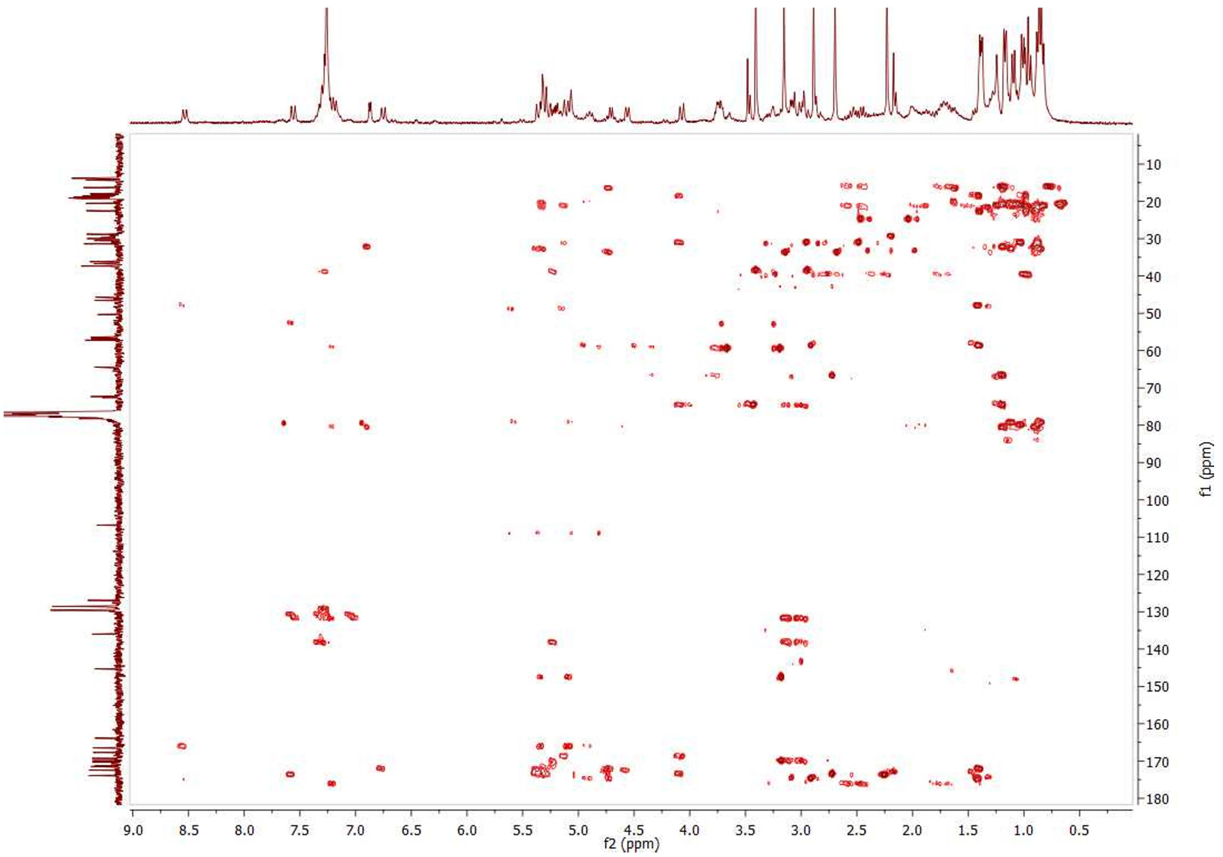


**Supplementary Figure 31**: $^{1}$H-$^{13}$C HMBC NMR spectrum of compound **7** in $CDCl_3$ (300 MHz).

## Gq Inhibition Assays

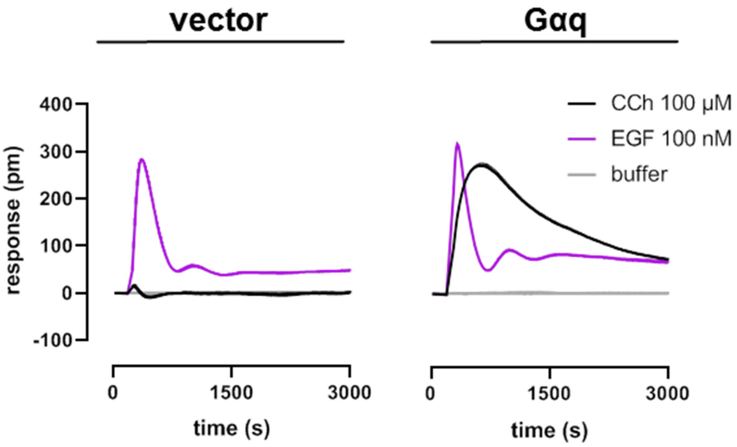


**Supplementary Figure 32: CRISPR-Cas9 HEK293 Gαq/Gα11-null cells were unresponsive to carbachol.** DMR analysis of whole cell responses evoked by epidermal growth factor (EGF, as viability control) and carbachol (CCh), which activates Gαq-sensitive endogenous muscarinic M3 receptors at the indicated concentrations in CRISPR-Cas9 genome-edited HEK293 cells that lack functional alleles for Gαq and Gα11. Shown are real-time measurements (mean + s.e.m., technical triplicates) representative of three such experiments.

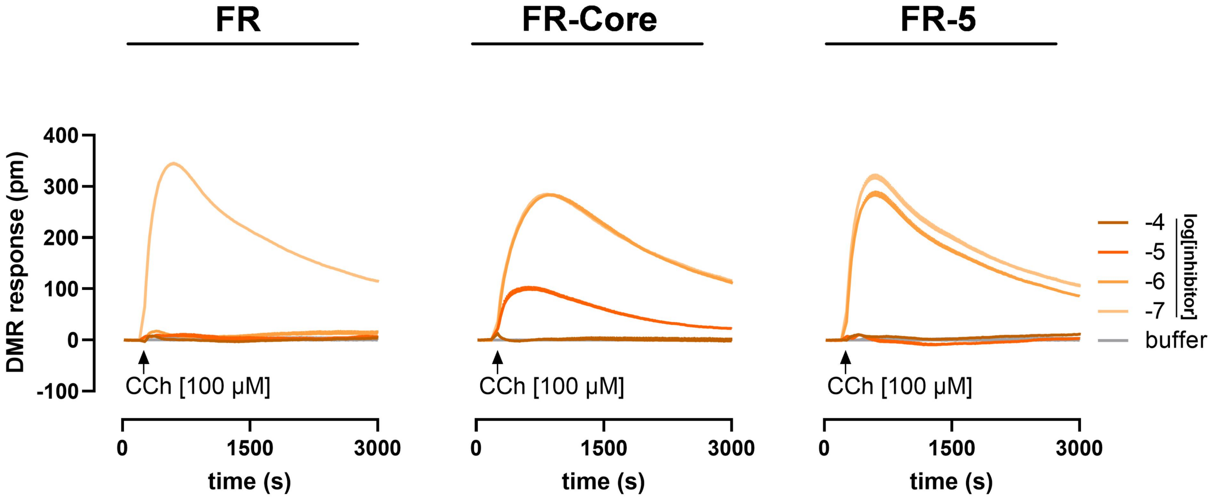


**Supplementary Figure 33: Pharmacological characterization of FR-Core and FR-5 on Gαq-mediated signaling.** Concentration-dependent inhibition of cell responses induced with carbachol (CCh) [100 µM] by FR, FR-Core and FR-5 in HEK293 Gαq/Gα11-null cells transfected to express wild type Gαq. Data shown are representative real-time recordings (mean + s.e.m., technical triplicates) of at least four independent experiments.

**Supplementary Table 6:** Quantification of FR, FR-Core and FR-5 inhibitory activities at wild type Gαq in HEK Gαq/Gα11-null cells. $IC_{50}$ values were determined by nonlinear regression on concentration-effect data and represent the mean of 'n' independent biological replicates performed as technical triplicates.

| # | $pIC_{50}$± s.e.m. | $IC_{50}$ [µM] | n |
|---|---|---|---|
| FR (**1**) | 6.34±0.03 | 0.45 | 11 |
| FR-Core (**2**) | 5.13±0.04 | 7.34 | 4 |
| FR-5 (**7**) | 5.50±0.06 | 3.18 | 4 |

# Bioinformatic Analyses on *frs* BGC and FrsA domains

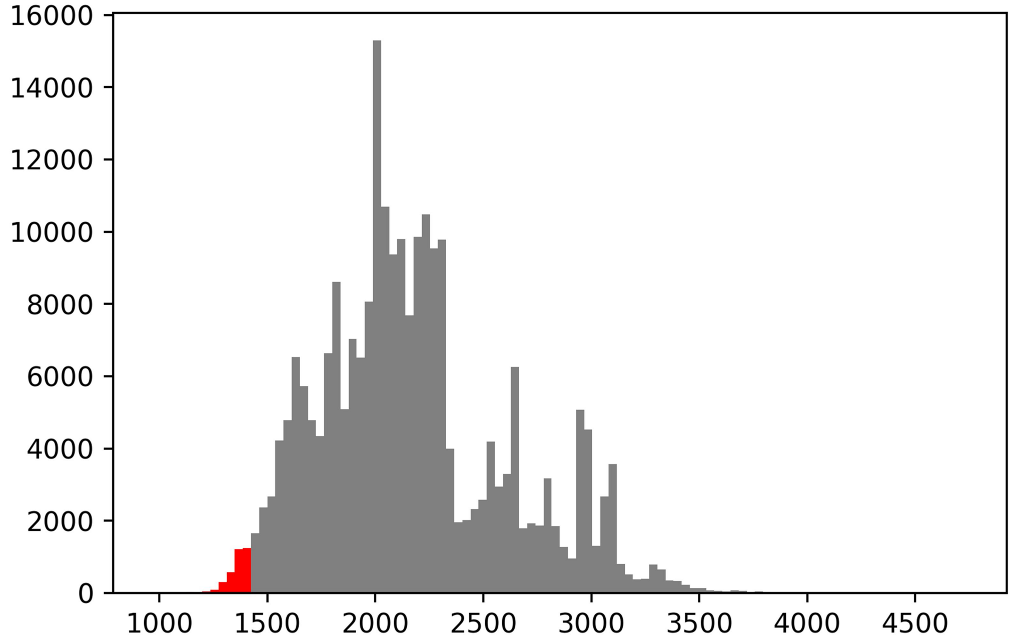


**Supplementary Figure 34: Histogram displaying distances of 239,899 BGC relative to *frs* revealed by a BiG-SliCE analysis.**[10] X-axis displays distance, y-axis number of BGCs. BGCs marked in red (up to d=1400) were selected for a more detailed BiG-SCAPE analysis.[11]

**Supplementary Table 7:** Databank accession numbers and source organisms of protein sequences used for phylogenetic analyses of $C_{starter}$ domains. Related to Fig. 5c and Supplementary Fig. 35.

| Accession | Organism | Accession | Organism |
|---|---|---|---|
| MT876545 | *Chromobacterium vaccinii* MWU205 | WP_084480120.1 | *Methylosarcina lacus* |
| MT876545 | *Chromobacterium vaccinii* MWU205 | WP_078996481.1 | *Lysobacter enzymogenes* |
| KNE75171.1 | *Candidatus* Burkholderia crenata | WP_096414584.1 | *Lysobacter capsici* |
| KNE75168.1 | *Candidatus* Burkholderia crenata | WP_119629200.1 | *Methylocaldum marinum* |
| WP_162850377.1 | *Rhodococcus jostii* | WP_174932260.1 | *Burkholderia lata* |
| WP_007299123.1 | *Rhodococcus imtechensis* | WP_120026908.1 | *Amycolatopsis panacis* |
| RZK84214.1 | *Rhodococcus sp.* | WP_054261810.1 | *Propionispora sp.* 2/2-37 |
| WP_169695704.1 | *Rhodococcus opacus* | WP_091794482.1 | *Lysobacter sp.* yr284 |
| ELB93997.1 | *Rhodococcus wratislaviensis* IFP 2016 | WP_040376786.1 | *Peribacillus psychrosaccharolyticus* |
| TMK21117.1 | *Alphaproteobacteria bacterium* | WP_138885197.1 | *Lysobacter enzymogenes* |
| TMJ40383.1 | *Alphaproteobacteria bacterium* | WP_052756236.1 | *Lysobacter capsici* |
| WP_036714060.1 | *Paenibacillus ehimensis* | KJS54231.1 | *Streptomyces rubellomurinus subsp. indigoferus* |
| WP_176165338.1 | *Streptomyces sp.* NA00687 | WP_166530797.1 | *Agaribacter marinus* |
| WP_106046991.1 | *Bacillus atrophaeus* | TQF01515.1 | *Kitasatospora sp.* MMS16-CNU292 |
| WP_183596885.1 | *Paenibacillus phyllosphaerae* | WP_057921675.1 | *Lysobacter capsici* |
| NQD50526.1 | *Bacillus altitudinis* | WP_049786543.1 | *Mycetohabitans rhizoxinica* |
| WP_156746001.1 | *Mycobacterium sp.* 1423905.2 | CBW76866.1 | *Mycetohabitans rhizoxinica* HKI 454 |
| WP_186229549.1 | *Burkholderia gladioli* | WP_146064026.1 | *Mycetohabitans endofungorum* |
| OKH99041.1 | *Streptomyces sp.* CB02923 | WP_174993913.1 | *Burkholderia arboris* |
| WP_157638970.1 | *Burkholderia ubonensis* | OPD64588.1 | *Lysobacter enzymogenes* |

| | | | |
|---|---|---|---|
| WP_161220653.1 | *Streptomyces sp.* SID6137 | WP_156123907.1 | *Paraburkholderia mimosarum* |
| WP_155634472.1 | *Burkholderia stagnalis* | WP_081052109.1 | *Burkholderia cepacia* |
| REK66978.1 | *Brevibacillus sp.* | WP_026869105.1 | *Inquilinus limosus* |
| APD71879.1 | *Streptomyces sp.* | WP_143200560.1 | *Kitasatospora sp.* CB01950 |
| TDL85614.1 | *Vibrio vulnificus* | SFN60854.1 | *Actinomadura madurae* |
| WP_171565203.1 | *Brevibacillus sp.* MCWH | WP_051213701.1 | *Glomeribacter sp.* 1016415 |
| WP_090670197.1 | *Paenibacillus tianmuensis* | WP_067343588.1 | *Marinomonas spartinae* |
| WP_139641914.1 | *Streptomyces sedi* | WP_169750257.1 | *Streptosporangium amethystogenes* |
| PBC50623.1 | *Rhodococcus sp.* ACS1 | WP_092071365.1 | *Dendrosporobacter quercicolus* |
| WP_161218580.1 | *Streptomyces sp.* SID6139 | WP_045303061.1 | *Saccharothrix sp.* ST-888 |
| WP_135342059.1 | *Streptomyces palmae* | WP_181556777.1 | *Anoxybacillus caldiproteolyticus* |
| WP_155754571.1 | *Burkholderia stagnalis* | WP_034840793.1 | *Inquilinus limosus* |
| WP_161783329.1 | *Burkholderia sp.* A1 | WP_157441225.1 | *Actinoplanes awajinensis* |
| WP_187438659.1 | *Streptomyces sp.* sk2.1 | WP_172236540.1 | *Bradyrhizobium sp.* LMG 8443 |
| AEA62641.1 | *Burkholderia gladioli* BSR3 | WP_143956941.1 | *Mycobacterium sp.* KBS0706 |
| SEC10944.1 | *Rhodococcus koreensis* | WP_165781571.1 | *Streptosporangium minutum* |
| WP_158315302.1 | *Bacillus megaterium* | WP_176072366.1 | *Paraburkholderia mimosarum* |
| WP_072274058.1 | *Peribacillus simplex* | WP_056114189.1 | *Lysobacter sp.* Root690 |
| PAE69549.1 | *Bacillus subtilis* | WP_165940603.1 | *Burkholderia sp.* SRS-46 |
| WP_120709248.1 | *Rhizobium jaguaris* | WP_083780791.1 | *Bradyrhizobium sp.* BTAi1 |
| WP_094237092.1 | *Tumebacillus algifaecis* | WP_067015022.1 | *Marinomonas spartinae* |
| QAR15116.1 | *Streptomyces costaricanus* | PMS18374.1 | *Burkholderia dabaoshanensis* |
| WP_104825816.1 | *Rhizobium sp.* NXC24 | WP_162791553.1 | *Dyella sp.* L4-6 |
| BBA33607.1 | *Methylocaldum marinum* | WP_103564835.1 | *Actinomadura sp.* RB29 |
| WP_090514810.1 | *Paenibacillus sp. cl6col* | RDD80443.1 | *Dyella sp.* L4-6 |
| WP_094237092.1 | *Tumebacillus algifaecis* | WP_146014032.1 | *Burkholderia dabaoshanensis* |
| WP_098680385.1 | *Bacillus altitudinis* | WP_132240709.1 | *Micromonospora sp.* CNZ303 |
| WP_081114287.1 | *Bacillus stratosphericus* | WP_091621489.1 | *Micromonospora peucetia* |
| WP_068017976.1 | *Rhodoplanes sp.* Z2-YC6860 | WP_093292387.1 | *Thermoactinomyces sp.* DSM 45892 |
| WP_181799076.1 | *Kitasatospora sp.* MMS16-CNU292 | WP_184260366.1 | *Granulicella mallensis* |
| WP_061420802.1 | *Bacillus pumilus* | WP_067109092.1 | *Mycobacterium sp.* 852002-51057_SCH5723018 |
| WP_125058029.1 | *Streptomyces rimosus* | WP_110573648.1 | *Marinomonas alcarazii* |
| WP_128788709.1 | *Streptomyces sp. endophyte*_N2 | WP_033674841.1 | *Bacillus gaemokensis* |
| PYS27789.1 | *Acidobacteria bacterium* | WP_172427363.1 | *Streptomyces griseofuscus* |
| WP_048411362.1 | *Chromobacterium sp.* LK1 | WP_157419571.1 | *Actinomadura kijaniata* |
| WP_099396555.1 | *Iodobacter sp.* BJB302 | WP_186145144.1 | *Burkholderia gladioli* |
| AXE32757.1 | *Chromobacterium sp.* IIBBL 112-1 | WP_007952768.1 | *Pelosinus fermentans* |
| WP_082113610.1 | *Chromobacterium vaccinii* | WP_136371369.1 | *Cohnella sp.* CC-MHH1044 |
| WP_122983744.1 | *Chromobacterium subtsugae* | WP_076788272.1 | *Burkholderia sp.* b13 |
| KZE85028.1 | *Chromobacterium sp.* F49 | WP_146012759.1 | *Trinickia caryophylli* |
| SUX53575.1 | *Chromobacterium vaccinii* | WP_146064107.1 | *Mycetohabitans endofungorum* |
| OVE47519.1 | *Chromobacterium violaceum* | WP_142401196.1 | *Mycobacterium marinum* |
| WP_081545507.1 | *Chromobacterium haemolyticum* | WP_067015692.1 | *Mycobacterium sp.* 1081908.1 |
| WP_118266927.1 | *Chromobacterium rhizoryzae* | WP_052407967.1 | *Allokutzneria albata* |
| WP_106076243.1 | *Chromobacterium amazonense* | MPZ86334.1 | *Actinophytocola sp.* |
| WP_107800191.1 | *Chromobacterium sp. Panama* | WP_141996174.1 | *Amycolatopsis cihanbeyliensis* |
| WP_156746001.1 | *Mycobacterium sp.* 1423905.2 | KAK48742.1 | *Caballeronia jiangsuensis* |
| WP_175879783.1 | *Mycobacterium sp.* IS-2888 | WP_099661199.1 | *Sporosarcina sp.* P29 |
| WP_172880913.1 | *Bacillus safensis* | WP_066488029.1 | *Burkholderia sp.* BDU8 |
| WP_097231083.1 | *Streptomyces zhaozhouensis* | WP_073604292.1 | *Vibrio aerogenes* |
| WP_053416733.1 | *Viridibacillus arvi* | SAL02479.1 | *Caballeronia arationis* |
| WP_143665067.1 | *Streptomyces cacaoi* | NVK71858.1 | *Oceanospirillaceae bacterium* |
| WP_123647243.1 | *Lysobacter enzymogenes* | WP_061162849.1 | *Caballeronia temeraria* |
| WP_035299007.1 | *Brevibacillus thermoruber* | SLC21331.1 | *Mycobacteroides abscessus subsp. massiliense* |
| WP_076794250.1 | *Burkholderia sp.* b14 | | |

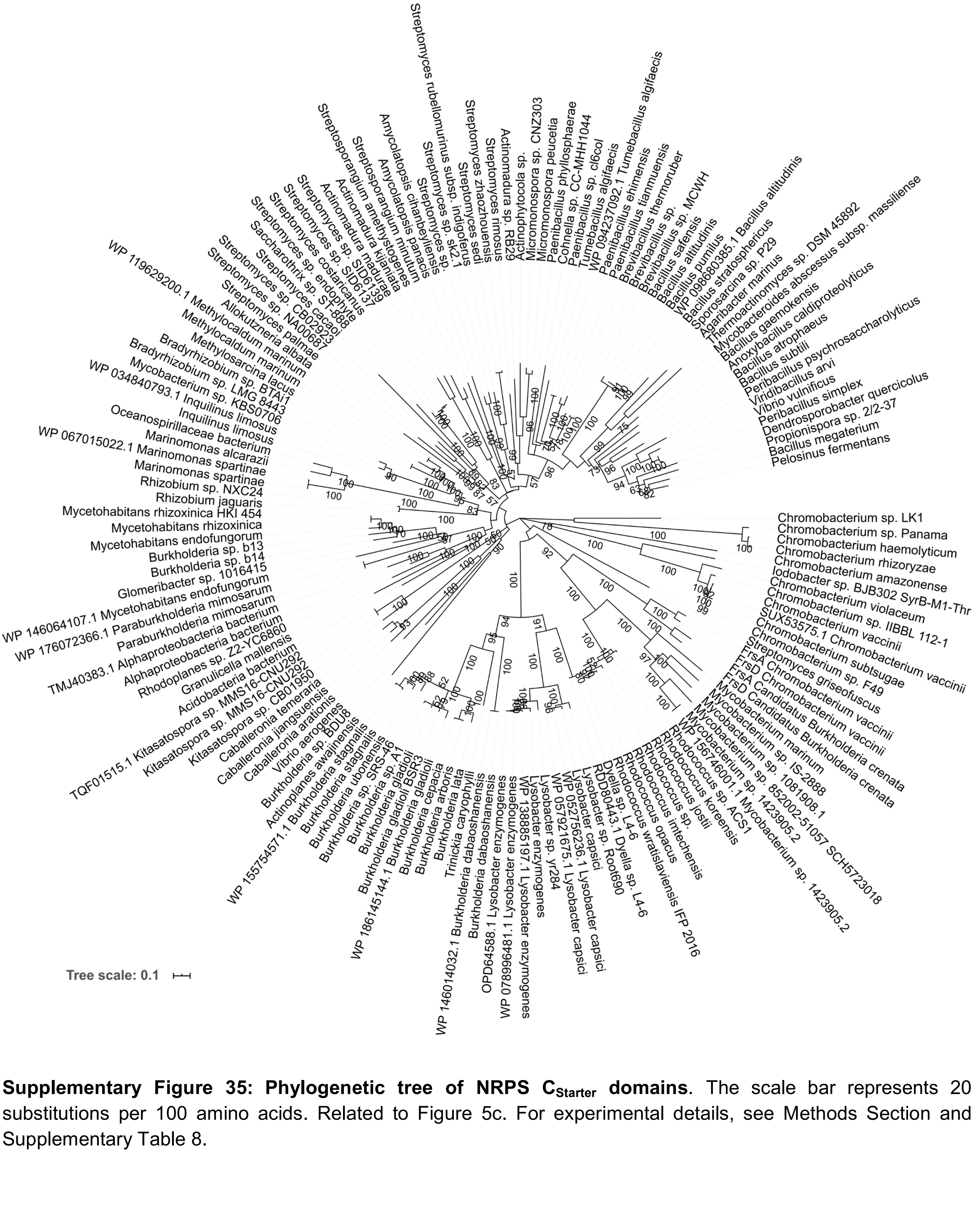


**Supplementary Figure 35: Phylogenetic tree of NRPS $C_{Starter}$ domains**. The scale bar represents 20 substitutions per 100 amino acids. Related to Figure 5c. For experimental details, see Methods Section and Supplementary Table 8.

```
TE_FrsA            1 HPLFCIHPEGGLGWSYIGLALHLDHEQPIYTLQARGLDGMSELAPSIPDM     50
                     .|||||||.|.|.|:|:.|..:||.|||||.|||||:||.||.|.||..|
TE_FrsG            1 PPLFCIHPGGCLSWTYVSLVRYLDAEQPIYGLQARGIDGQSEPASSIEAM     50

TE_FrsA           51 AADYIEQIRSIQPNGPYHLLGWSLGGVIAQEVAVQLERVGEKTALLAILD    100
                     ||||:.|||.|||:|||:||||||||.:||.:|.|||.:.::..||.:||
TE_FrsG           51 AADYVAQIRGIQPHGPYYLLGWSLGGNLAQAMASQLESMDQEVGLLFLLD    100

TE_FrsA          101 TFPIEILHEAMFGKQACAYDLFARVVQEMYLMPIEEARLKSMYLIGLNHM    150
                     :.| ..:|:   ..:...|.||.:..:..:...:.|.::::::.:...|:
TE_FrsG          101 SGP-SPMHK---DDEMIEYPLFTKEFKNTFKFHVSETKMQAIFEVTKRHV    146

TE_FrsA          151 KITAAFSSSHYGGDLLLFRSLIPYAEDALMPEADTWQPYLSGQLEVHDIE    200
                     ::....::....|..||||:.:||.|...:.....|..|:.|.:|||::.
TE_FrsG          147 ELIRQSTTPVSQGPALLFRATVPYDESTPLLPPHAWNEYVKGDIEVHEVH    196

TE_FrsA          201 CTHMDMMQRDVLKIIGPVLESKL    223
                     |.|..|.:.:.::.:|||:|.||
TE_FrsG          197 CQHAQMNRIEFMEQMGPVIERKL    219

                            Length: 223
                  # Identity:      93/223 (41.7%)
                  # Similarity:   139/223 (62.3%)
                  # Gaps:           4/223 (1.8%)
                  # Score: 482.0
```

**Supplementary Figure 36: Emboss Needle Alignment of FrsA$_{TE}$ and FrsG$_{TE}$ from *C. vaccinii*.** Residues in the active site, which are responsible for hydrolysis, are marked in yellow.

**Supplementary Table 8:** Names, databank accession numbers, source organisms of protein sequences used for phylogenetic analyses of TE domains. Related to Fig. 5d.

| Name | Accession | Organism |
|---|---|---|
| BcFrsA-TE | KNE75171.1 | *"Candidatus* Burkholderia crenata" |
| BcFrsG-TE | KNE75165.1 | *"Candidatus* Burkholderia crenata" |
| cvFrsA-TE | MT876545 | *Chromobacterium vaccinii* MWU205 |
| cvFrsG-TE | MT876545 | *Chromobacterium vaccinii* MWU205 |
| DhbF - TE | WP_144530663.1 | *Bacillus subtilis* ATCC 21332 |
| EntF - TE | AYG20286.1 | *Escherichia coli str.* K-12 *substr.* MG1655 |
| ERYA3_TE | Q03133 | *Saccharopolyspora erythraea* |
| FtdB - TE | ADJ54381.1 | *Streptomyces sp.* SPB78 |
| HbnA | ALV82446.1 | *Streptomyces variabili* |
| HSAF - TE | ABL86391.1 | *Lysobacter enzymogenes* |
| IkaA - TE | AJD77023.1 | *Streptomyces sp.* ZJ306 |
| KirHI | AGS67165.1 | *Streptomyces collinus* Tu 365 |
| KSE_70420 | BAJ32800 | *Kitasatospora setae* KM-6054 |
| LipX2 | ABB05100 | *Streptomyces aureofaciens* |
| LybB - TE1 | AEH59100.1 | *Lysobacter sp.* ATCC 53042 |
| LybB - TE2 | AEH59100.1 | *Lysobacter sp.* ATCC 53042 |
| MassC - TE1 | EIK63041.1 | *Pseudomonas fluorescens* SS101 |
| MassC - TE2 | EIK63041.1 | *Pseudomonas fluorescens* SS101 |
| NocB - TE | AAT09805.1 | *Nocardia uniformis subsp. tsuyamanensis* |
| ObiF - TE | KX134687.1 | *Pseudomonas fluorescens* ATCC 39502 |
| Pys-Pent - TE | WP_011533552 | *Pseudomonas entomophila* |
| Pys-Pflu -TE | WP_064118616 | *Pseudomonas fluorescens* HKI 0770 |
| RomH | WP_078586793.1 | *Streptomyces rimosus* NRRL B-2659 |
| RomI | WP_004571777.1 | *Streptomyces rimosus* NRRL B-2659 |
| SGR814 - TE | AGK81502 | *Streptomyces fulvissimus* DSM 40593 |
| SlgL | CBA11558 | *Streptomyces lydicus* |
| Sln6 - TE | DAB41476.1 | *Streptomyces sp.* CNB-091 |
| Sln9 - TE | DAB41479.1 | *Streptomyces sp.* CNB-091 |
| SrfA-C - TE | 2VSQ | *Bacillus subtilis* ATCC 21332 |
| SSHG - TE | EFE85271.1 | *Streptomyces albus* J1074 |
| SwrW - TE | I7GF64 | *Serratia marcescens* |

| | | |
|---|---|---|
| TaaE - TE1 | CCJ67640.1 | *Pseudomonas costantinii* DSM 16734 |
| TaaE - TE2 | CCJ67640.1 | *Pseudomonas costantinii* |
| TrdC | ADY38535.1 | *Streptomyces sp.* SCSIO 1666 |
| TycC - TE | AAC45930.1 | *Brevibacillus brevis* |
| ViscC - TE1 | CAY48789.1 | *Pseudomonas fluorescens* SBW25 |
| ViscC - TE2 | CAY48789.1 | *Pseudomonas fluorescens* SBW25 |
| WlipC - TE1 | AFJ23826.1 | *Pseudomonas putida* |
| WlipC - TE2 | AFJ23826.1 | *Pseudomonas putida* |

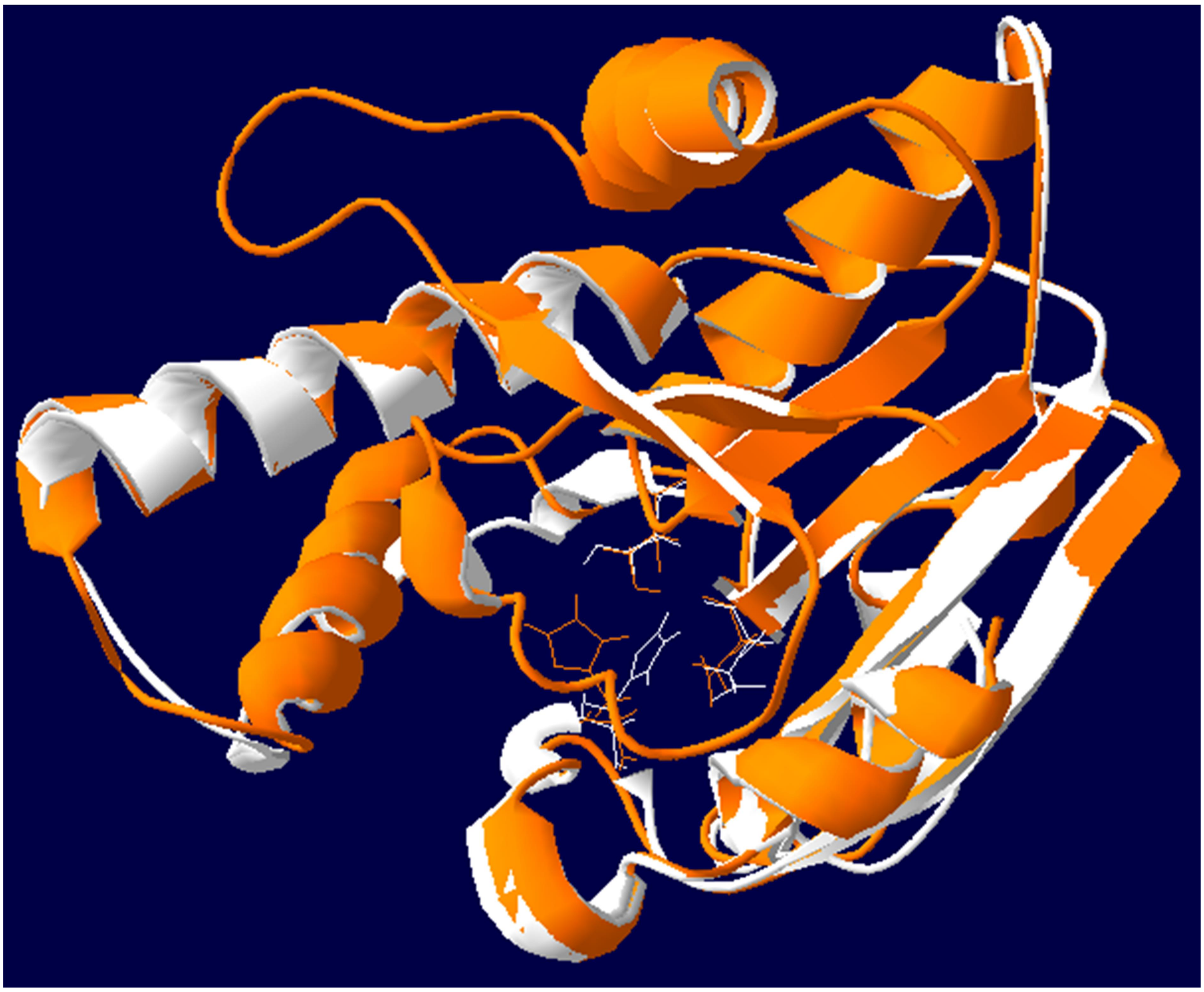

**Supplementary Figure 37: Alignment of the structural models of FrsA$_{TE}$ (orange) and FrsG$_{TE}$ (white) from *C. vaccinii*.** The amino acids of the active site are displayed in red (FrsA) and blue (FrsG).

# Discussion: FrsA$_{TE}$ acceptor substrate specificity

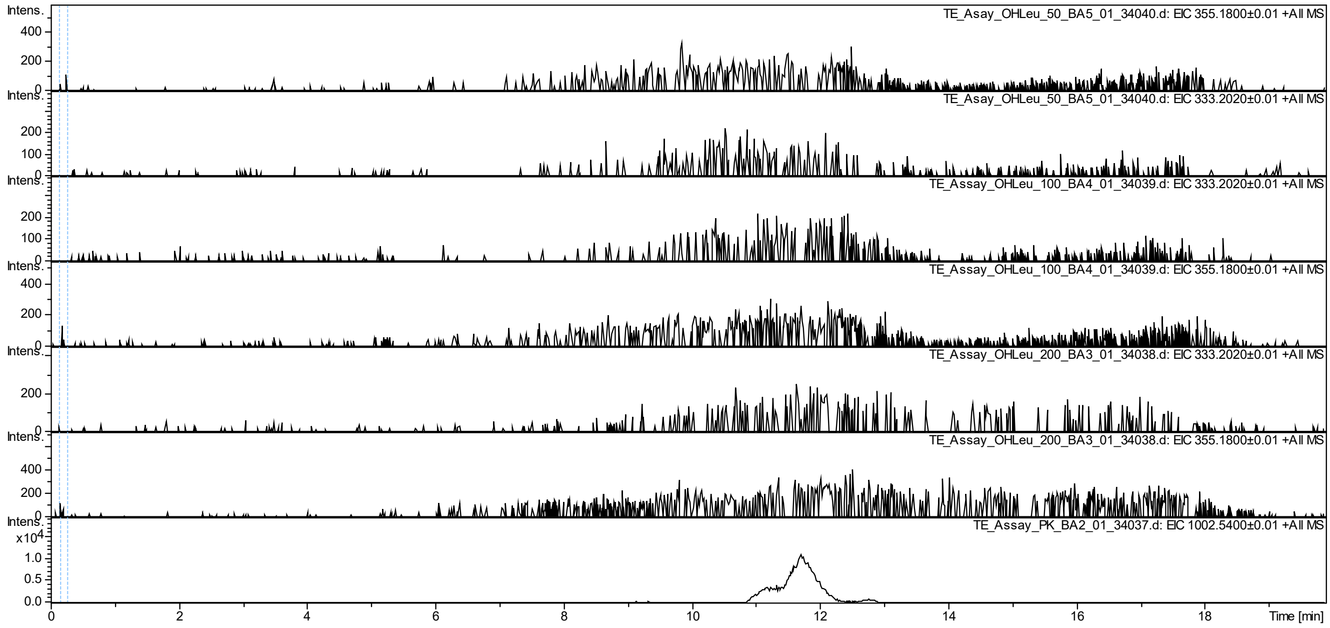


**Supplementary Figure 38:** EICs of HPLC-MS experiments of transesterification assays with the added minimal substrate Hle in varying concentrations (50 µM, 100 µM, 200 µM), instead of **2**. EIC for the proton adduct (*m/z*: 333.202) and the sodium adduct (*m/z*: 355.180) of the anticipated product Hle-N-Pp-Hle ester were generated. Also, the data was analyzed for formation of dimer and trimer esters. No product formation could be observed. FrsA TE was reconstituted in active conformation in this assay, as can be seen from the positive control experiment in the bottom chromatogram (formation of **1** after addition of **2**).

## Supplementary References


[1] Carlier, A. et al. The genome analysis of Candidatus Burkholderia crenata reveals that secondary metabolism may be a key function of the Ardisia crenata leaf nodule symbiosis. *Environ. Microbiol.* **18,** 2507–2522 (2016).

[2] Madeira, F. et al., The EMBL-EBI search and sequence analysis tools APIs in 2019. *Nucleic Acids Res.* **47,** W636–W641 (2019).

[3] Hoang, T. T., Karkhoff-Schweizer, R. R., Kutchma, A. J., Schweizer, H. P. A broad-host-range Flp-FRT recombination system for site-specific excision of chromosomally-located DNA sequences: application for isolation of unmarked Pseudomonas aeruginosa mutants. *Gene* **212,** 77–86 (1998).

[4] Norrander, J., Kempe, T., Messing, J. Construction of improved M13 vectors using oligodeoxynucleotide-directed mutagenesis. *Gene* **26,** 101–106 (1983).

[5] Gibson, D. G., Young, L., Chuang, R. Y., Venter, J. C., Hutchison 3rd, C. A., Smith, H. O. Enzymatic assembly of DNA molecules up to several hundred kilobases. *Nat. Methods* **6,** 343–345 (2009).

[6] Lynch, M. D., Gill, R. T. Broad host range vectors for stable genomic library construction. *Biotechnol. Bioeng.* **94,** 151–158 (2006).

[7] Wang, C., Wesener, S. R., Zhang, H. Cheng, Y.-Q. An FAD-dependent pyridine nucleotide-disulfide oxidoreductase is involved in disulfide bond formation in FK228 anticancer depsipeptide. *Chem. Biol*. **16,** 585-593 (2009).

[8] Broetto, L., Cecagno, R., Sant'Anna, F. H., Weber, S., Schrank, I. S. Stable transformation of Chromobacterium violaceum with a broad-host-range plasmid. *Appl. Microbiol. Biotechnol.* **71,** 450–454 (2006).

[9] Reher, R. et al. Applying molecular networking for the detection of natural sources and analogues of the selective Gq Protein inhibitor FR900359. *J Nat. Prod.* **81,** 1628–1635 (2018).

[10] Kautsar, S.A., van der Hooft, J.J.J., de Ridder, D., Medema, M.H. BiG-SLiCE: A highly scalable tool maps the diversity of 1.2 million biosynthetic gene clusters. *bioRxiv* (2020).

[11] Navarro-Muñoz, J. C., et al. A computational framework to explore large-scale biosynthetic diversity. *Nat. Chem. Biol.* **16,** 60-68 (2020).
