# Supplementary material for: Thioesterase-mediated side chain transesterification generates potent Gq signaling inhibitor FR900359
Source: Nat Commun. 2021 Jan 8;12:144. doi: 10.1038/s41467-020-20418-3 (PMC7794379; doi:10.1038/s41467-020-20418-3)
Supplement: Supplementary file 2 — Description of Additional Supplementary Files [file 41467_2020_20418_MOESM2_ESM.pdf]

<u>Description of Additional Supplementary Files</u>

Supplementary Data 1: List of BGCs extracted from the BiG-FAM database (distance cutoff=1400) used for the BiG-SCAPE analysis. Dataset type, genome and BGC accession codes as well as BiG-SLiCE distance to *frs* are indicated.
