# Supplementary material for: Thioesterase-mediated side chain transesterification generates potent Gq signaling inhibitor FR900359
Source: Nat Commun. 2021 Jan 8;12:144. doi: 10.1038/s41467-020-20418-3 (PMC7794379; doi:10.1038/s41467-020-20418-3)
Supplement: Supplementary file 4 — Reporting Summary [file 41467_2020_20418_MOESM4_ESM.pdf]

nature research

Corresponding author(s): Max Crüsemann

Last updated by author(s): Nov 6, 2020

Reporting Summary

Nature Research wishes to improve the reproducibility of the work that we publish. This form provides structure for consistency and transparency in reporting. For further information on Nature Research policies, see our Editorial Policies and the Editorial Policy Checklist.

Statistics

For all statistical analyses, confirm that the following items are present in the figure legend, table legend, main text, or Methods section.

n/a	Confirmed	
☐	☒	The exact sample size (*n*) for each experimental group/condition, given as a discrete number and unit of measurement
☒	☐	A statement on whether measurements were taken from distinct samples or whether the same sample was measured repeatedly
☒	☐	The statistical test(s) used AND whether they are one- or two-sided<br>*Only common tests should be described solely by name; describe more complex techniques in the Methods section.*
☒	☐	A description of all covariates tested
☒	☐	A description of any assumptions or corrections, such as tests of normality and adjustment for multiple comparisons
☐	☒	A full description of the statistical parameters including central tendency (e.g. means) or other basic estimates (e.g. regression coefficient) AND variation (e.g. standard deviation) or associated estimates of uncertainty (e.g. confidence intervals)
☒	☐	For null hypothesis testing, the test statistic (e.g. *F*, *t*, *r*) with confidence intervals, effect sizes, degrees of freedom and *P* value noted<br>*Give P values as exact values whenever suitable.*
☒	☐	For Bayesian analysis, information on the choice of priors and Markov chain Monte Carlo settings
☒	☐	For hierarchical and complex designs, identification of the appropriate level for tests and full reporting of outcomes
☒	☐	Estimates of effect sizes (e.g. Cohen's *d*, Pearson's *r*), indicating how they were calculated

Our web collection on statistics for biologists contains articles on many of the points above.

Software and code

Policy information about availability of computer code

Data collection	We used no specific software to collect our data.
Data analysis	We used the following published software and web-based analysis tools to analyze our data: MestreNova8, Bruker Data analysis 4.2, Cytoscape 3.8, MUSCLE 3.8.31, MEGA 10, GraphPad PRISM 7 and 8, AutoDock 4.2, EMBOSS-NEEDLE (https://www.ebi.ac.uk/Tools/psa/emboss_needle), GNPS (gnps.ucsd.edu, Ref. 31), I-TASSER (https://zhanglab.ccmb.med.umich.edu/I-TASSER/ Refs. 65,66), BiG-SLiCE (Ref. 44), BiG-SCAPE (Ref. 45)

For manuscripts utilizing custom algorithms or software that are central to the research but not yet described in published literature, software must be made available to editors and reviewers. We strongly encourage code deposition in a community repository (e.g. GitHub). See the Nature Research guidelines for submitting code & software for further information.

Data

Policy information about availability of data

All manuscripts must include a data availability statement. This statement should provide the following information, where applicable:

- Accession codes, unique identifiers, or web links for publicly available datasets
- A list of figures that have associated raw data
- A description of any restrictions on data availability

frs sequences have been deposited at GenBank, accession number MT876545. MS/MS data is deposited at MassIVE (massive.ucsd.edu), dataset MSV000085975. The molecular network can be found under: gnps.ucsd.edu/ProteoSAFe/status.jsp?task=8e524f93dc66405b98c9d77cc0f9c596. The Gq protein structure was retrieved from rcsb.org/structure/3AH8. NRPS BGCs were downloaded from the BiG-FAM database (bigfam.bioinformatics.nl/home). BGCs used for the BiG-SCAPE analysis are listed in Supplementary data 1. Source data for Figure 4 a,b,c,e are provided with this paper. All other data are available from the corresponding author upon request.

Field-specific reporting

Please select the one below that is the best fit for your research. If you are not sure, read the appropriate sections before making your selection.

- [x] Life sciences
- [ ] Behavioural & social sciences
- [ ] Ecological, evolutionary & environmental sciences

For a reference copy of the document with all sections, see nature.com/documents/nr-reporting-summary-flat.pdf

Life sciences study design

All studies must disclose on these points even when the disclosure is negative.

Sample size	Sample size: 7 groups of each 20 animals were treated with control (1 group) and two different compounds at 3 different concentrations (6 groups). No sample size calculation was performed. Sample size (size of group) was determined based on standard laboratory practice, and the available amount of the compounds for the experiments.
Data exclusions	No data were excluded from the analyses.
Replication	Experiments was performed twice with identical results.
Randomization	ithin an experiment (replicate), all animals (freshly hatched first instars) were issued from the same container, born on the same day and randomly divided in 7 animal groups.
Blinding	No blinding of the groups was performed. The outcome measure of the experiment was the daily determination of the live/dead status of each individual animal which is on objective and binary criterium (yes/no).

Reporting for specific materials, systems and methods

We require information from authors about some types of materials, experimental systems and methods used in many studies. Here, indicate whether each material, system or method listed is relevant to your study. If you are not sure if a list item applies to your research, read the appropriate section before selecting a response.

Materials & experimental systems

n/a	Involved in the study	
x		Antibodies
	x	Eukaryotic cell lines
x		Palaeontology and archaeology
	x	Animals and other organisms
x		Human research participants
x		Clinical data
x		Dual use research of concern

Methods

n/a	Involved in the study	
x		ChIP-seq
x		Flow cytometry
x		MRI-based neuroimaging

Eukaryotic cell lines

Policy information about cell lines

Cell line source(s)	human HEK293, genome edited for the GNAQ and the GNA11 genes, established by and obtained from Asuka Inoue, coauthor of this study.
Authentication	The GNAQ/GNA11-deficient HEK293 cell line was authenticated by a restriction enzyme-based genotyping method in the Inoue lab. After transferring the cells to the Crüsemann lab, the cell line was not authenticated.
Mycoplasma contamination	All cell lines were tested negative for mycoplasma contamination.
Commonly misidentified lines (See ICLAC register)	No commonly misidentified cell lines were used in this study.

Animals and other organisms

Policy information about studies involving animals; ARRIVE guidelines recommended for reporting animal research

Laboratory animals	Insects of the species Riptortus pedestris were used in this study. ARRIVE guidelines were taken into account when planning, conducting and reporting the animal experiments in this study

Wild animals	*Provide details on animals observed in or captured in the field; report species, sex and age where possible. Describe how animals were caught and transported and what happened to captive animals after the study (if killed, explain why and describe method; if released, say where and when) OR state that the study did not involve wild animals.*
Field-collected samples	*For laboratory work with field-collected samples, describe all relevant parameters such as housing, maintenance, temperature, photoperiod and end-of-experiment protocol OR state that the study did not involve samples collected from the field.*
Ethics oversight	*Identify the organization(s) that approved or provided guidance on the study protocol, OR state that no ethical approval or guidance was required and explain why not.*

Note that full information on the approval of the study protocol must also be provided in the manuscript.
